# Effectiveness of the Essential Critical Care Concepts in Emergency Medicine: Extracorporeal Membrane Oxygenation and Cardiovascular Devices Module Implementation

**DOI:** 10.15766/mep_2374-8265.11556

**Published:** 2025-11-07

**Authors:** Matthew M. T. Carvey, Daniel M. Zumsteg, Ava A. Omidvar, Arya Hawkins-Zafarnia, Allyson M. Hynes

**Affiliations:** 1 Fellow, Department of Anesthesiology, Cleveland Clinic Foundation; 2 Resident, Department of Emergency Medicine, Medical College of Wisconsin; 3 Resident, Department of Emergency Medicine, University of Maryland; 4 Resident, Department of Emergency Medicine, Yale School of Medicine; 5 Assistant Professor, Department of Surgery, Division of Trauma and Acute Care Surgery, Medical College of Wisconsin; Assistant Professor, Department of Emergency Medicine, Medical College of Wisconsin

**Keywords:** Extracorporeal Membrane Oxygenation, Flipped Classroom, Cardiovascular Medicine, Critical Care Medicine, Emergency Medicine

## Abstract

**Introduction:**

In the emergency department, emergency medicine (EM) physicians should be familiar with advanced cardiovascular devices, such as extracorporeal membrane oxygenation (ECMO), intra-aortic balloon pumps (IABPs), and resuscitative endovascular balloon occlusion of the aorta (REBOA) catheters. Therefore, we developed a module to help learners integrate these modalities in the acute care setting to enhance patient care.

**Methods:**

Third- and fourth-year medical students interested in EM, as well as EM interns, participated in the module during their Introduction to Critical Care in EM course. The session involved a 60-minute flipped classroom online curriculum. Students received prerecorded lectures and PDF materials before the session. Students had to pass a premodule knowledge quiz and complete a postmodule quiz using 5-point Likert scales (1 = *strongly disagree*, 5 = *strongly agree*) assessing the module's effectiveness.

**Results:**

Thirty students provided overwhelmingly positive feedback, indicating that the module effectively taught the basics of advanced devices and how the devices apply to EM and critical care (each, median score 5). Participants expressed increased confidence in managing IABPs, ECMO, and REBOA (each, median score 5). Free-text comments highlighted that the material was challenging but helpful. Students’ premodule median knowledge score was 2 (interquartile range [IQR] 2–3), versus a median score of 4 (IQR 4–4) postmodule (*p* < .001).

**Discussion:**

This advanced cardiovascular device module offers a structured approach to teaching ECMO, IABP, and REBOA cardiovascular management to students interested in EM. Our module effectively addressed the educational gap and helped students achieve the learning objectives.

## Educational Objectives

By the end of this activity, learners will be able to:

1. Identify the indications, contraindications, mechanics, and complications behind veno-venous (VV) and veno-arterial (VA) extracorporeal membrane oxygenation (ECMO) therapy and cannulation.
2. Identify the indications, contraindications, mechanics, and complications behind intra-aortic balloon pump (IABP) therapy.
3. Identify the indications, contraindications, mechanics, and complications behind resuscitative endovascular balloon occlusion of the aorta (REBOA) therapy and cannulation.
4. Describe the difference between VV-ECMO and VA-ECMO, and specify the clinical scenarios that warrant each specific form of ECMO.
5. Demonstrate the ability to understand IABP inflation and deflation waveforms, as well as corrective techniques to avoid timing errors.

## Introduction

The use of extracorporeal membrane oxygenation (ECMO), intra-aortic balloon pumps (IABPs), and other cardiovascular devices for critically ill patients in the emergency department (ED) has increased over time. Emergency physicians (EPs) are increasingly initiating ECMO, with studies showing improved survival after extracorporeal cardiopulmonary resuscitation.^1^ ECMO usage is expected to increase, as the Extracorporeal Life Support Organization reports a significant rise in implementation of veno-venous ECMO (VV-ECMO) therapy since 2009, with more than 6000 registered cases in just over the first 6 months of 2017 and an expected continuing rise in cases.^2^ It is known that early IABP initiation in cardiogenic shock is also linked to lowered lactate levels and better outcomes.^3^ Similarly, the use of resuscitative endovascular balloon occlusion of the aorta (REBOA) to prevent re-arrest after return of spontaneous circulation in out-of-hospital cardiac arrest scenarios is becoming more common, with some studies supporting its effectiveness.^4^ An analysis of 3398 REBOA procedures from 2017 to 2022 found that REBOA usage remained limited but steady, primarily occurring at level 1 trauma center EDs.^5^

Despite the increased use of these procedures in the ED, familiarity and comfort with them are still lacking. As ECMO usage in the ED is expected to rise, understanding its management is becoming crucial.^6^ It is essential for EPs to recognize the indications and contraindications for ECMO, become familiar with cannulation, and understand the differences between VV-ECMO and veno-arterial ECMO (VA-ECMO). Additionally, with the increasing prevalence of prehospitalization IABP management, EPs are also encountering these devices more frequently in the ED.

In a retrospective analysis conducted over a 7-year period, Bur and colleagues evaluated 88 patients who received IABP counter-pulsation for cardiogenic shock in a single ED. Management by both EPs and cardiologists was associated with potentially improved clinical outcomes.^3^ Similarly, as REBOA indications have expanded and REBOA cases have increased, maintaining knowledge of REBOA is vital, particularly since prior work has detailed that understanding of REBOA deteriorates without regular clinical cases.^7^ We recognize that ECMO, IABP, and REBOA cardiovascular management procedures are commonly introduced during residency or fellowship through structured didactics, simulation training, and supervised clinical exposure. Early familiarization, particularly through brief, guided learning sessions, may serve as a valuable strategy to scaffold understanding of translational cardiovascular principles, potentially demystifying complex procedures and preparing learners for more advanced training. Moreover, with ECMO increasingly being used in EDs, the American Board of Emergency Medicine has made it a core certification topic in the 2022 Model of the Clinical Practice of Emergency Medicine, making understanding its fundamentals essential for those wishing to pursue a career in emergency medicine (EM).^8^

An advanced cardiovascular devices module was created for third- and fourth-year medical students and EM interns to integrate these procedures into practice as eventual EPs. This 60-minute flipped classroom module teaches the fundamentals of ECMO, IABPs, and other cardiovascular devices early in medical education to foster interest and understanding. It challenges students to grasp the pathophysiology, indications, contraindications, mechanics, and complications behind these life-stabilizing interventions, further solidified through complex case discussions and with emphasis placed on their growing relevance in the ED. A review of *MedEdPORTAL* and PubMed revealed no publications describing a definitive or widely adopted instructional module that is specifically designed to teach medical students the management of life-threatening cardiovascular conditions using advanced support devices, including use of ECMO, IABPs, or REBOA.

## Methods

This educational module was primarily designed to engage third- and fourth-year medical students, as well as EM interns, through an international EM academy.^9^ We intentionally excluded senior EM residents, as this module was part of a larger introductory critical care course and, overall, would be too basic for their level of training. The initiative was designed to introduce advanced cardiovascular device concepts early in the medical curriculum, offering learners repeated exposure to these complex topics and reinforcing a strong foundational understanding from the outset of their training. Prior to its launch, the module underwent a rigorous review process led by content experts in EM and critical care from the Critical Care Medicine Section of the American Academy of Emergency Medicine. This comprehensive evaluation ensured the module's clinical accuracy and alignment with evidence-based medicine principles.

The development process commenced with a targeted literature review to ground the material in the latest evidence-based practices. Subsequently, clinical case scenarios were crafted based on the authors’ practical experiences, ensuring both realism and applicability. Over 2 years, the development process was iterative, incorporating multiple rounds of drafting, expert peer review, and refinement, thereby reinforcing the educational rigor and relevance of the module. According to the University of Vermont Health Network, the proposed activity did not meet the federal regulatory definition of research that would require institutional review board (IRB) review and approval (University of Vermont Health Network IRB reference No. 192.240.43.189).

To ensure students could build effectively upon prior knowledge, a prerequisite for module participation was a working familiarity with cardiovascular anatomy and physiology. This assumption was based on the educational principle that solid grounding in the basic sciences enables learners to better grasp complex clinical conditions and interventions in a more integrated and meaningful way.^10^

The module unfolded in a structured sequence that began with a recorded presentation and culminated in a flipped classroom online session and postcourse evaluation. The only notable cost associated with the module was the use of a webinar platform to host the live sessions. Attendance at the online session was required unless prior arrangements had been made, and participants were expected to complete both pre- and postmodule assessments and a feedback survey.

The course was facilitated by experienced educators, including a senior EM resident and a physician dually trained in EM and critical care. The educational format centered on a single, 60-minute flipped classroom online session, designed to encourage interactive, large-group discussions around clinical cases. Learners were expected to come prepared, having already reviewed the recorded presentation and completed the premodule knowledge quiz.

The structure of the module is outlined in detail in the facilitator guide (Appendix A) and included the following components:

### Introduction and Objectives

The session began with a brief, 2-minute introduction by the facilitators, followed by an overview of the learning objectives, delivered via a prerecorded PowerPoint presentation (Appendix B).

### Prerecorded Module Presentation

The main educational content was delivered through a 41-minute prerecorded PowerPoint lecture, which was made accessible to students at the beginning of the week. This presentation covered advanced cardiovascular devices, with specific focus on ECMO, IABPs, and REBOA (Appendices C and D).

### Online Module Quiz

Following the lecture, students completed a five-question postmodule quiz designed in a case-based format to assess their understanding of the module content (Appendix E). Topics included the function and clinical application of ECMO, IABPs, and REBOA. A minimum passing score of 80% was required. Students who did not pass were offered remediation sessions with the course directors to review key concepts before retaking the quiz. Remediation sessions were formative rather than punitive, offering 15–20 minutes of supportive dialogue to reinforce module comprehension by reviewing incorrect quiz responses and addressing student questions. Students were permitted to retake the quiz only after both they and the course director confirmed adequate understanding.

### Flipped Classroom Online Session and Case Discussions

Once the prerecorded materials and quiz were completed, students participated in a 60-minute live online session. This session focused on case-based discussions and applied learning. One case presented was a patient with acute respiratory failure due to acute respiratory distress syndrome (ARDS) in the context of septic shock. Learners explored the diagnostic criteria for ARDS, ventilator strategies, and the role of VV-ECMO, including its use as a bridge to transplant or recovery.^11^ Additional questions were posed to deepen understanding of the differences between ECMO, IABP, and REBOA. Discussions included recognizing IABP timing errors and comparing cannulation techniques for VV-ECMO versus VA-ECMO, all of which were supported by the material provided in the facilitator guide (Appendix A).

### Postmodule Assessments and Survey

To evaluate the module's effectiveness, participants completed a postmodule quiz identical to the premodule version (Appendix E) assessing students’ understanding of advanced cardiovascular devices, with answer keys provided for evaluation purposes (Appendix F). The assessments were designed to measure knowledge acquisition through a series of multiple-choice, case-based questions. These tools were piloted with two student cohorts prior to being finalized for broader use. Understanding and confidence were assessed using 5-point Likert scales, with responses ranging from 1 = *strongly disagree* to 5 = *strongly agree* (Appendix G). In addition, a postmodule survey was distributed to collect qualitative and quantitative feedback on the module's relevance, content clarity, and the learners’ perceived confidence in managing advanced cardiovascular devices.

## Results

Since the introduction of this advanced cardiovascular devices module, a total of 30 medical students and EM interns with no prior critical care experience completed the finalized module and were included in the outcome analysis, with the sample size pragmatically determined based on instructor-to-student ratio and the need to ensure equal opportunity for participation within the flipped classroom learning model. The module was piloted with 58 medical students for 2 years prior to its initiation, and all feedback collected during this time was used to finalize the current curriculum. As represented in the Table, the questionnaire results showed favorable feedback with regard to the module. The majority of respondents strongly agreed that the module effectively taught the basics of advanced cardiovascular devices (median Likert score 5). Most learners found that the devices taught in the module apply directly to EM and critical care (median Likert score 5). Furthermore, participants expressed increased confidence in managing IABPs (median Likert score 5), ECMO (median Likert score 5), and REBOA (median Likert score 5) after participation in the module.

**Table.**
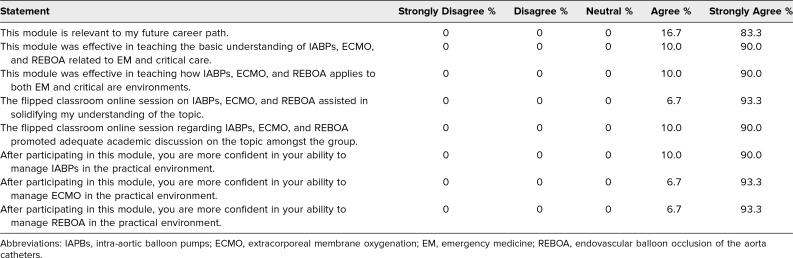
Extracorporeal Membrane Oxygenation and Cardiovascular Devices Postmodule Questionnaire Assessing Module Effectiveness and Student Confidence (*N* = 30)

We invited student feedback through a questionnaire to help improve the course for future participants. Responses were positive, with one student describing the module as “challenging and helpful” and another student stating, “Best module I've had on this.”

A five-question pre- and postmodule quiz determined students’ understanding before the module and the overall effect of the module on the student's understanding and retention of the material at its conclusion. The students’ median premodule knowledge quiz score was 2 (interquartile range [IQR] 2–3) and their median postmodule knowledge quiz score was 4 (IQR 4–4; *p* < .001). Analysis of pre- and postmodule quiz scores demonstrates a statistically significant improvement in students’ understanding of cardiovascular topics. This substantial and consistent gain in performance indicates that students not only engaged with the material but also retained and applied the knowledge effectively, confirming the module's educational impact.

## Discussion

The module was successful in exposing medical students to ECMO, IABPs, and other advanced cardiovascular devices, allowing an early introduction to critical care topics through case-based discussions and flipped classroom learning. All students reported a positive educational experience, based on the results of the questionnaire. The overwhelmingly positive reception indicates that the module's approach to understanding advanced cardiovascular topics is an appropriate method for closing the curricular gap identified in medical education. It provided a learning opportunity to challenge students’ current physiologic understanding from a medical school perspective and apply it practically to cardiovascular devices.

The learning material for this module was specifically designed for use by medical students interested in EM and EM interns; however, it could also be used by other learners, including internal medicine or surgical residents. The flipped classroom presentation and case-based discussions were delivered by two facilitators: a senior EM resident presented the material, while an intensivist assisted with developing the module's content and delivery. PowerPoint was used to create the teaching content. The flipped classroom session and case-based discussions were delivered online to ensure inclusion of students from international locations; however, it could be readily implemented at the local, regional, or national level. Additionally, other means (eg, in-person sessions at an international conference) could also be used to present the material.

This module was created to address a gap in medical curricula: early access to cardiovascular critical care education. During the pilot phase of this course with 58 medical students, we found that third- and fourth-year students had a better understanding of the pathophysiology of cardiovascular devices than second-year medical students. As a result, we identified our primary audience as senior medical students rather than first- and second-year students, who may lack the educational foundation necessary to fully grasp these critical care concepts. Early on, we discovered that the mechanical and physiologic complexity of ECMO and IABP therapy posed a barrier to understanding. Therefore, we broke these devices into multiple sections and explained them in a stepwise manner, ultimately leading to a comprehensive understanding of the device as a whole upon completion.

Common comments from learners included, “I'm glad I had exposure to these cardiovascular devices early in my medical education,” and “I thought these critical care topics would be too difficult for me to understand as a medical student.” This underscores the opportunity for medical educators to implement strategies that enhance understanding of challenging material, rather than assuming it is only suitable for advanced learners with a more substantial foundation (eg, critical care fellows or senior residents).

As these cardiovascular devices become more frequent in nontraditional settings, such as the ED, it is increasingly important for those interested in EM to understand their utility and practical applications. For example, ECMO is increasingly used in resuscitation, or early extracorporeal life support. Kraai et al conducted a case series showing improved survival and favorable neurologic outcomes with intensivist-deployed extracorporeal cardiopulmonary resuscitation in cases of cardiac arrest due to accidental hypothermia. Some of the authors of that study were credentialed ECMO cannulators, and 6 of the 8 were EM trained.^12^ Kraai and colleagues also developed a model for initiating and developing early extracorporeal life support programs in settings with limited access to local surgical services, demonstrating successful outcomes with intensivist-led cannulations.^13^ This further highlights the importance of understanding advanced cardiovascular devices as they become integrated into low-resource settings and EDs.

A limitation of this educational experience is that our postmodule evaluation was administered immediately following the session, which did not account for long-term knowledge retention or efficacy. Second, the virtual format of the module may have impacted learner engagement by limiting hands-on practice, real-time feedback, and peer interaction. While virtual delivery offers flexibility, future iterations may benefit from hybrid or in-person formats to support experiential learning. Third, generalizability may be limited, as the module was tailored to the ED environment specifically. Its applicability to other settings, such as the ICU—with differing learner populations, resources, and device usage—remains uncertain. Broader implementation studies are needed to assess adaptability across contexts.

Overall, participants reported an increased understanding of advanced cardiovascular devices, addressing an educational gap identified in medical curricula. Moving forward, we aim to continue developing this curriculum and objectively assess learning outcomes, long-term knowledge retention, and student comfort with advanced cardiovascular devices through 3- and 6-month evaluations. We would also like to expand our content to early trainees within other fields who have a critical care interest.

## Appendices


Facilitator Guide - ECMO and ACD.docxLearning Objectives - ECMO and ACD.docxModule Presentation Slides - ECMO and ACD.pptxModule Presentation Recording - ECMO and ACD.mp4Module Quiz - ECMO and ACD.docxModule Quiz Answers - ECMO and ACD.docxPostmodule Survey Likert Questions.docx
*All appendices are peer reviewed as integral parts of the Original Publication.*
