# Supplementary material for: Effectiveness of the Essential Critical Care Concepts in Emergency Medicine: Extracorporeal Membrane Oxygenation and Cardiovascular Devices Module Implementation
Source: MedEdPORTAL. 2025 Nov 7;21:11556. doi: 10.15766/mep_2374-8265.11556 (PMC12592219; doi:10.15766/mep_2374-8265.11556)
Supplement: Supplementary file 1 — Facilitator Guide - ECMO and ACD.docxLearning Objectives - ECMO and ACD.docxModule Presentation Slides - ECMO and ACD.pptxModule Presentation Recording - ECMO and ACD.mp4Module Quiz - ECMO and ACD.docxModule Quiz Answers - ECMO and ACD.docxPostmodule Survey Likert Questions.docx [file mep_2374-8265.11556-s001.zip › A. Facilitator Guide - ECMO and ACD.docx]

**Facilitator’s Guide for the Extracorporeal Membrane Oxygenation and Cardiovascular Devices Module**

Appendix A: Facilitator’s guide for the flipped-classroom and case-based module on **Extracorporeal Membrane Oxygenation and Cardiovascular Devices**

**Purpose & Goals**

The primary goal of this session is to increase learner familiarity, confidence, and clinical reasoning when managing advanced cardiovascular support devices, specifically:

- Extracorporeal Membrane Oxygenation (ECMO)
- Intra-aortic Balloon Pumps (IABPs)
- Other advanced cardiovascular technologies not commonly covered in traditional medical school curricula, such as Resuscitative Endovascular Balloon Occlusion of the Aorta (REBOA).

The flipped-classroom format is designed to:

- Encourage active learning and application of pre-recorded content.
- Foster critical thinking and discussion through case-based learning.
- Create a collaborative environment where learners support and challenge each other.

**Before the Session**

*Facilitator Checklist: Pre-Session Tasks*

☐ Distribute materials:

- Send out the pre-recorded lecture link and online quiz (5 questions) one week prior.
- Email the case study document below without the explanation.

☐ Instruct students to:

- Complete the pre-recorded lecture and quiz before the live session.
- Score ≥80% on the quiz (remediation to follow if needed).

☐ Review student quiz performance:

- Identify commonly missed questions for targeted review.

☐ Confirm platform setup:

- Schedule the session on a video-conferencing platform (Zoom, Teams, etc.).
- Enable breakout room functionality for small-group discussions.
- Ensure facilitators have co-host privileges.

☐ Invite an EM-Critical Care (EM-CCM) physician, if available, to enhance discussion with expert input.

**Session Agenda (60 Minutes Total)**

| **Segment** | **Duration** | **Facilitator Actions** |
| --- | --- | --- |
| Welcome & Review of Difficult Concepts | 5–10 min | Introduce faculty. Clarify ECMO physiology & IABP waveforms. Use slides or visuals if helpful. |
| Quiz Review & Group Debrief | 10 min | Go over 1–2 quiz questions students struggled with. Explain why correct answers are correct and why incorrect ones are not. Facilitate brief group discussion after each. |
| Small Group Case Discussions | 25–30 min | Divide learners into groups of 7–8 using breakout rooms. Each group is given the case study (provided below). Assign facilitators to each room if possible. |
| Regroup & Case Debrief | 10–15 min | Return to the main room. Have each group present their responses and rationale. Use a rotating format to ensure equal participation. |
| Open Q&A / Wrap-up | 5 min | Call on each student (not just group-wide) for questions or reflections. Offer take-home points and additional reading if desired. |

**Facilitation Tips & Best Practices**

*Make It Engaging & Focused*

- Encourage students to reference the pre-recorded lecture or notes during the session.
- Remind groups to stay focused on the case—unrelated tangents can derail learning.
- Phrase questions to stimulate deeper thinking, especially during follow-up questions.
- If possible, rotate facilitators between breakout rooms to balance expertise.

*During Small Group Discussions*

- Use the case provided below.
- Pose at least three questions:
  - Start with the multiple-choice question provided.
  - Follow up with 1–2 open-ended questions for application or ethical considerations.
- Assign a group leader or rotate who shares out.

*Leverage Experts*

- If an EM-CCM physician is present, invite them to provide “educational pearls” or highlight real-world application.
- If learners are shy, use the chat or call on them by name to ensure participation.

**Case Study for Discussion**

*Clinical Vignette:*A patient presents with severe influenza after returning from vacation. They progress to sepsis and septic shock. EMS intubates the patient for respiratory failure. In the ED, an X-ray shows signs concerning for ARDS. The patient is admitted to the ICU, remains on 100% FiO₂, with PaO₂ persistently low at 30–32 mmHg. WBC count improves slightly (25K → 19K), but the patient remains acidotic (ABG shows pH 7.02). Further attempts at stabilization, including hemodynamic resuscitation with vasopressors, ventilator adjustment strategies, proning, and paralysis are unsuccessful.

*Prompt to Learners:*What could you do to optimize care for this patient at this point?

*Multiple Choice Options:*A) Mechanical ventilator support with lung-protective settings and diuresis
B) VA-ECMO
C) VV-ECMO
D) REBOA placement
E) Discontinue, inform the family the patient cannot be weaned

**Case Explanation for Facilitators**

Correct Answer: C) VV-ECMO

- The patient is experiencing refractory hypoxemia and metabolic acidosis despite appropriate ventilatory strategies. This is a classic scenario for VV-ECMO consideration.
- RESP score (Respiratory ECMO Survival Prediction) can be introduced as a clinical decision-making tool.
- Emphasize the incorrect answers:
  - Answer A: Mechanical ventilator support with lung-protective settings and diuresis is not appropriate due to the patients failing condition, despite adequate ventilation strategies, which indication the need for VV-ECMO in this case. Lung-protective strategies in ARDS, which target a low-volume/high-PEEP approach, can be considered; however, given this patient’s rapidly deteriorating situation, VV-ECMO should be considered early to address the developing metabolic acidosis and critically low PaO2. Diuresis would also be inappropriate in this situation, as the hypoxemia was not from a volume overload state and due to the gravity of the patient’s condition.
  - Answer B: VA-ECMO is not appropriate in this scenario because there was no mention of concomitant cardiac compromise, and this was likely a primary respiratory failure.
  - Answer D: REBOA placement is not appropriate in this scenario, as this patient had no signs or suspicion of trauma or life-threatening hemorrhage. Instead, this patient likely had septic shock as a result of influenza, which led to ARDS and ultimately respiratory failure. Indications for REBOA include traumatic or non-traumatic life-threatening hemorrhage below the diaphragm in patients experiencing hemorrhagic shock who are unresponsive.
  - Answer E: Withdrawal of care is incorrect, as this patient still meets the criteria for VV-ECMO. Unless the patient has a pre-existing DNR status or the family reaches a decision on DNR status because patient is too critically-ill to make decisions for themselves, measures involving VV-ECMO should be considered for a potentially reversible condition.

Follow-up Questions (for deeper discussion)

1. What criteria must be met before initiating VV-ECMO?
2. How would the management differ if this were a combined cardiopulmonary failure?
3. What are the ethical and resource-based considerations when deciding on ECMO?

**Post-Session Tasks for Facilitators**

☐ Encourage students to complete the post-module quiz and feedback survey.

☐ Use responses to evaluate effectiveness and areas for improvement.

☐ Debrief with other facilitators: What worked? What didn’t? Any student feedback?

☐ Consider updating content or pacing for future sessions based on engagement and outcomes.

**Final Tips for Success**

- Keep things interactive: Ask “why” often.
- Use real-life analogies when explaining complex physiology.
- Be patient with silence, it often means students are thinking.
- Make space for curiosity, allow follow-up questions even if off-script.
