# Supplementary material for: Effectiveness of the Essential Critical Care Concepts in Emergency Medicine: Extracorporeal Membrane Oxygenation and Cardiovascular Devices Module Implementation
Source: MedEdPORTAL. 2025 Nov 7;21:11556. doi: 10.15766/mep_2374-8265.11556 (PMC12592219; doi:10.15766/mep_2374-8265.11556)
Supplement: Supplementary file 1 — Facilitator Guide - ECMO and ACD.docxLearning Objectives - ECMO and ACD.docxModule Presentation Slides - ECMO and ACD.pptxModule Presentation Recording - ECMO and ACD.mp4Module Quiz - ECMO and ACD.docxModule Quiz Answers - ECMO and ACD.docxPostmodule Survey Likert Questions.docx [file mep_2374-8265.11556-s001.zip › B. Learning Objectives - ECMO and ACD.docx]

**Extracorporeal Membrane Oxygenation and Cardiovascular Devices Module Learning Objectives**

1.1 - Describe the goals, procedure, clinical uses and contraindications of IABP therapy

1.2 - Demonstrate the ability to understand an IABP waveform and potential timing errors

1.3 - Describe the two forms of ECMO therapy along with their indications and contraindications

1.4 - Describe the physiology and mechanics behind ECMO therapy

1.5 - Describe the goals and procedure of REBOA therapy
