# Supplementary material for: Effectiveness of the Essential Critical Care Concepts in Emergency Medicine: Extracorporeal Membrane Oxygenation and Cardiovascular Devices Module Implementation
Source: MedEdPORTAL. 2025 Nov 7;21:11556. doi: 10.15766/mep_2374-8265.11556 (PMC12592219; doi:10.15766/mep_2374-8265.11556)
Supplement: Supplementary file 1 — Facilitator Guide - ECMO and ACD.docxLearning Objectives - ECMO and ACD.docxModule Presentation Slides - ECMO and ACD.pptxModule Presentation Recording - ECMO and ACD.mp4Module Quiz - ECMO and ACD.docxModule Quiz Answers - ECMO and ACD.docxPostmodule Survey Likert Questions.docx [file mep_2374-8265.11556-s001.zip › C. Module Presentation Slides - ECMO and ACD.pptx]

## Slide 1
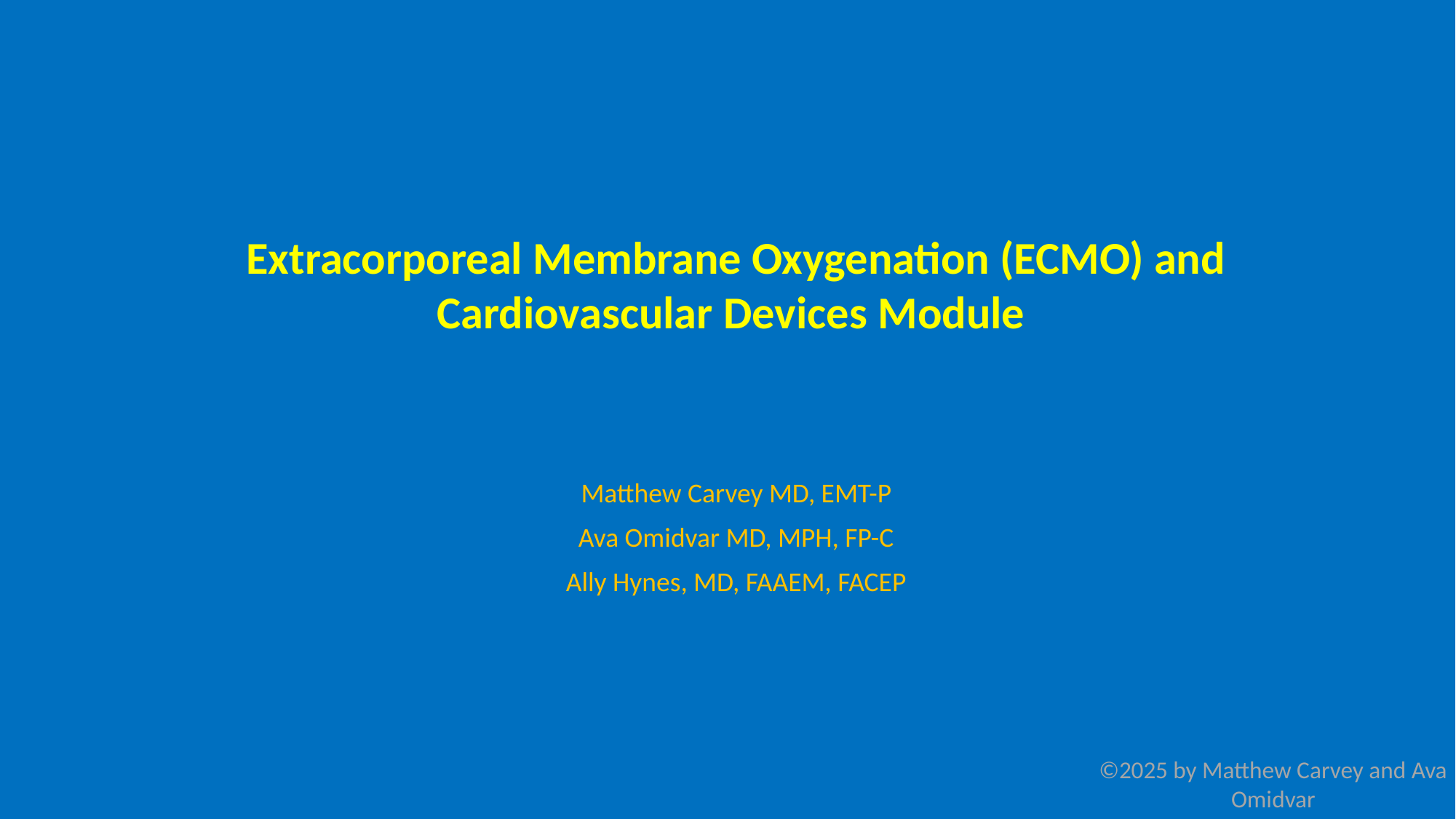

Extracorporeal Membrane Oxygenation (ECMO) and Cardiovascular Devices Module
Matthew Carvey MD, EMT-P
Ava Omidvar MD, MPH, FP-C
Ally Hynes, MD, FAAEM, FACEP
©2025 by Matthew Carvey and Ava Omidvar

## Slide 2
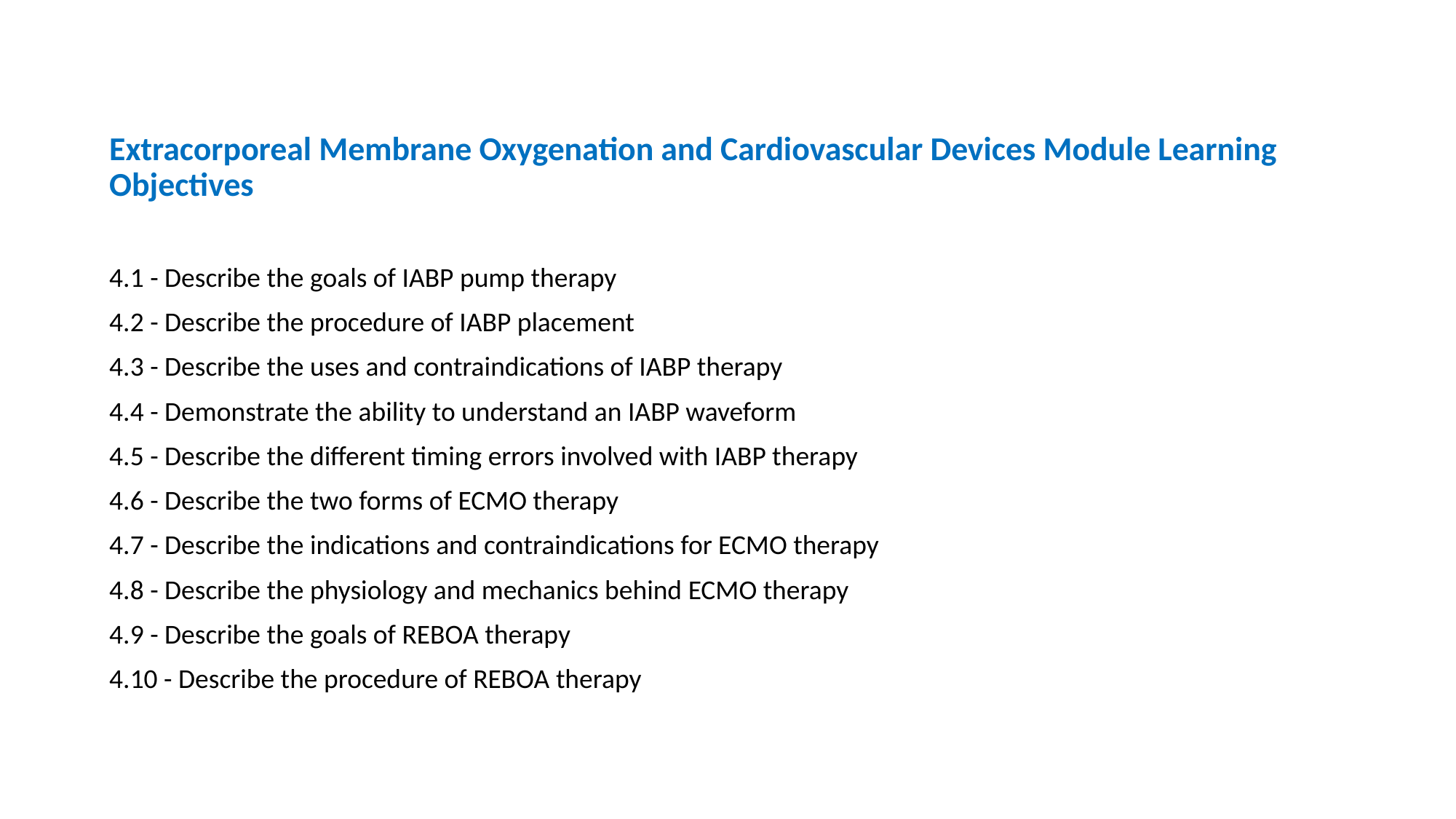

Extracorporeal Membrane Oxygenation and Cardiovascular Devices Module Learning Objectives
4.1 - Describe the goals of IABP pump therapy
4.2 - Describe the procedure of IABP placement
4.3 - Describe the uses and contraindications of IABP therapy
4.4 - Demonstrate the ability to understand an IABP waveform
4.5 - Describe the different timing errors involved with IABP therapy
4.6 - Describe the two forms of ECMO therapy
4.7 - Describe the indications and contraindications for ECMO therapy
4.8 - Describe the physiology and mechanics behind ECMO therapy
4.9 - Describe the goals of REBOA therapy
4.10 - Describe the procedure of REBOA therapy

## Slide 3
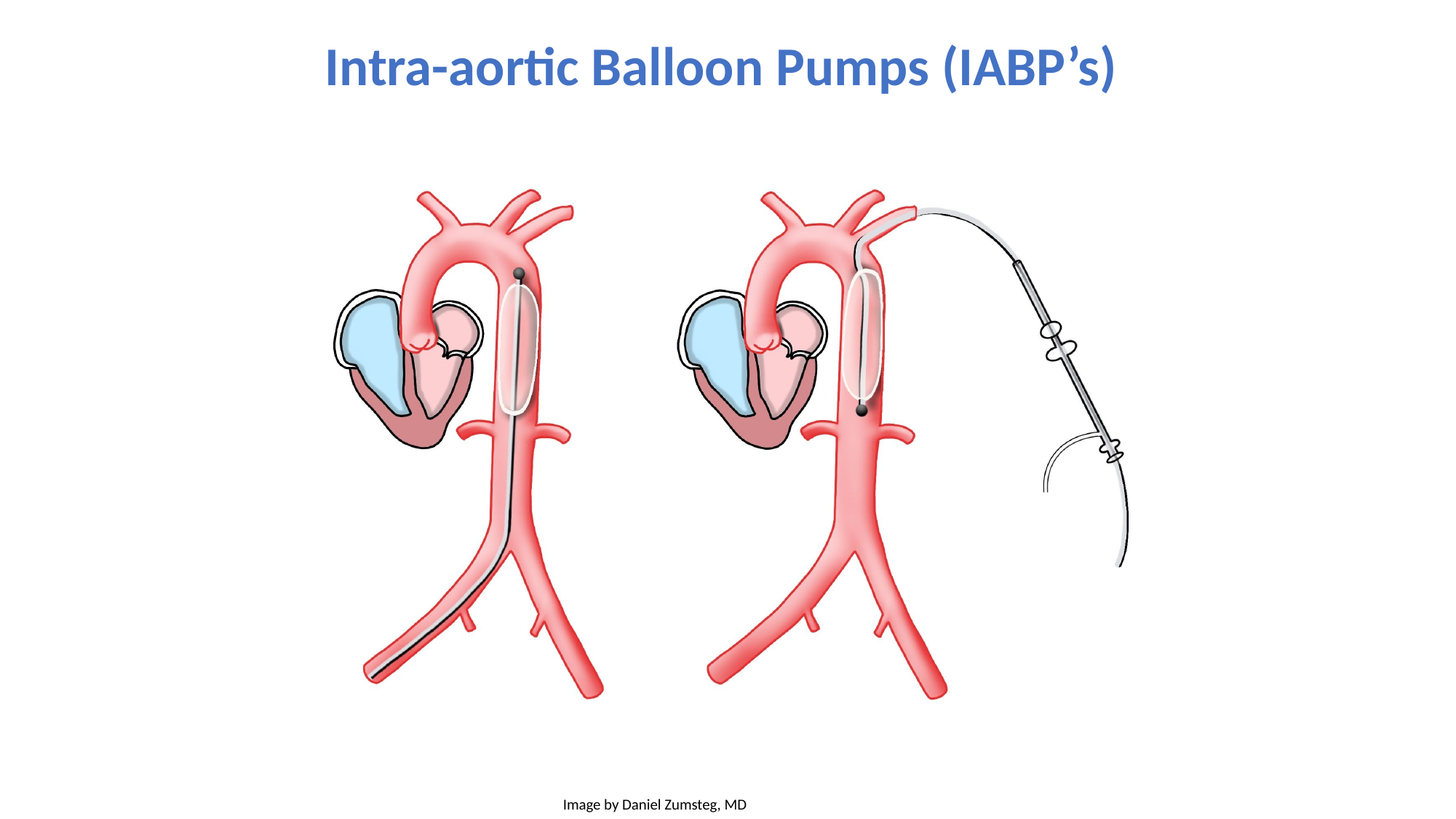

Intra-aortic Balloon Pumps (IABP’s)
Image by Daniel Zumsteg, MD

## Slide 4
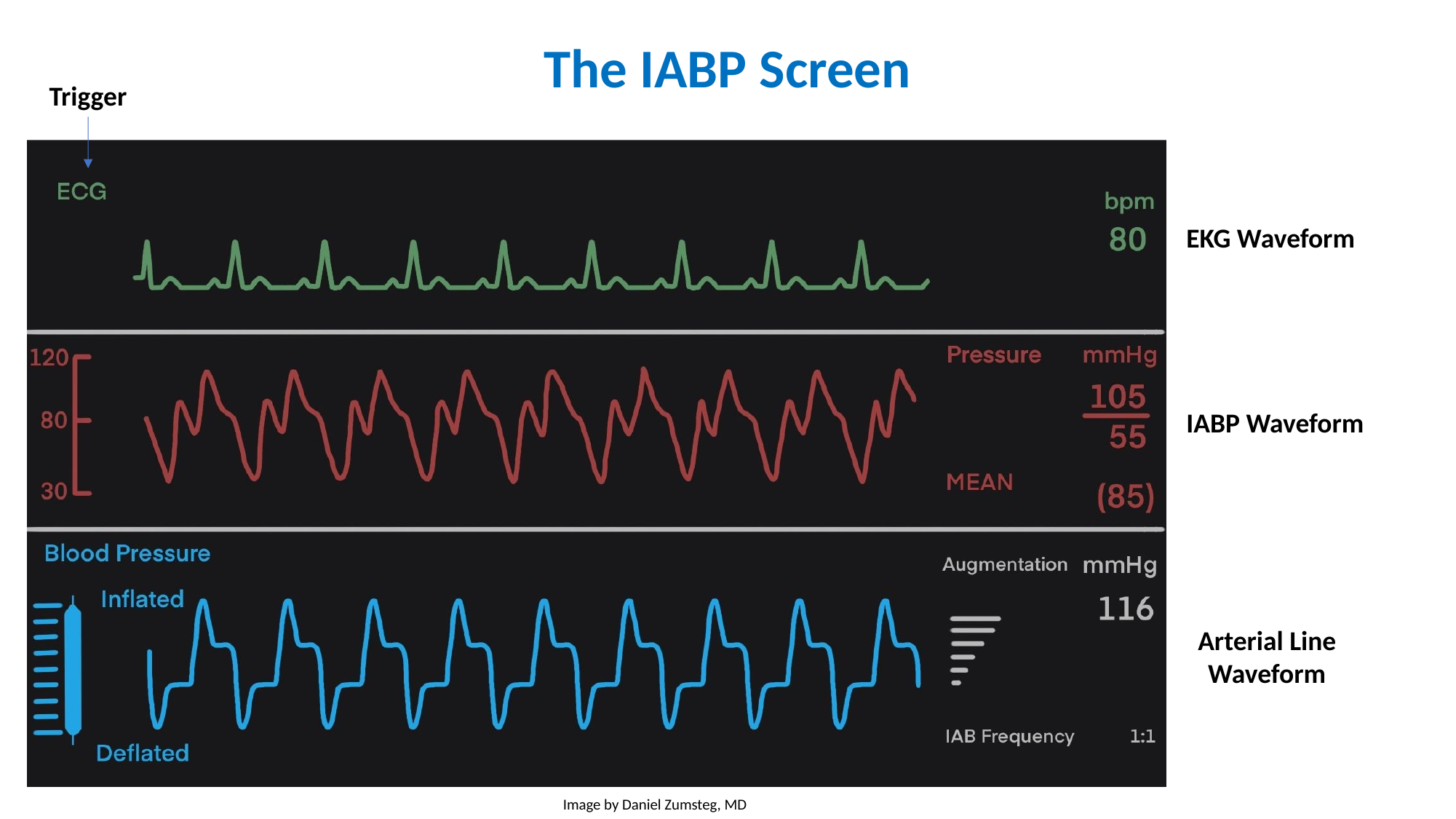

# The IABP Screen
Trigger
EKG Waveform
IABP Waveform
Arterial Line Waveform
Image by Daniel Zumsteg, MD

## Slide 5
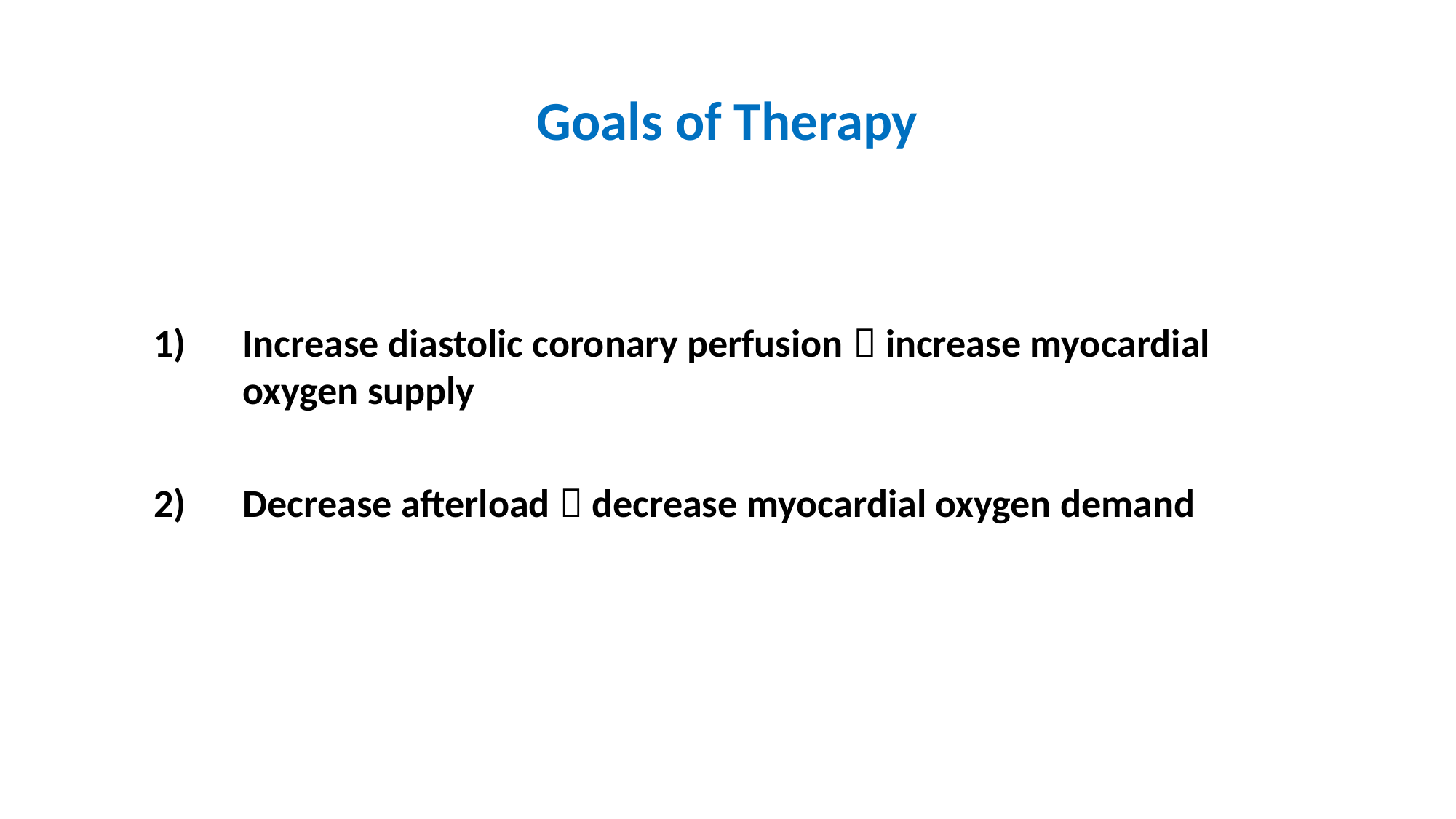

# Goals of Therapy
Increase diastolic coronary perfusion  increase myocardial oxygen supply
Decrease afterload  decrease myocardial oxygen demand

## Slide 6
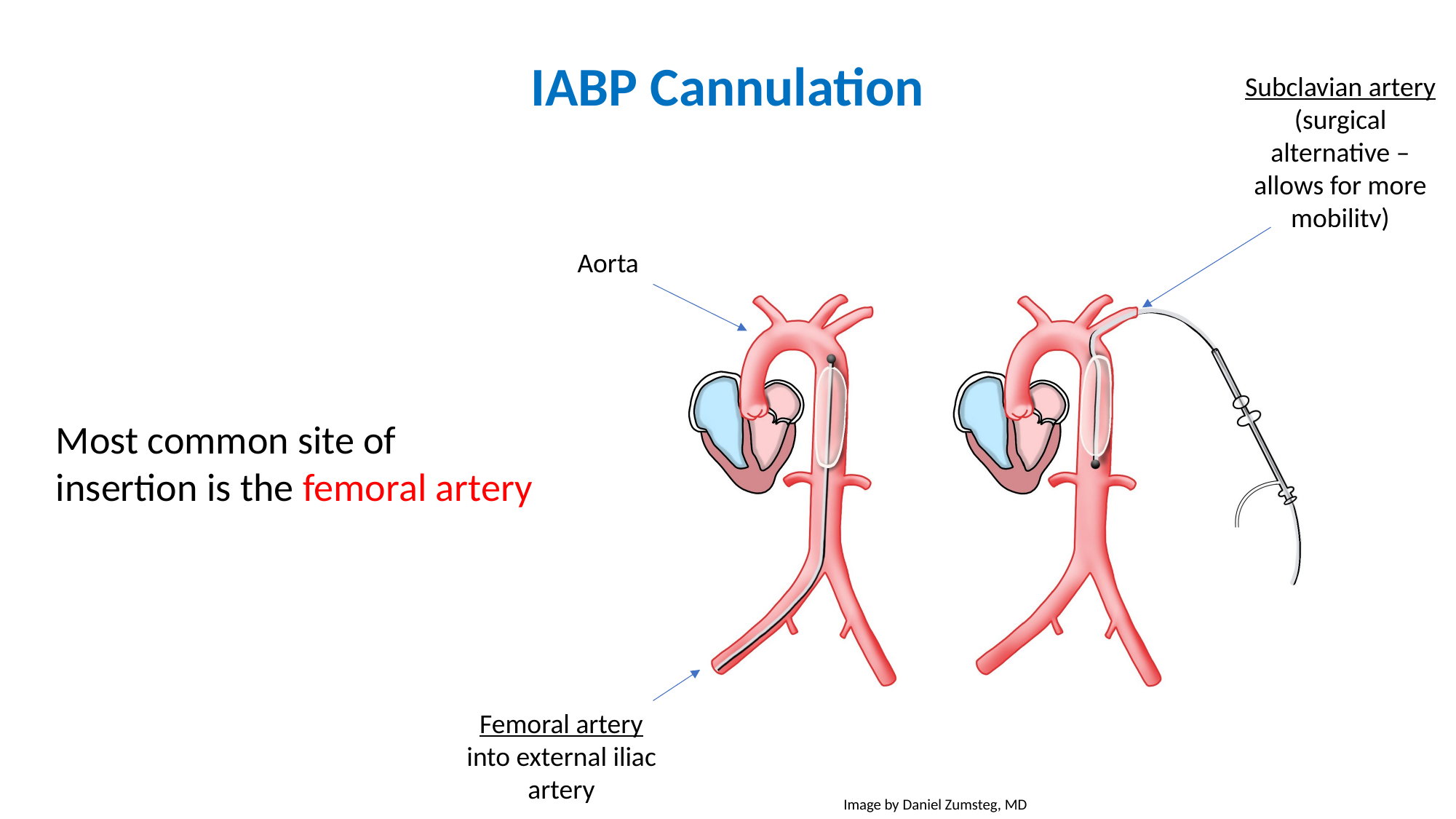

# IABP Cannulation
Subclavian artery (surgical alternative – allows for more mobility)
Aorta
Most common site of insertion is the femoral artery
Common iliac artery
Femoral artery into external iliac artery
Image by Daniel Zumsteg, MD

## Slide 7
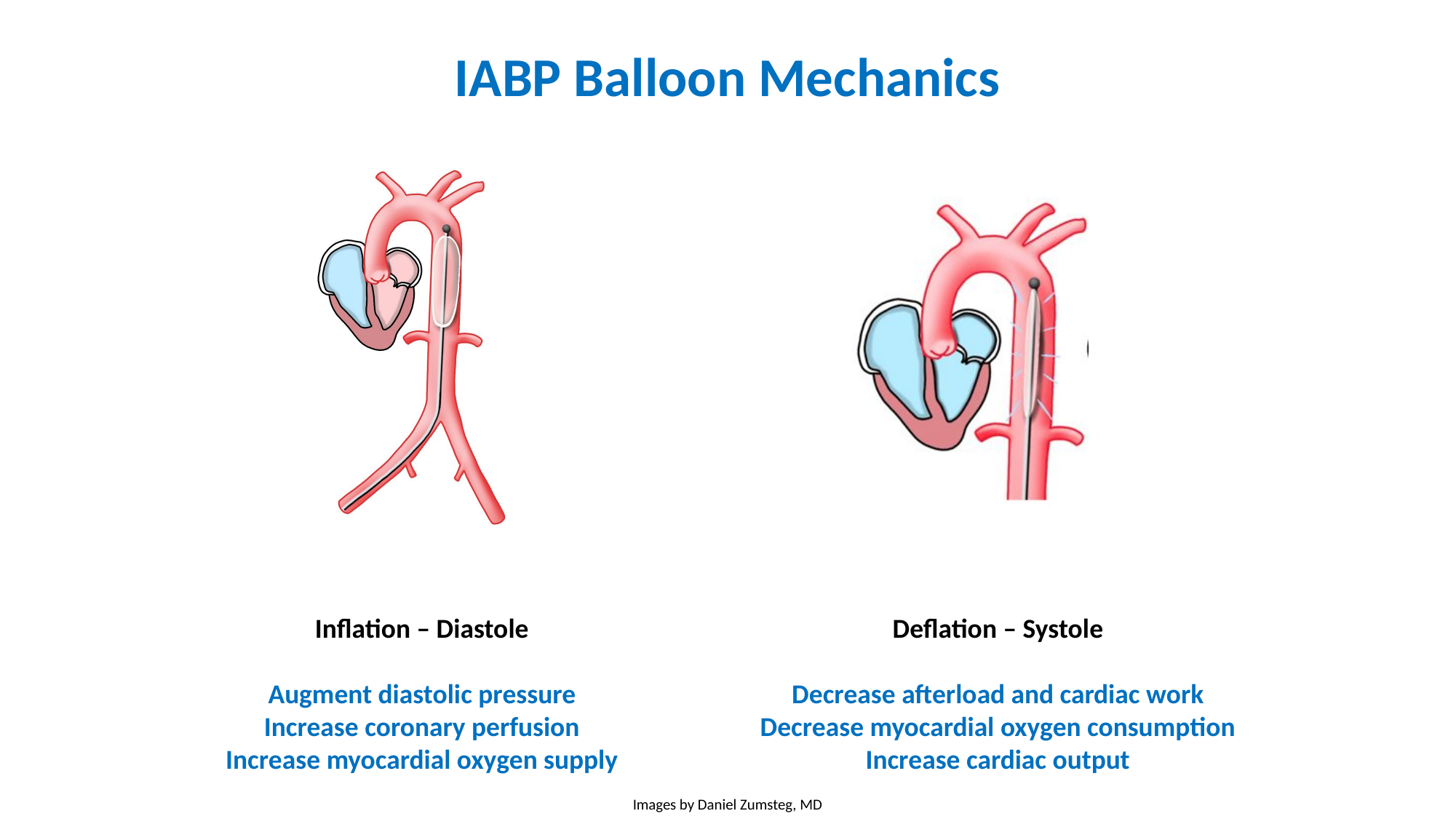

# IABP Balloon Mechanics
Inflation – Diastole
Augment diastolic pressure
Increase coronary perfusion
Increase myocardial oxygen supply
Deflation – Systole
Decrease afterload and cardiac work
Decrease myocardial oxygen consumption
Increase cardiac output
Images by Daniel Zumsteg, MD

## Slide 8
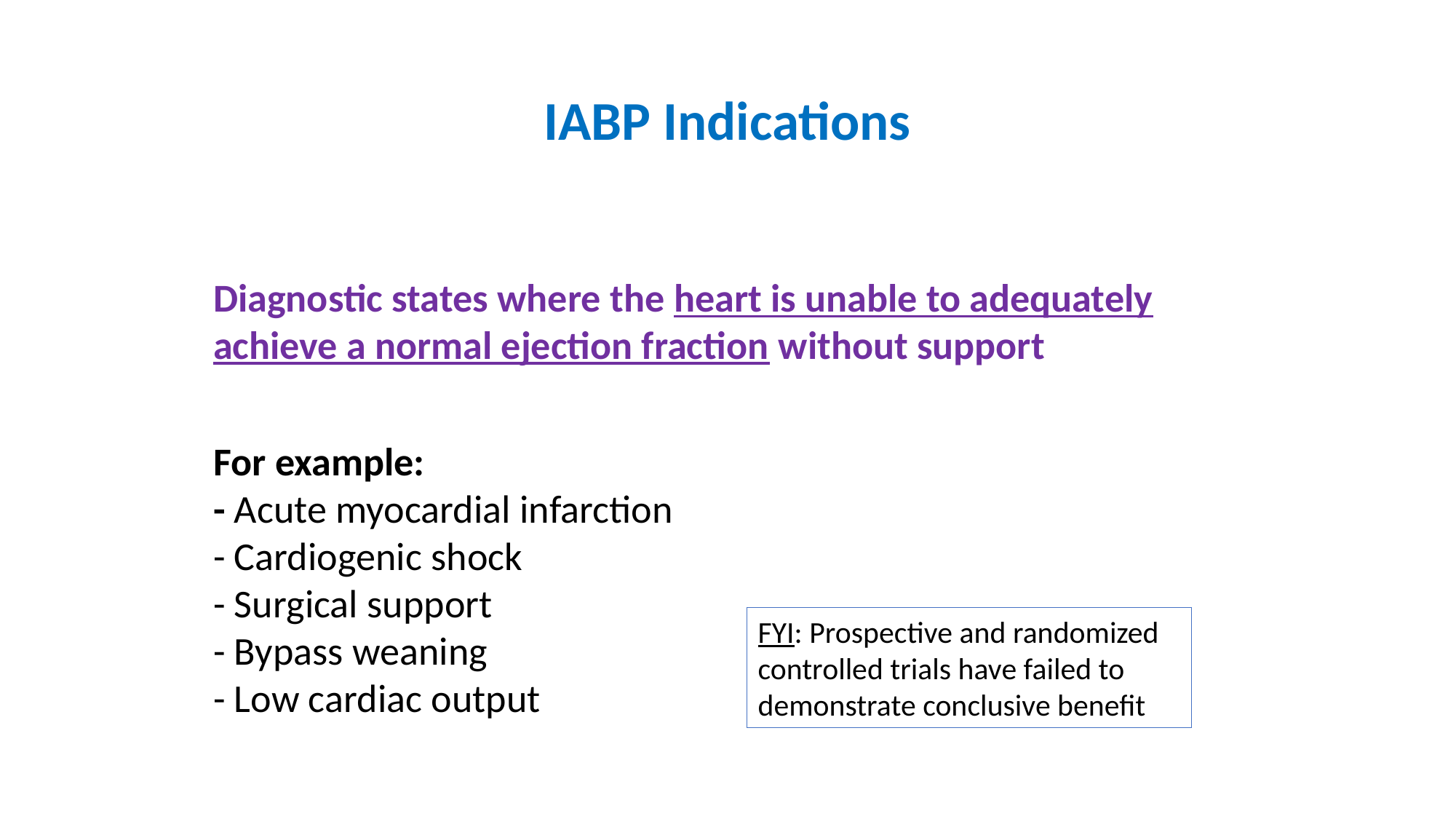

# IABP Indications
Diagnostic states where the heart is unable to adequately achieve a normal ejection fraction without support
For example:
- Acute myocardial infarction
- Cardiogenic shock
- Surgical support
- Bypass weaning
- Low cardiac output
FYI: Prospective and randomized controlled trials have failed to demonstrate conclusive benefit

## Slide 9
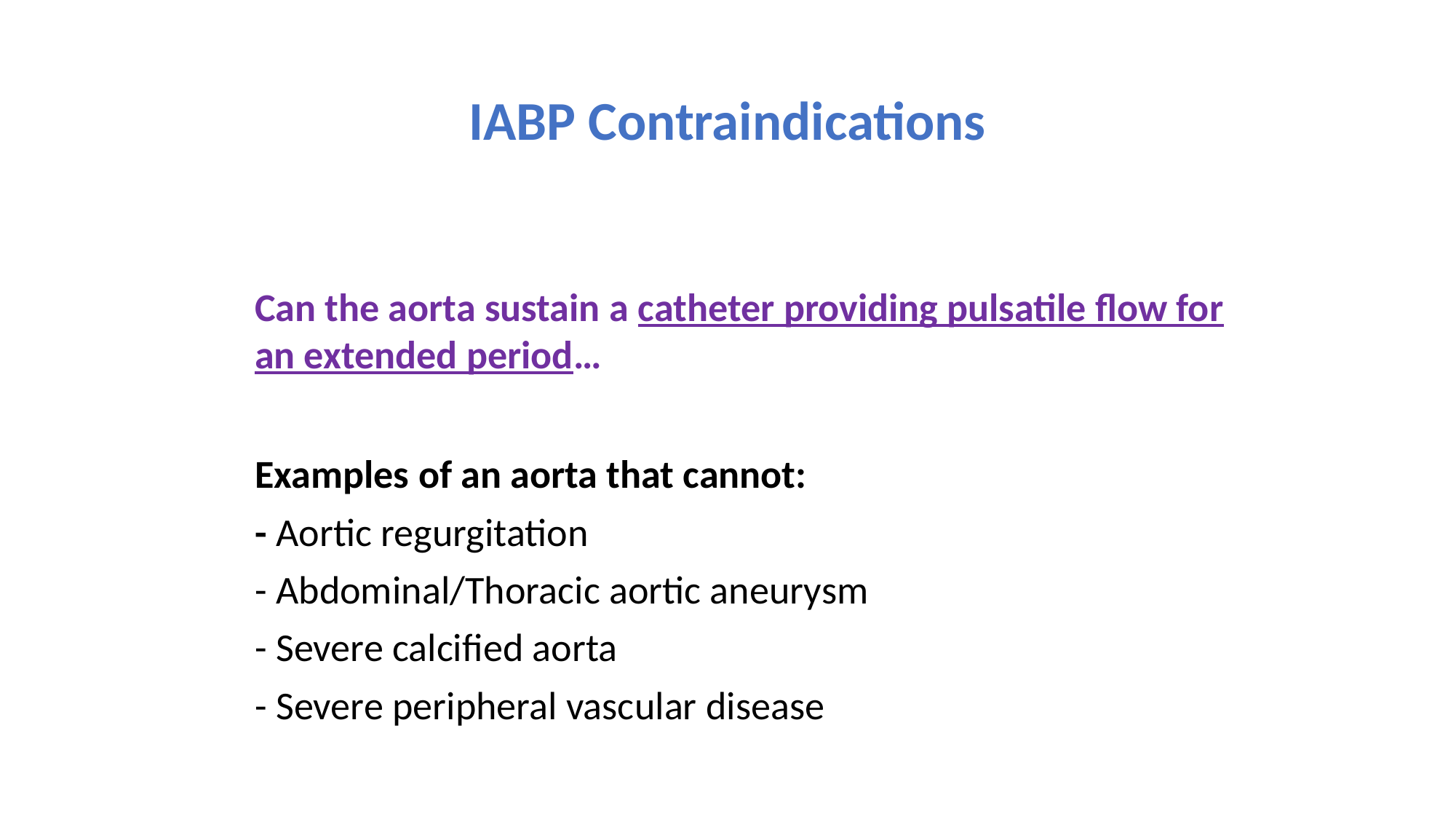

# IABP Contraindications
Can the aorta sustain a catheter providing pulsatile flow for an extended period…
Examples of an aorta that cannot:
- Aortic regurgitation
- Abdominal/Thoracic aortic aneurysm
- Severe calcified aorta
- Severe peripheral vascular disease

## Slide 10
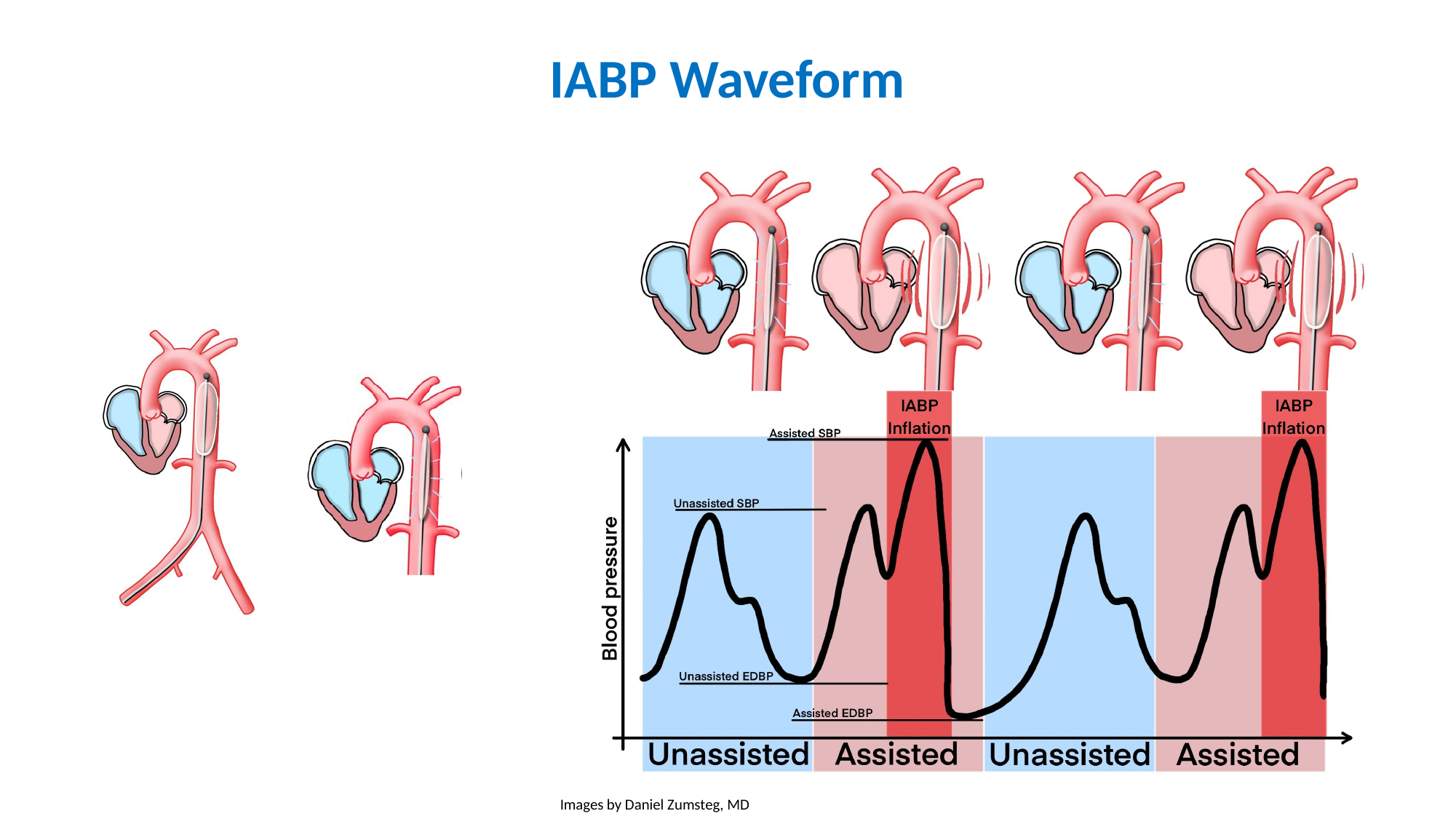

# IABP Waveform
Images by Daniel Zumsteg, MD

## Slide 11
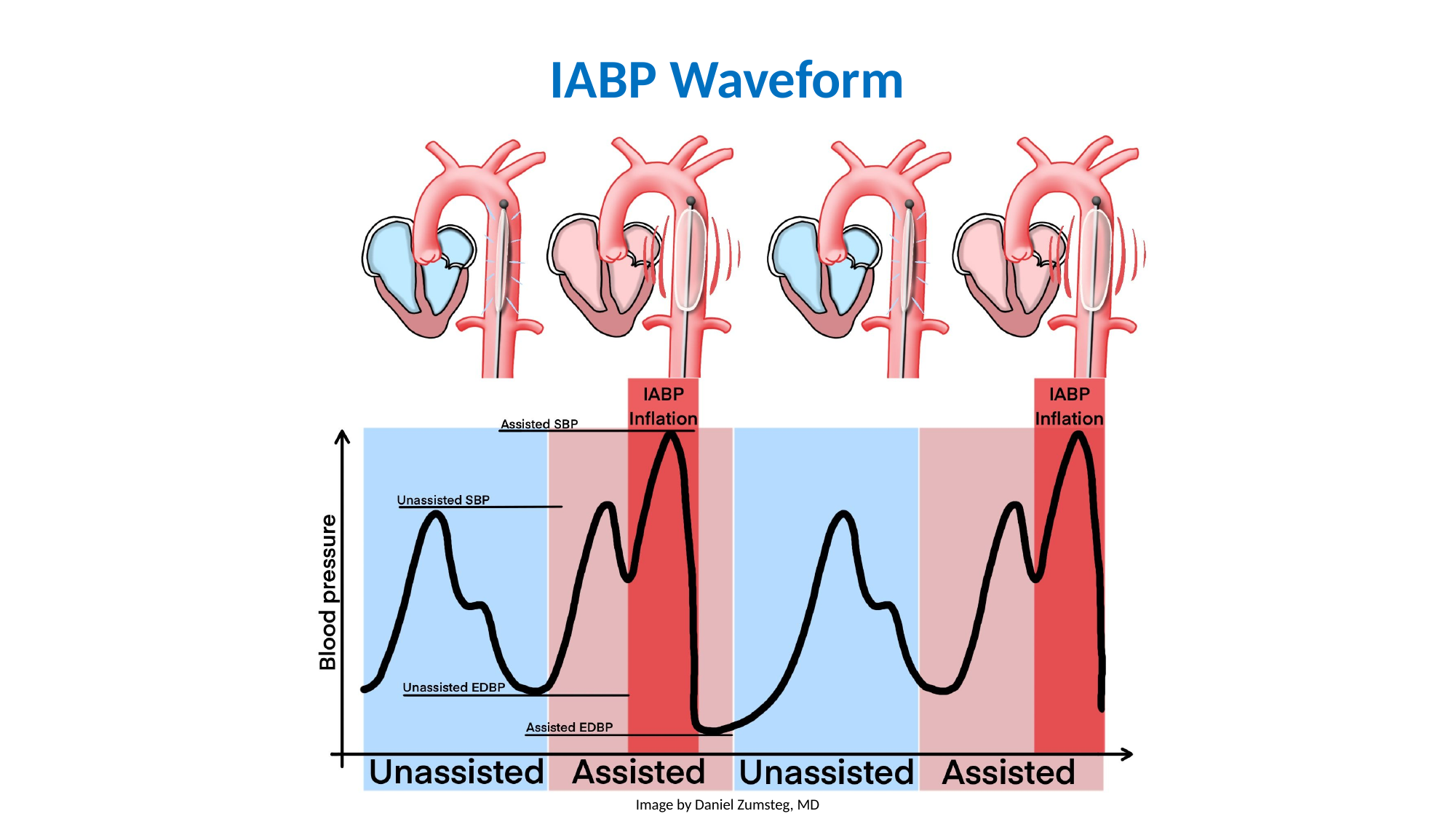

# IABP Waveform
Image by Daniel Zumsteg, MD

## Slide 12
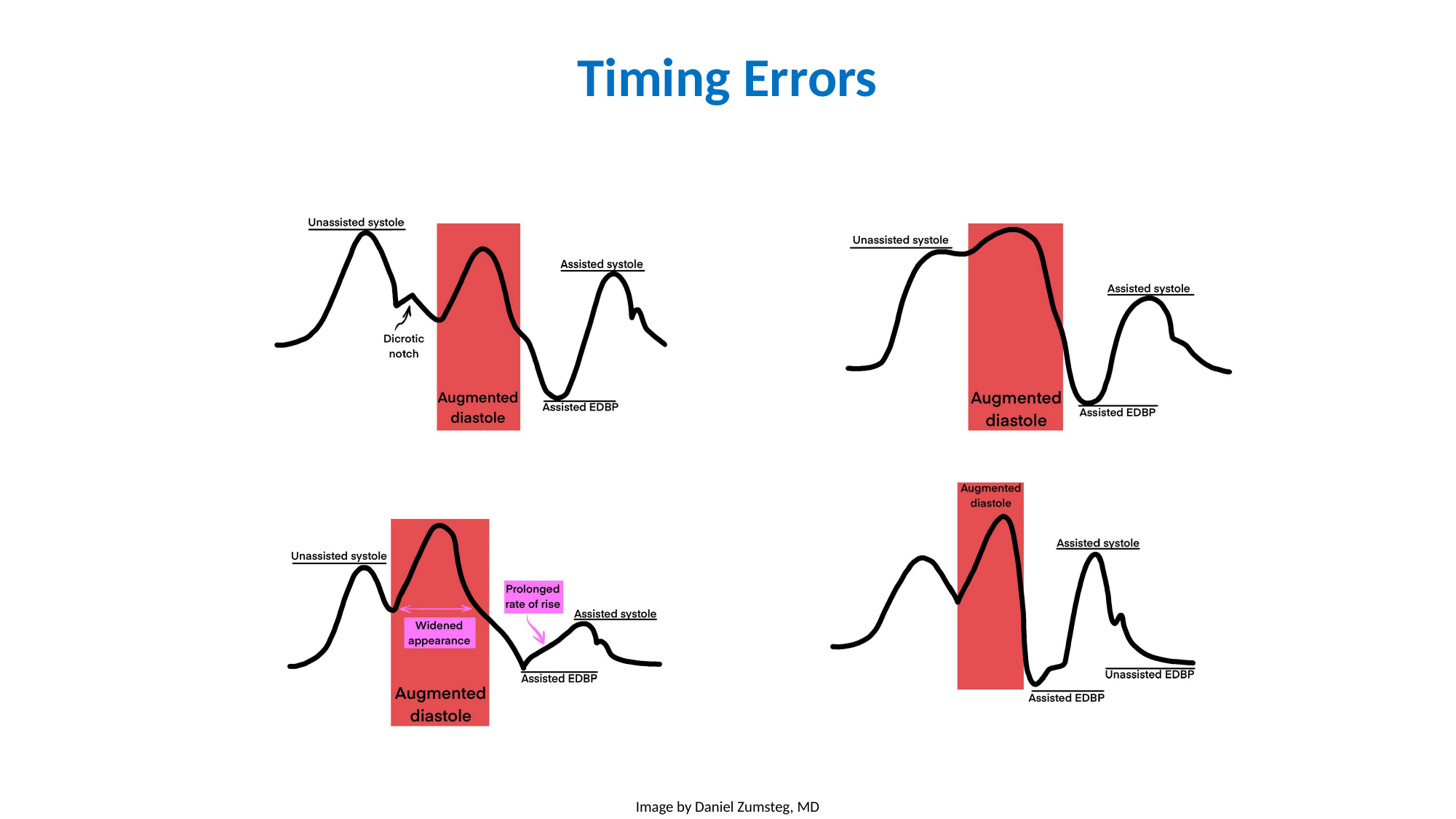

# Timing Errors
Image by Daniel Zumsteg, MD

## Slide 13
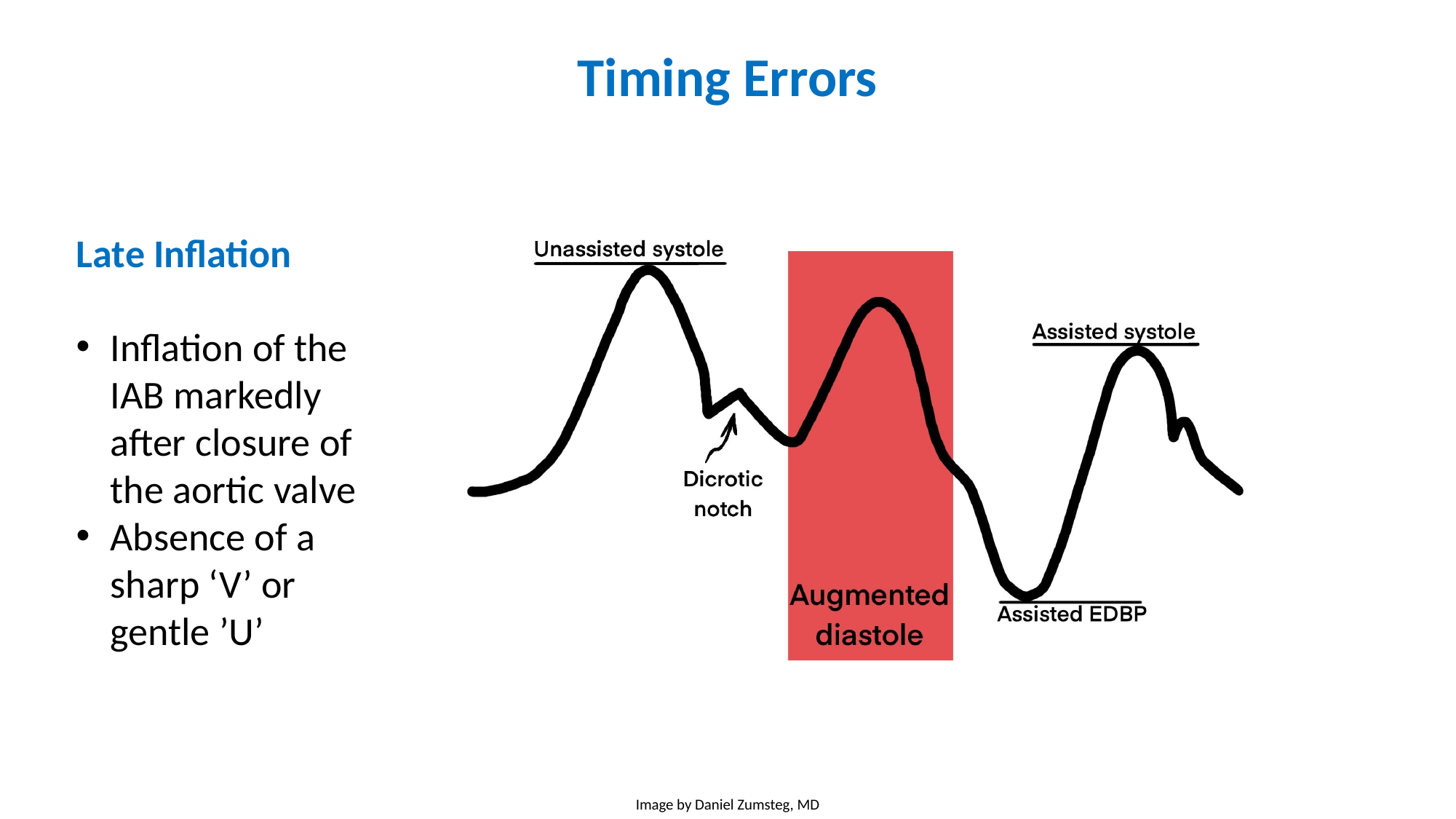

# Timing Errors
Late Inflation
Inflation of the IAB markedly after closure of the aortic valve
Absence of a sharp ‘V’ or gentle ’U’
Image by Daniel Zumsteg, MD

## Slide 14
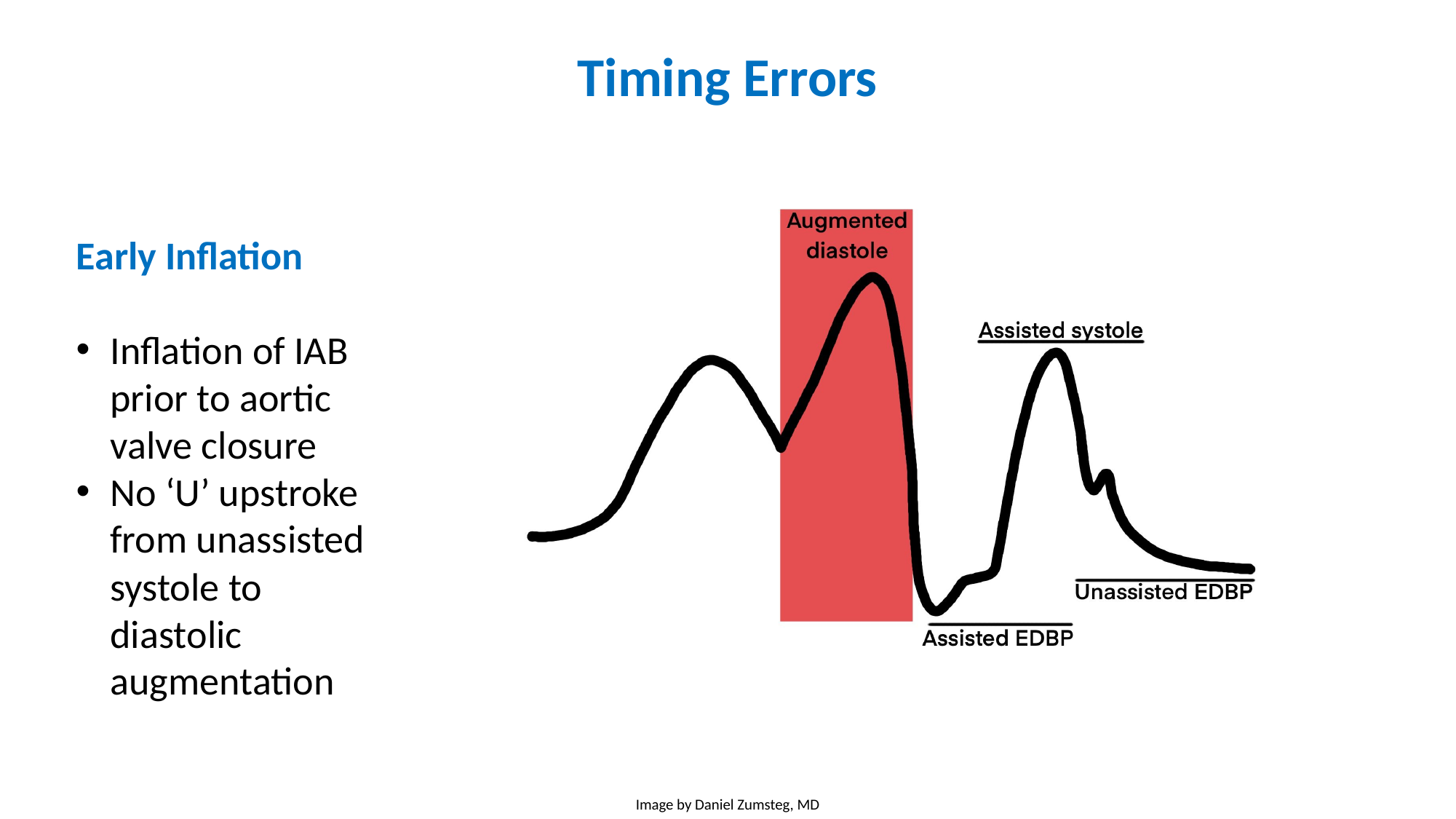

# Timing Errors
Early Inflation
Inflation of IAB prior to aortic valve closure
No ‘U’ upstroke from unassisted systole to diastolic augmentation
Image by Daniel Zumsteg, MD

## Slide 15
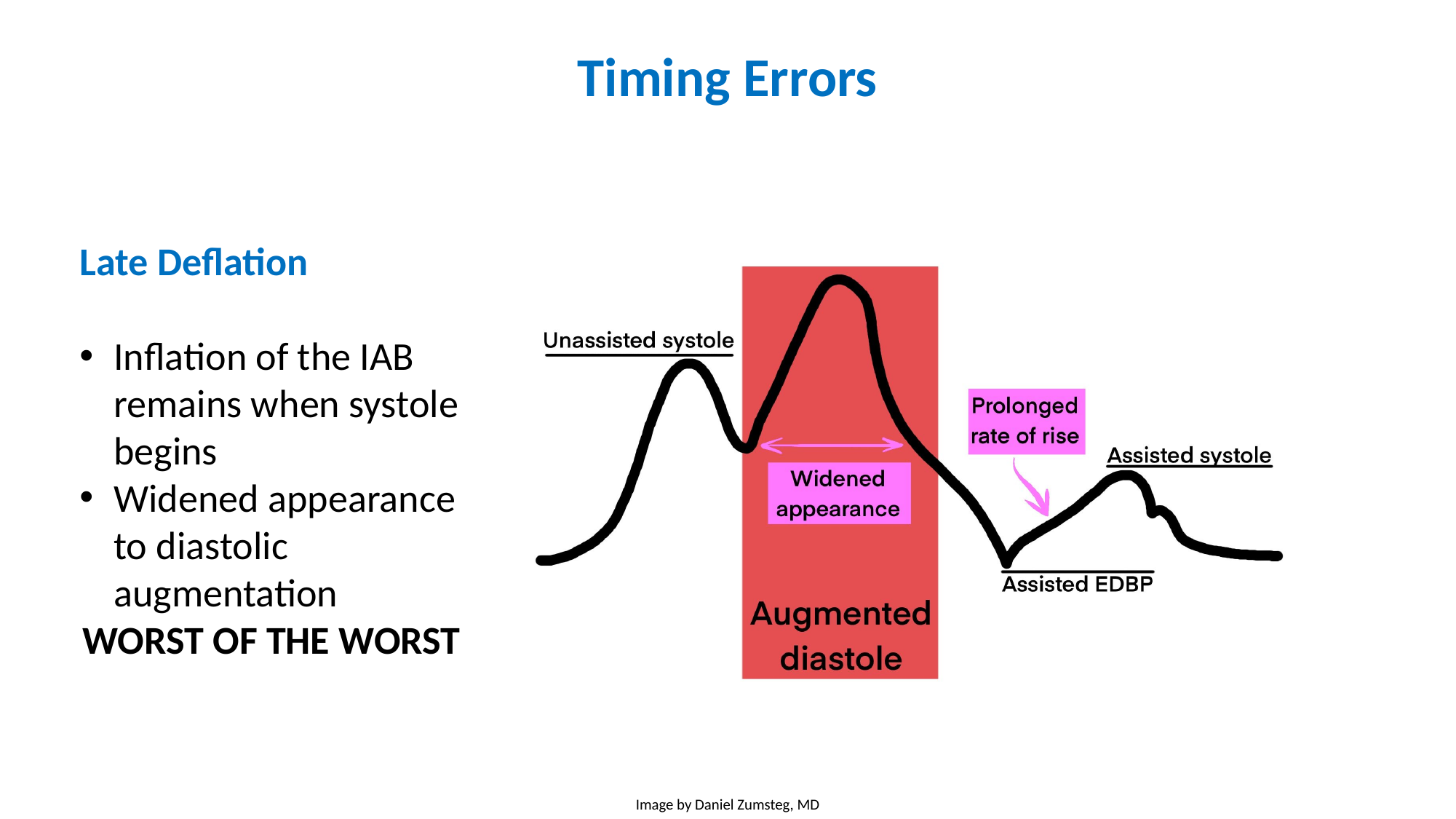

# Timing Errors
Late Deflation
Inflation of the IAB remains when systole begins
Widened appearance to diastolic augmentation
WORST OF THE WORST
Image by Daniel Zumsteg, MD

## Slide 16
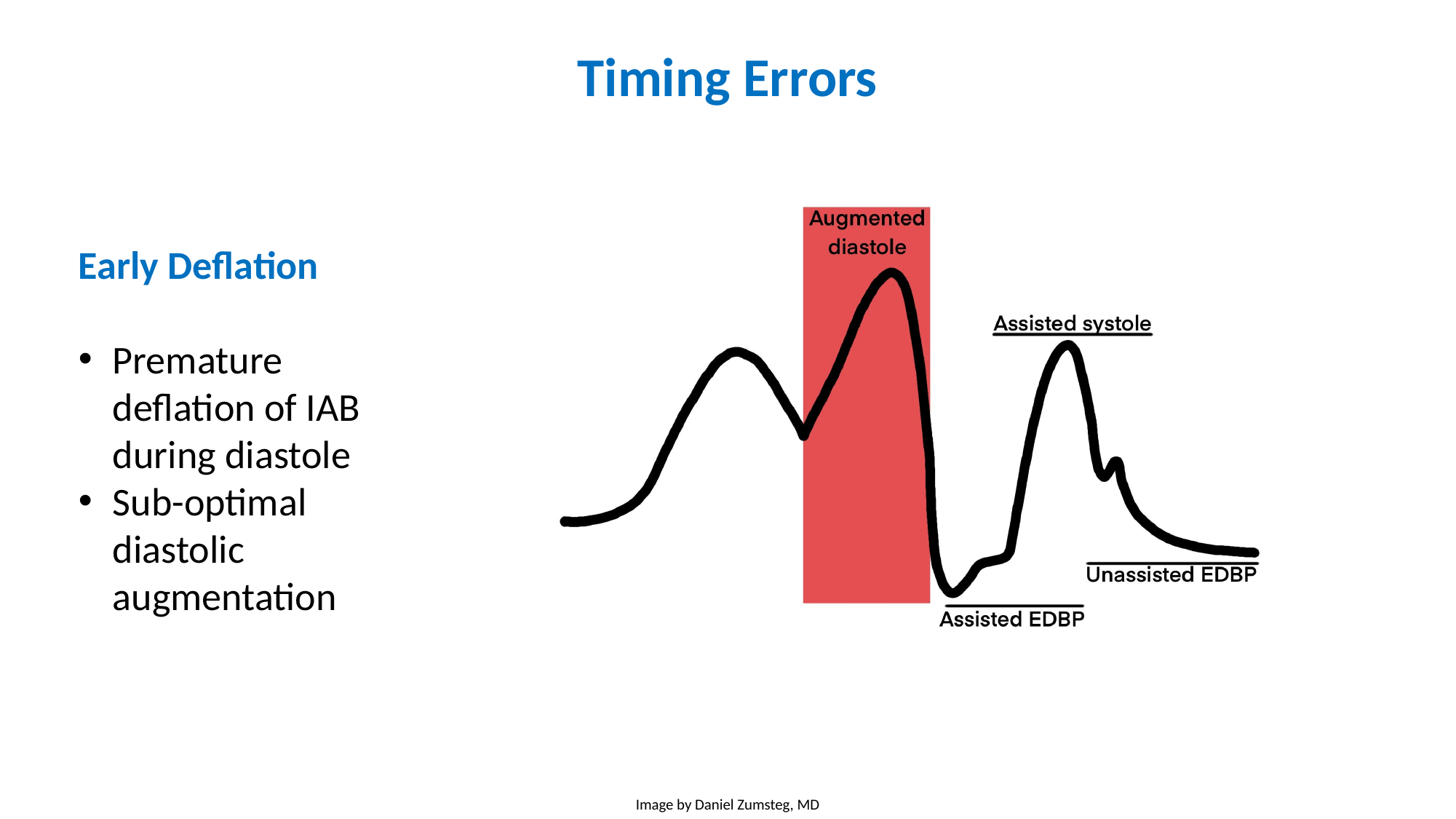

# Timing Errors
Early Deflation
Premature deflation of IAB during diastole
Sub-optimal diastolic augmentation
Image by Daniel Zumsteg, MD

## Slide 17
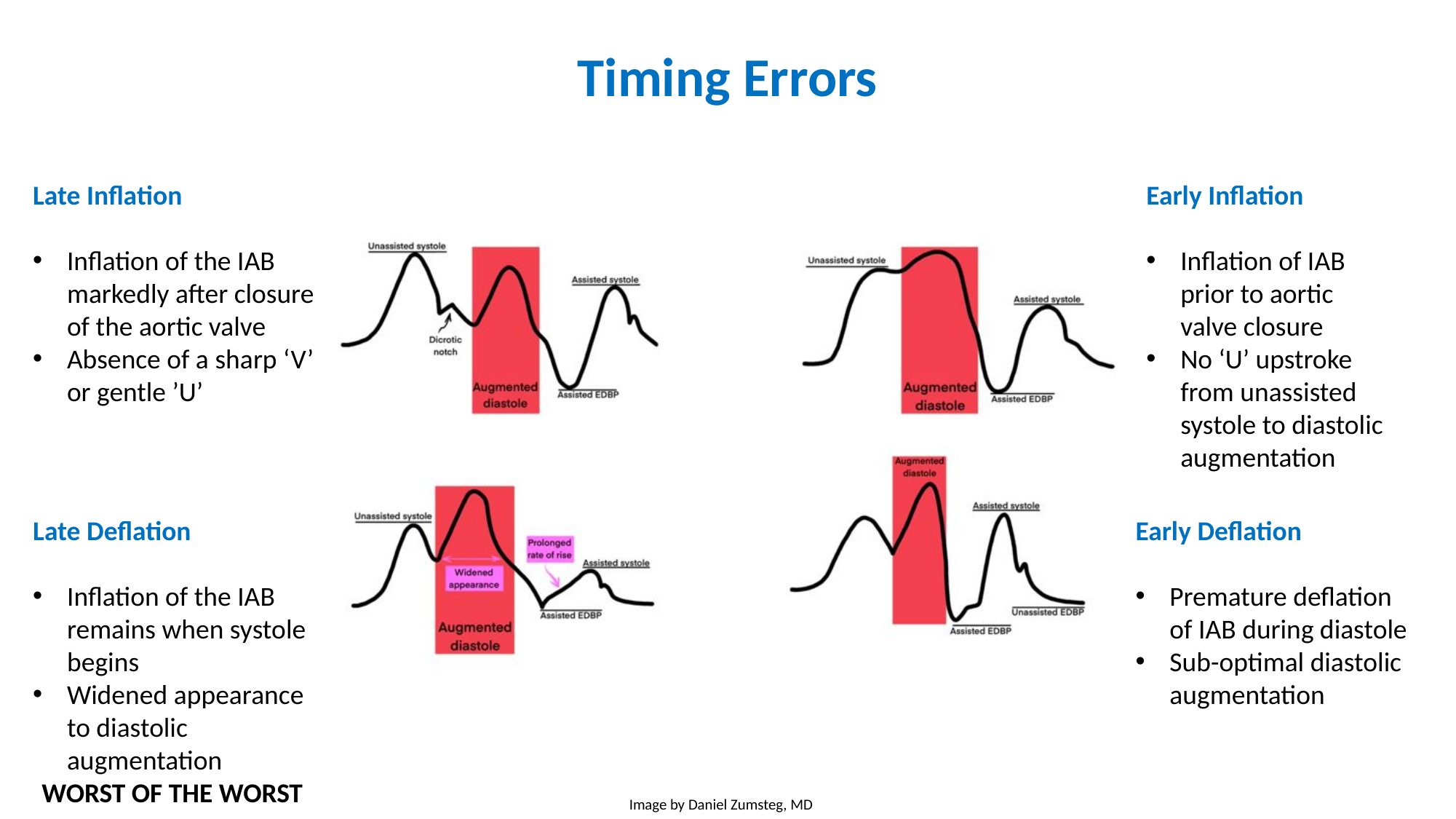

# Timing Errors
Late Inflation
Inflation of the IAB markedly after closure of the aortic valve
Absence of a sharp ‘V’ or gentle ’U’
Early Inflation
Inflation of IAB prior to aortic valve closure
No ‘U’ upstroke from unassisted systole to diastolic augmentation
Late Deflation
Inflation of the IAB remains when systole begins
Widened appearance to diastolic augmentation
WORST OF THE WORST
Early Deflation
Premature deflation of IAB during diastole
Sub-optimal diastolic augmentation
Image by Daniel Zumsteg, MD

## Slide 18
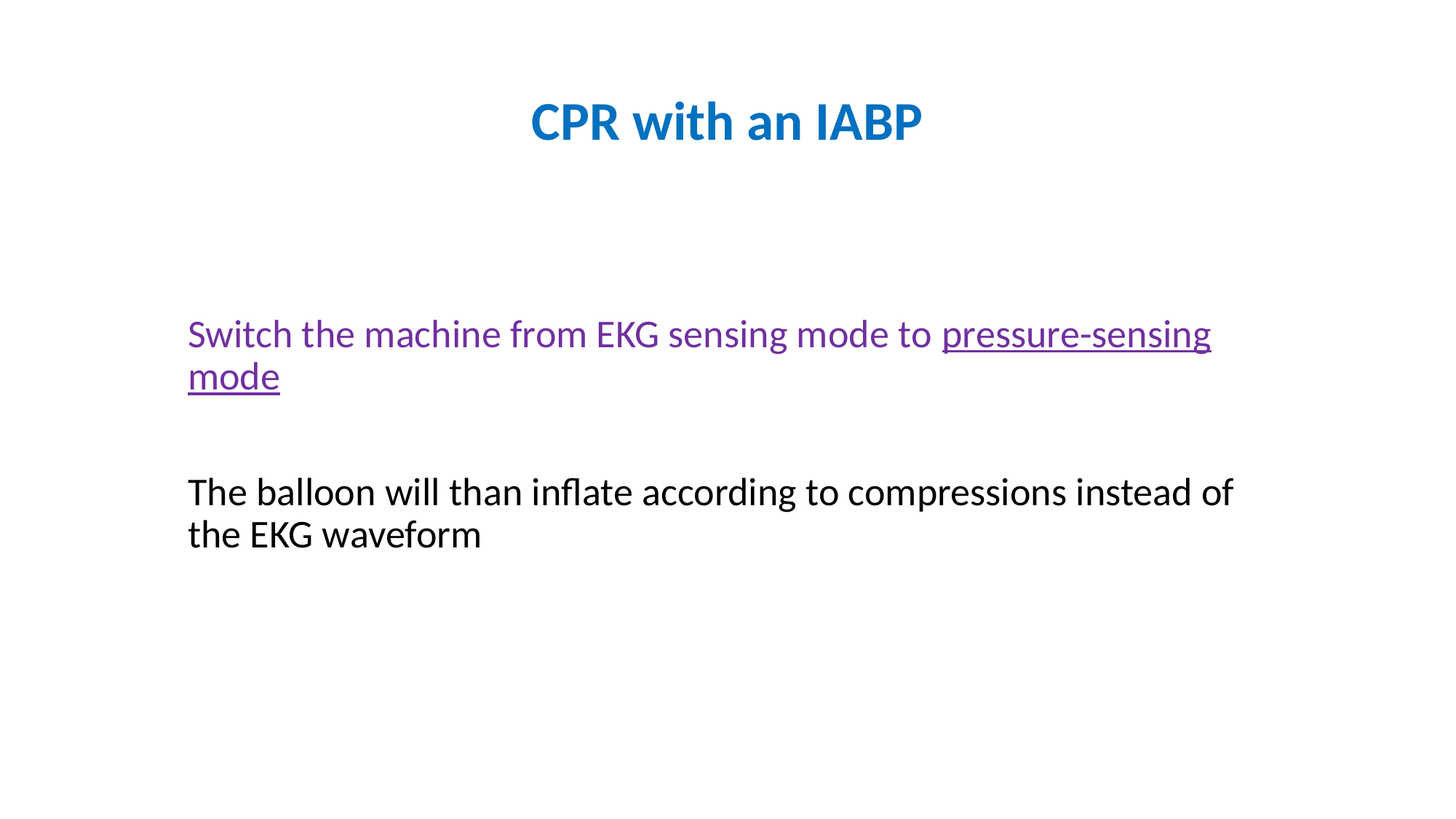

# CPR with an IABP
Switch the machine from EKG sensing mode to pressure-sensing mode
The balloon will than inflate according to compressions instead of the EKG waveform

## Slide 19
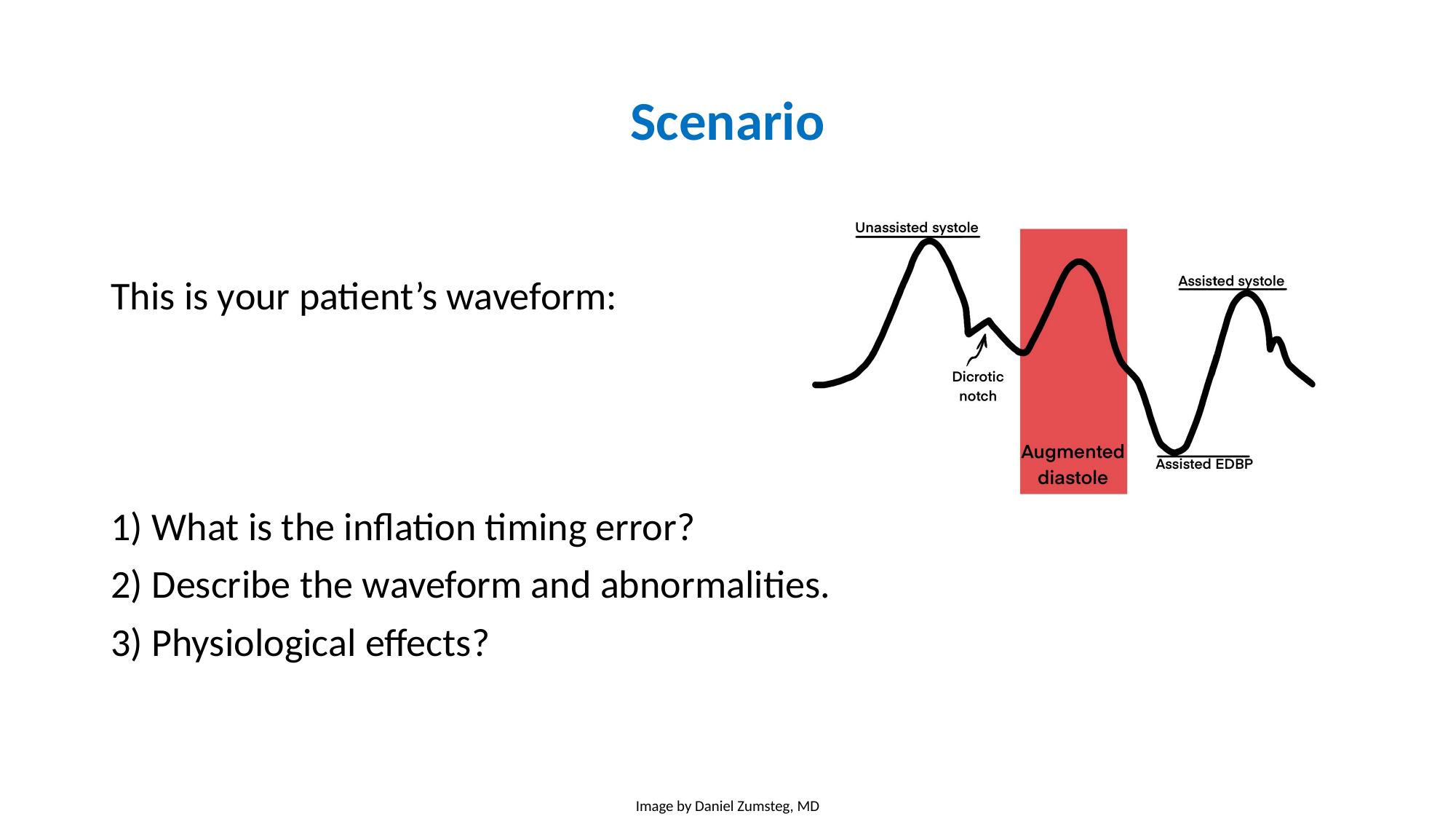

# Scenario
This is your patient’s waveform:
1) What is the inflation timing error?
2) Describe the waveform and abnormalities.
3) Physiological effects?
Image by Daniel Zumsteg, MD

## Slide 20
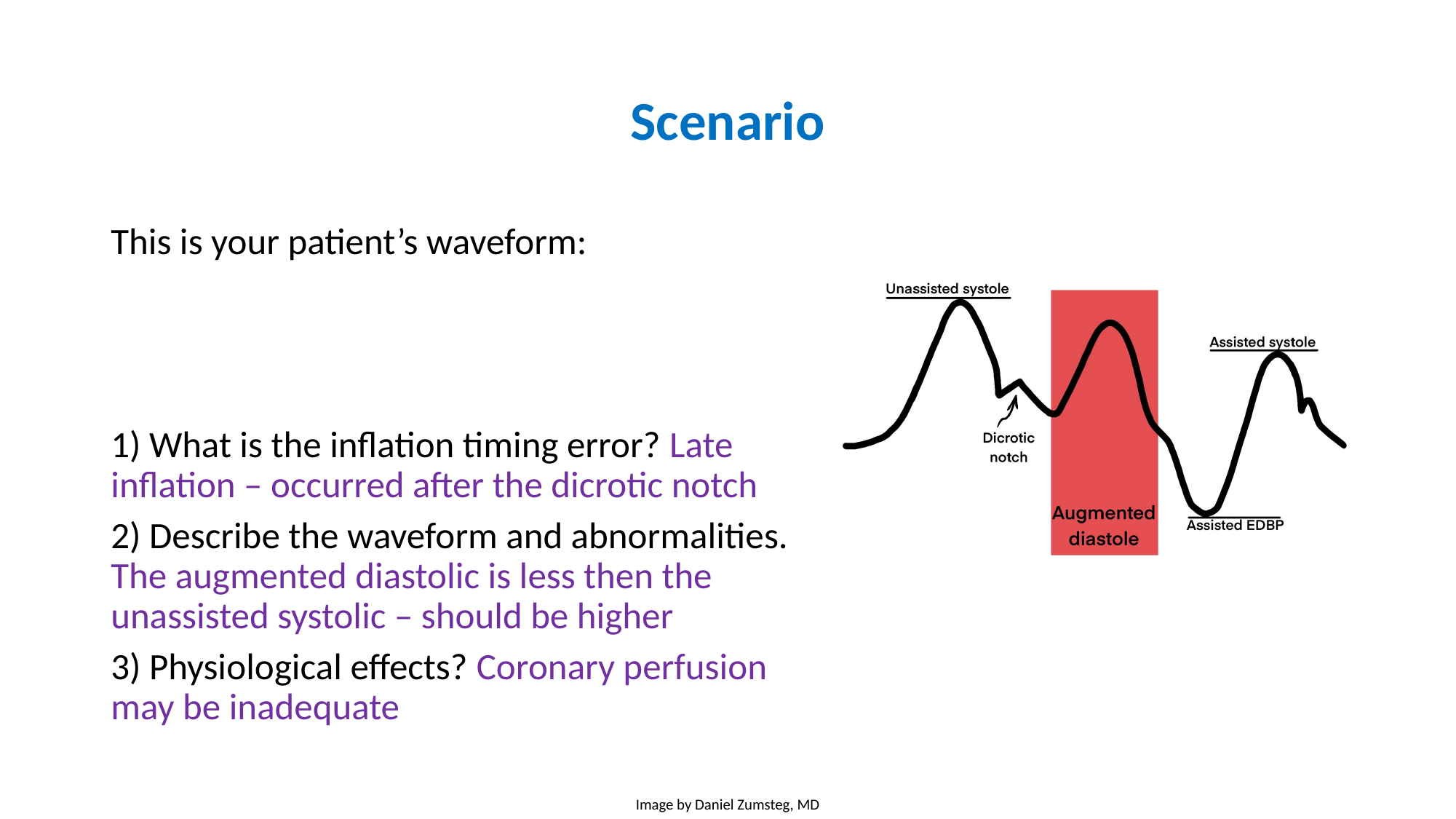

# Scenario
This is your patient’s waveform:
1) What is the inflation timing error? Late inflation – occurred after the dicrotic notch
2) Describe the waveform and abnormalities. The augmented diastolic is less then the unassisted systolic – should be higher
3) Physiological effects? Coronary perfusion may be inadequate
Image by Daniel Zumsteg, MD

## Slide 21
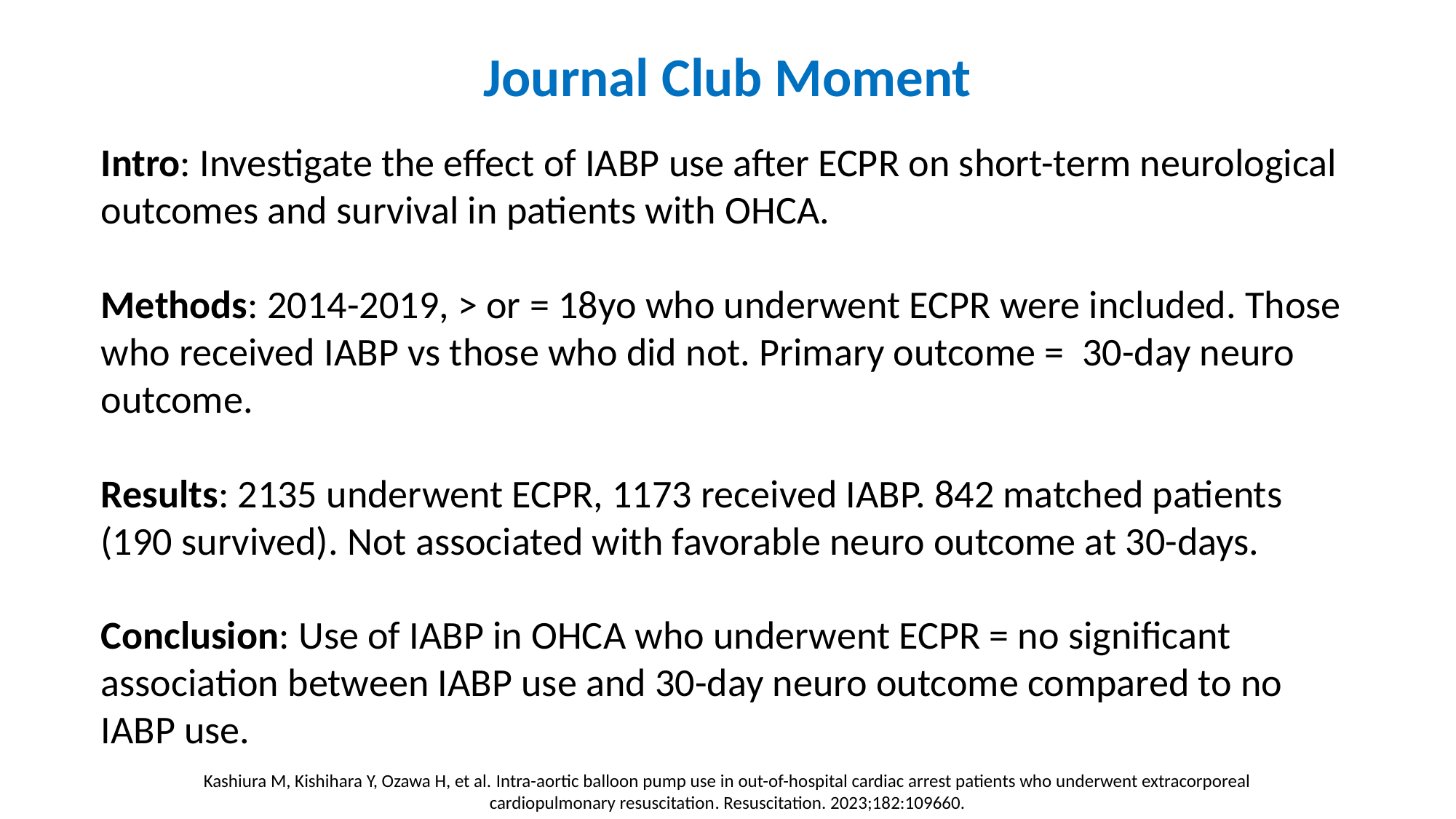

# Journal Club Moment
Intro: Investigate the effect of IABP use after ECPR on short-term neurological outcomes and survival in patients with OHCA.
Methods: 2014-2019, > or = 18yo who underwent ECPR were included. Those who received IABP vs those who did not. Primary outcome = 30-day neuro outcome.
Results: 2135 underwent ECPR, 1173 received IABP. 842 matched patients (190 survived). Not associated with favorable neuro outcome at 30-days.
Conclusion: Use of IABP in OHCA who underwent ECPR = no significant association between IABP use and 30-day neuro outcome compared to no IABP use.
Kashiura M, Kishihara Y, Ozawa H, et al. Intra-aortic balloon pump use in out-of-hospital cardiac arrest patients who underwent extracorporeal cardiopulmonary resuscitation. Resuscitation. 2023;182:109660.

## Slide 22
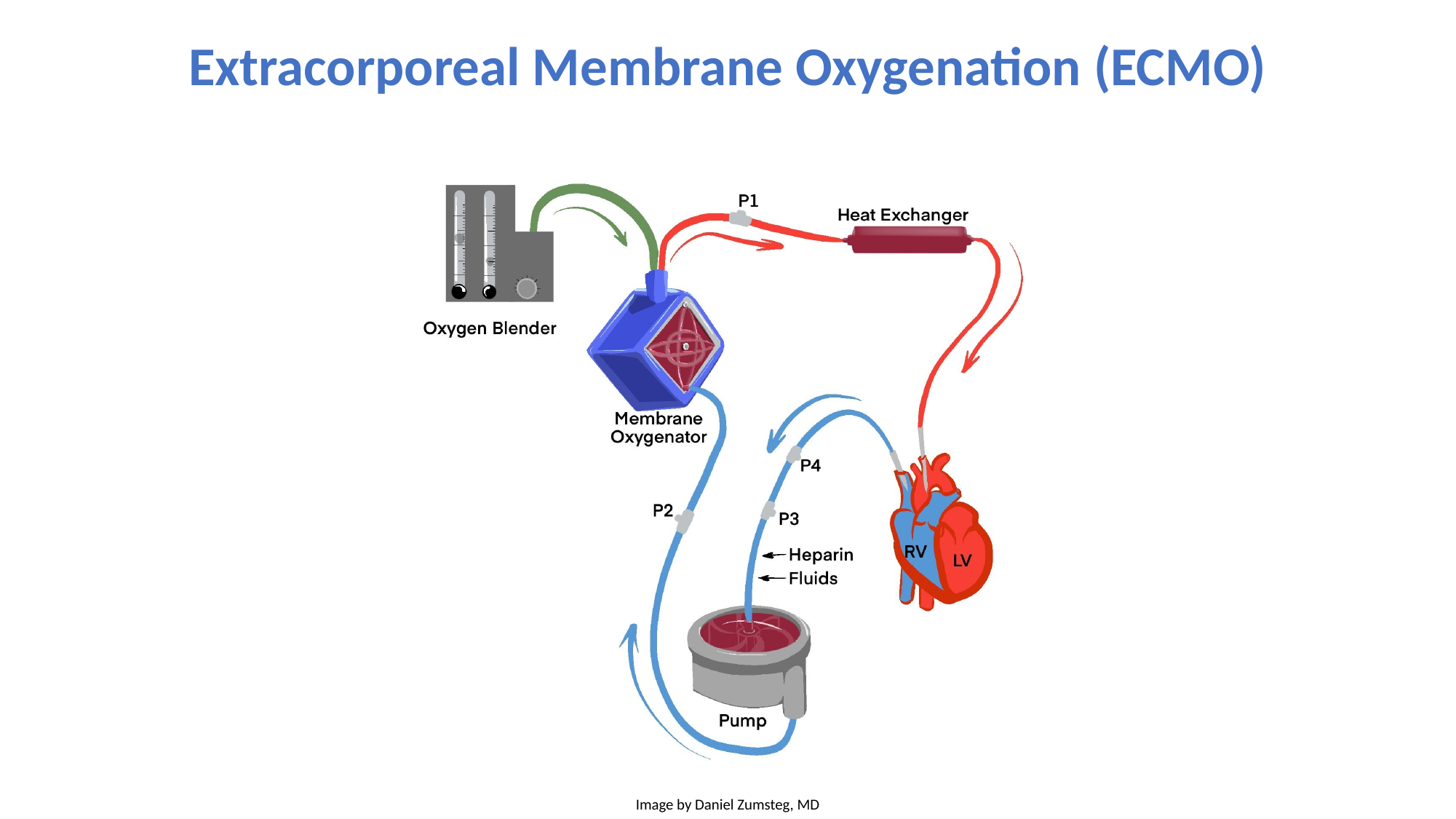

Extracorporeal Membrane Oxygenation (ECMO)
Image by Daniel Zumsteg, MD

## Slide 23
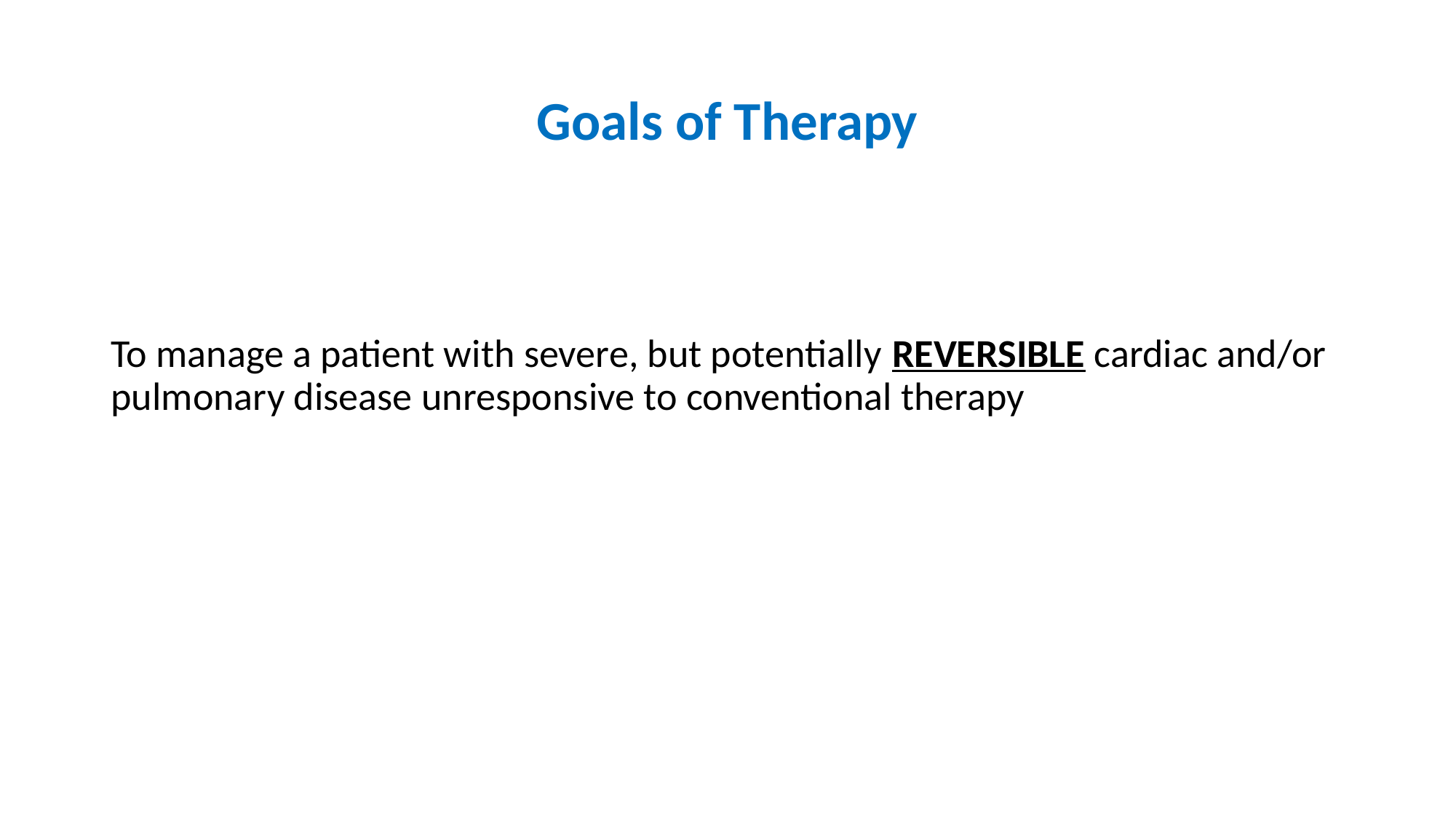

# Goals of Therapy
To manage a patient with severe, but potentially REVERSIBLE cardiac and/or pulmonary disease unresponsive to conventional therapy

## Slide 24
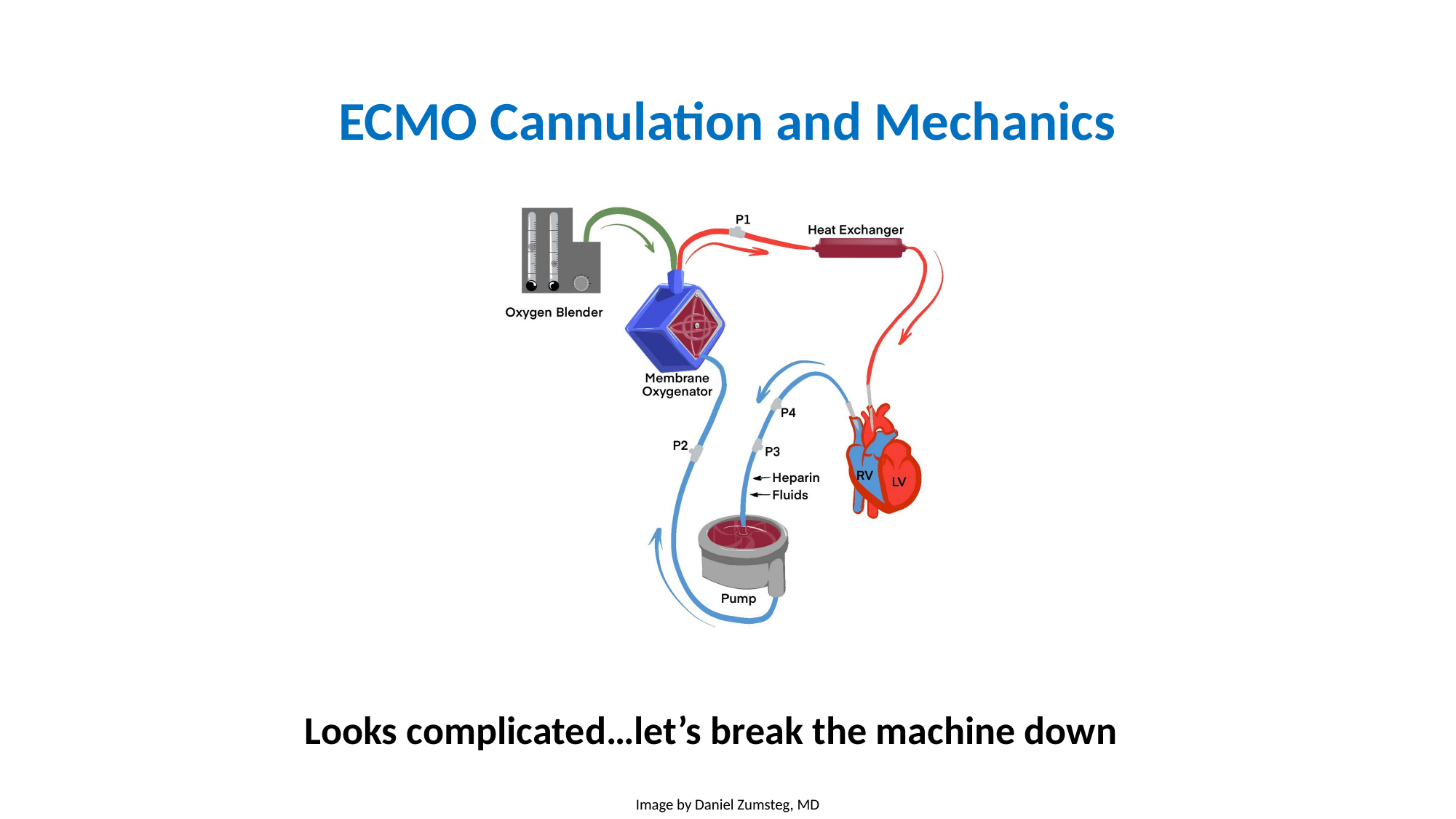

# ECMO Cannulation and Mechanics
Looks complicated…let’s break the machine down
Image by Daniel Zumsteg, MD

## Slide 25
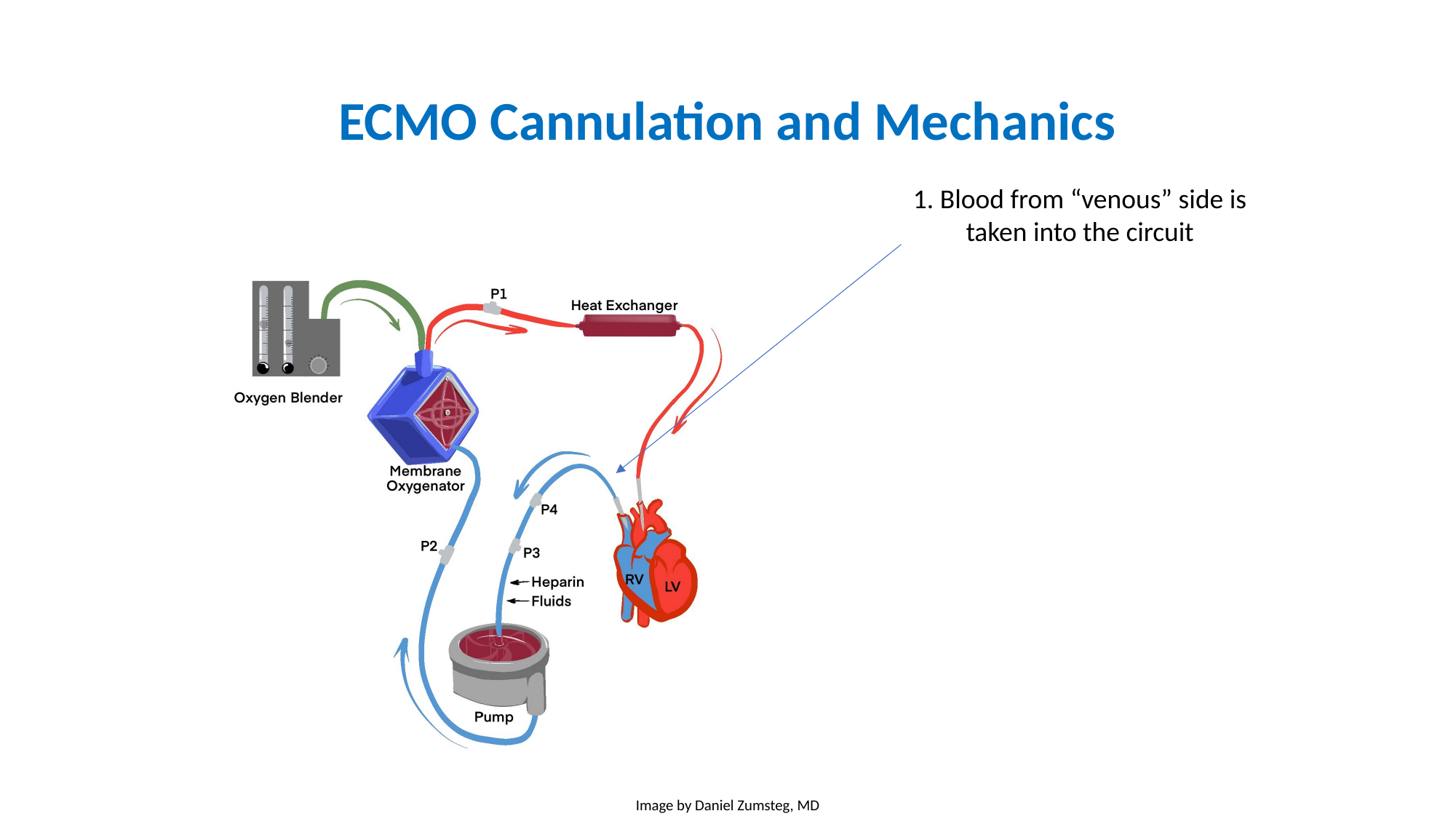

# ECMO Cannulation and Mechanics
1. Blood from “venous” side is taken into the circuit
Image by Daniel Zumsteg, MD

## Slide 26
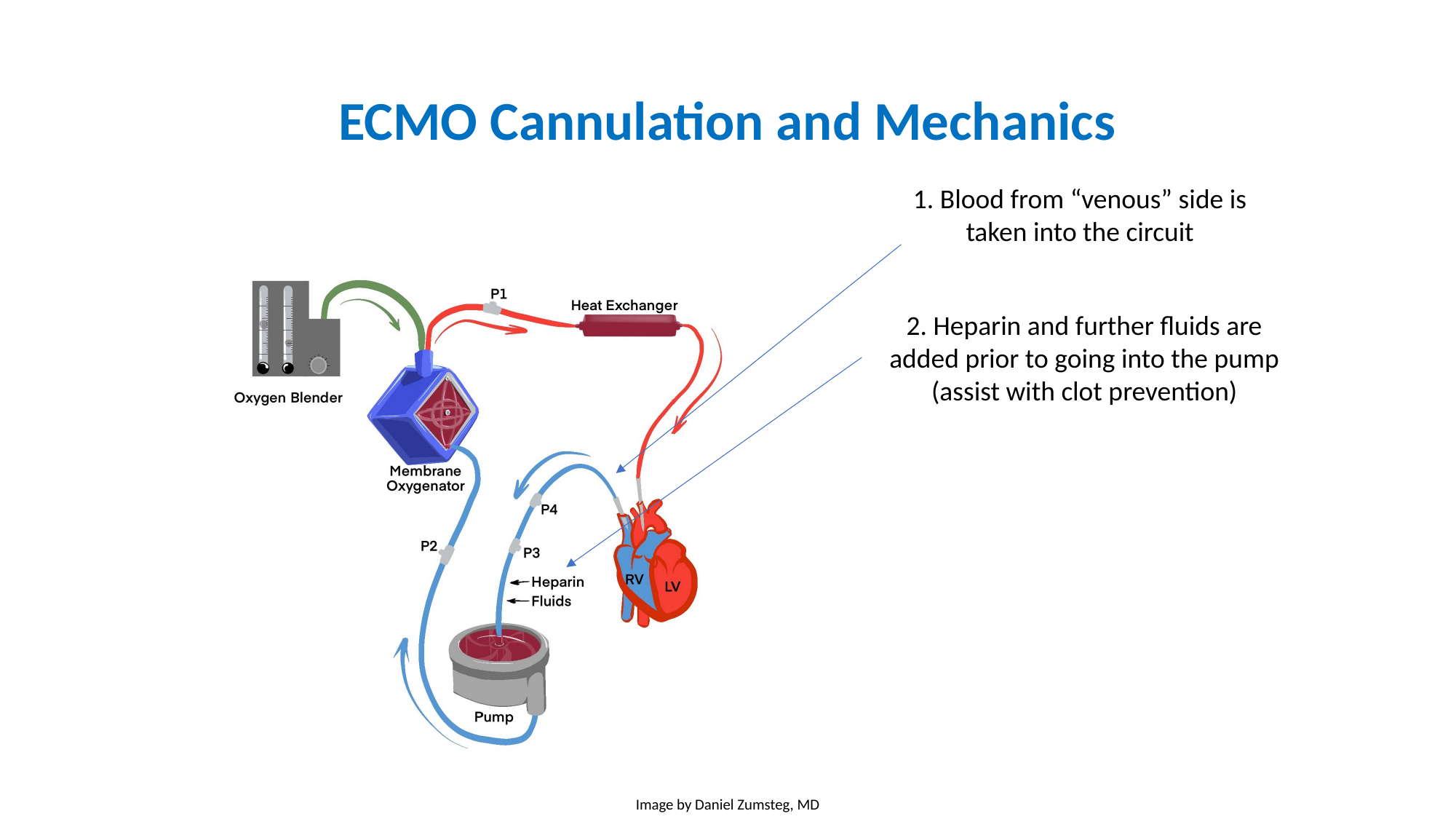

# ECMO Cannulation and Mechanics
1. Blood from “venous” side is taken into the circuit
2. Heparin and further fluids are added prior to going into the pump (assist with clot prevention)
Image by Daniel Zumsteg, MD

## Slide 27
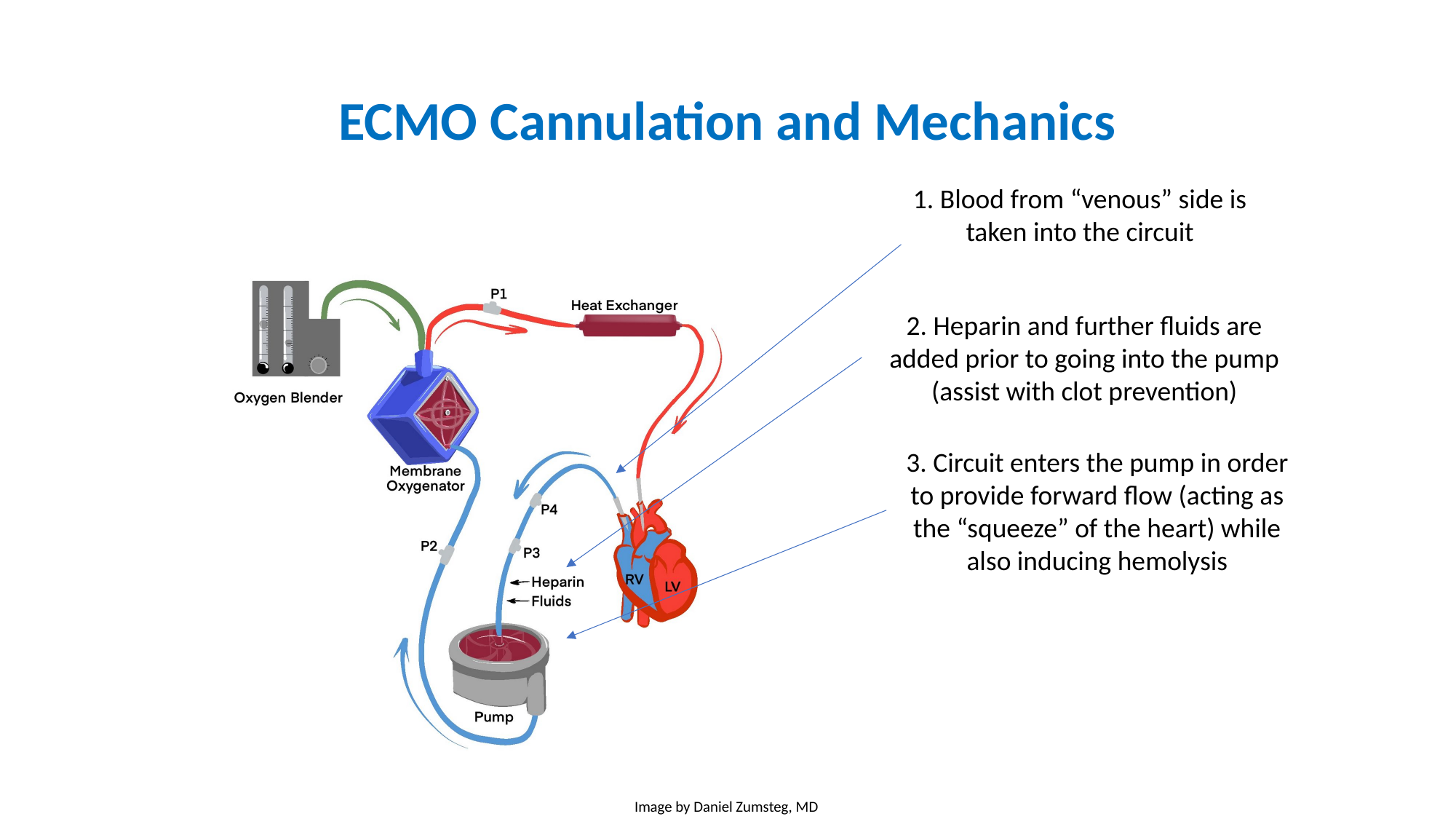

# ECMO Cannulation and Mechanics
1. Blood from “venous” side is taken into the circuit
2. Heparin and further fluids are added prior to going into the pump (assist with clot prevention)
3. Circuit enters the pump in order to provide forward flow (acting as the “squeeze” of the heart) while also inducing hemolysis
Image by Daniel Zumsteg, MD

## Slide 28
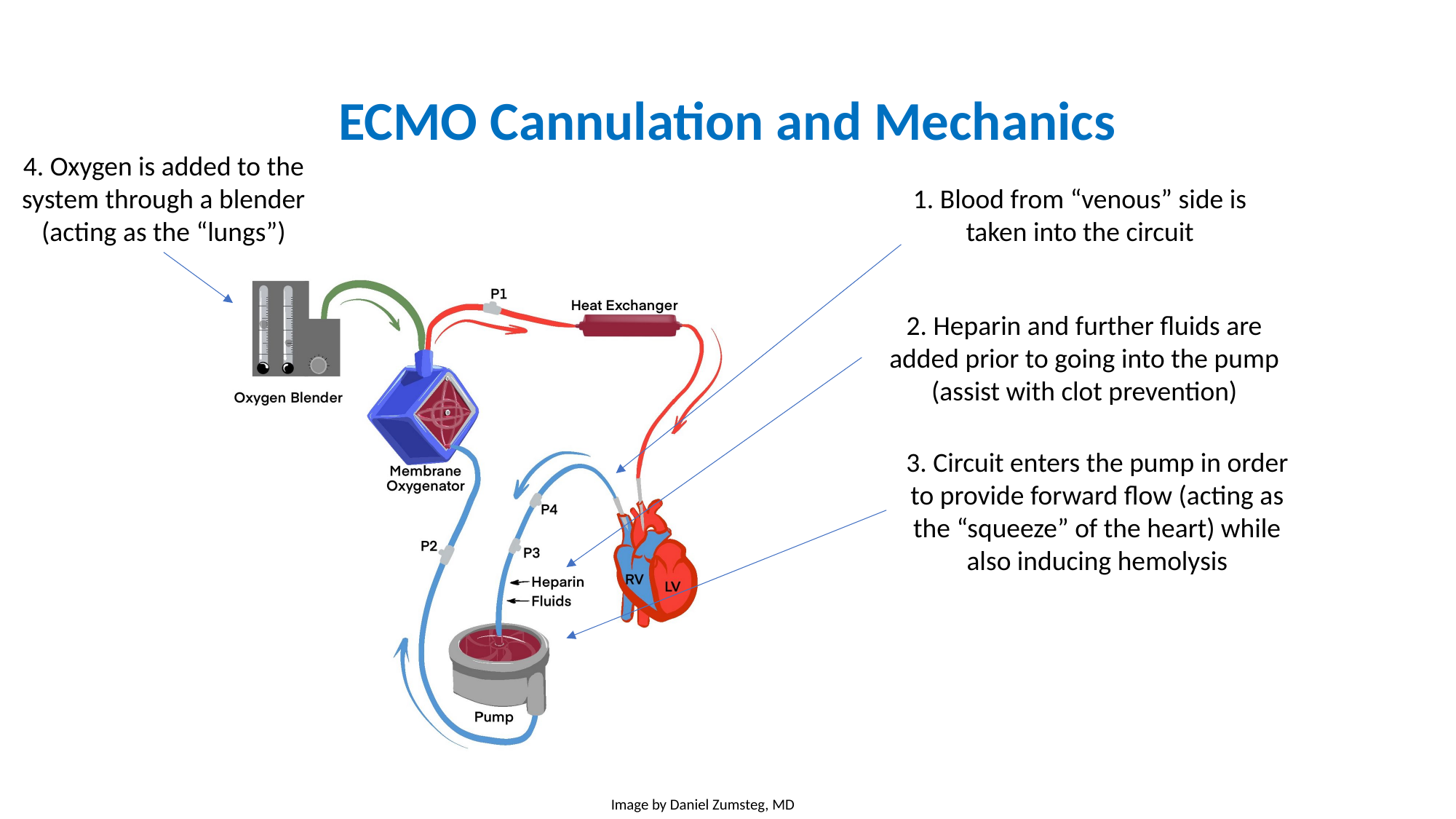

# ECMO Cannulation and Mechanics
4. Oxygen is added to the system through a blender (acting as the “lungs”)
1. Blood from “venous” side is taken into the circuit
2. Heparin and further fluids are added prior to going into the pump (assist with clot prevention)
3. Circuit enters the pump in order to provide forward flow (acting as the “squeeze” of the heart) while also inducing hemolysis
Image by Daniel Zumsteg, MD

## Slide 29
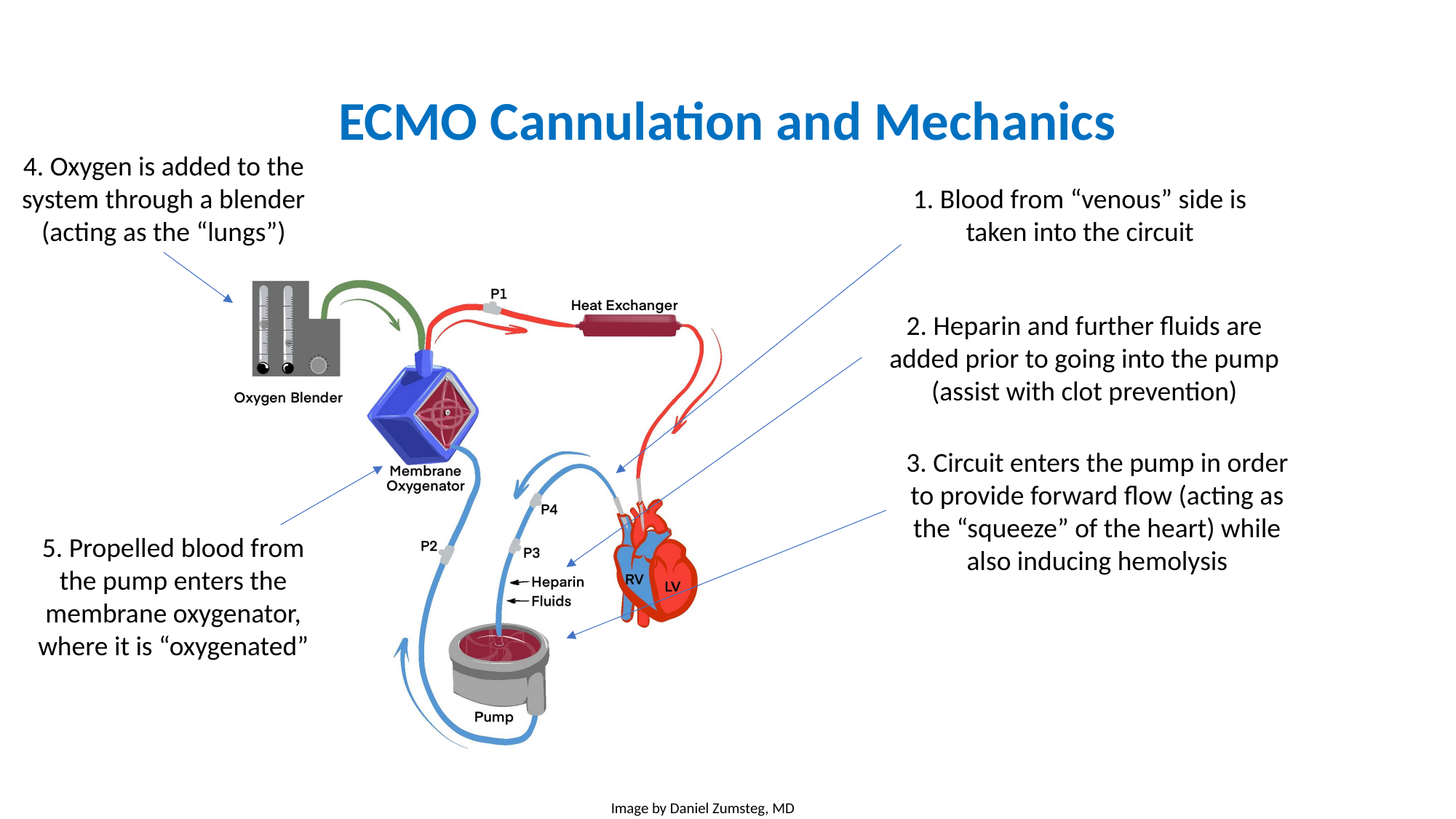

# ECMO Cannulation and Mechanics
4. Oxygen is added to the system through a blender (acting as the “lungs”)
1. Blood from “venous” side is taken into the circuit
2. Heparin and further fluids are added prior to going into the pump (assist with clot prevention)
3. Circuit enters the pump in order to provide forward flow (acting as the “squeeze” of the heart) while also inducing hemolysis
5. Propelled blood from the pump enters the membrane oxygenator, where it is “oxygenated”
Image by Daniel Zumsteg, MD

## Slide 30
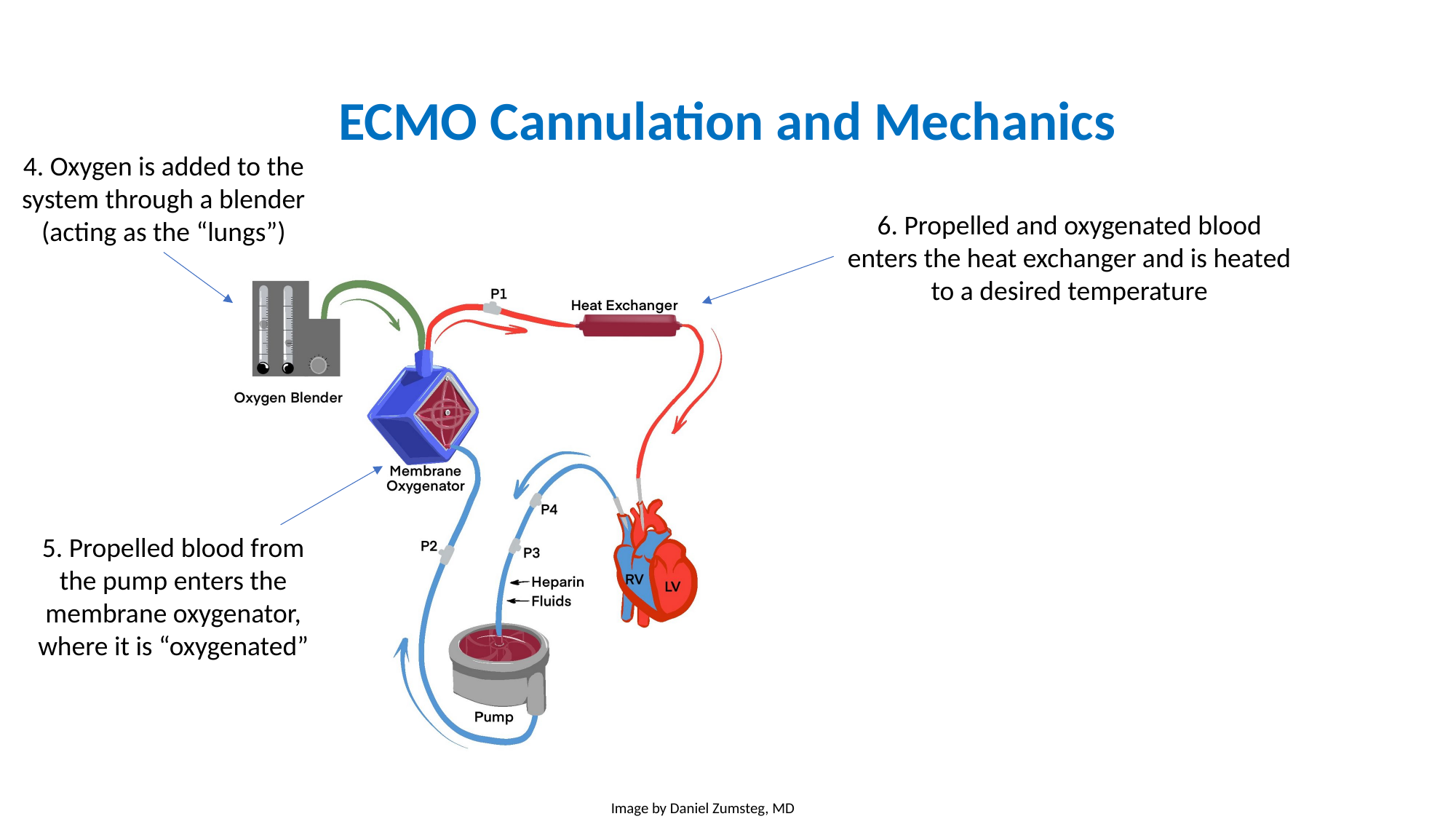

# ECMO Cannulation and Mechanics
4. Oxygen is added to the system through a blender (acting as the “lungs”)
6. Propelled and oxygenated blood enters the heat exchanger and is heated to a desired temperature
5. Propelled blood from the pump enters the membrane oxygenator, where it is “oxygenated”
Image by Daniel Zumsteg, MD

## Slide 31
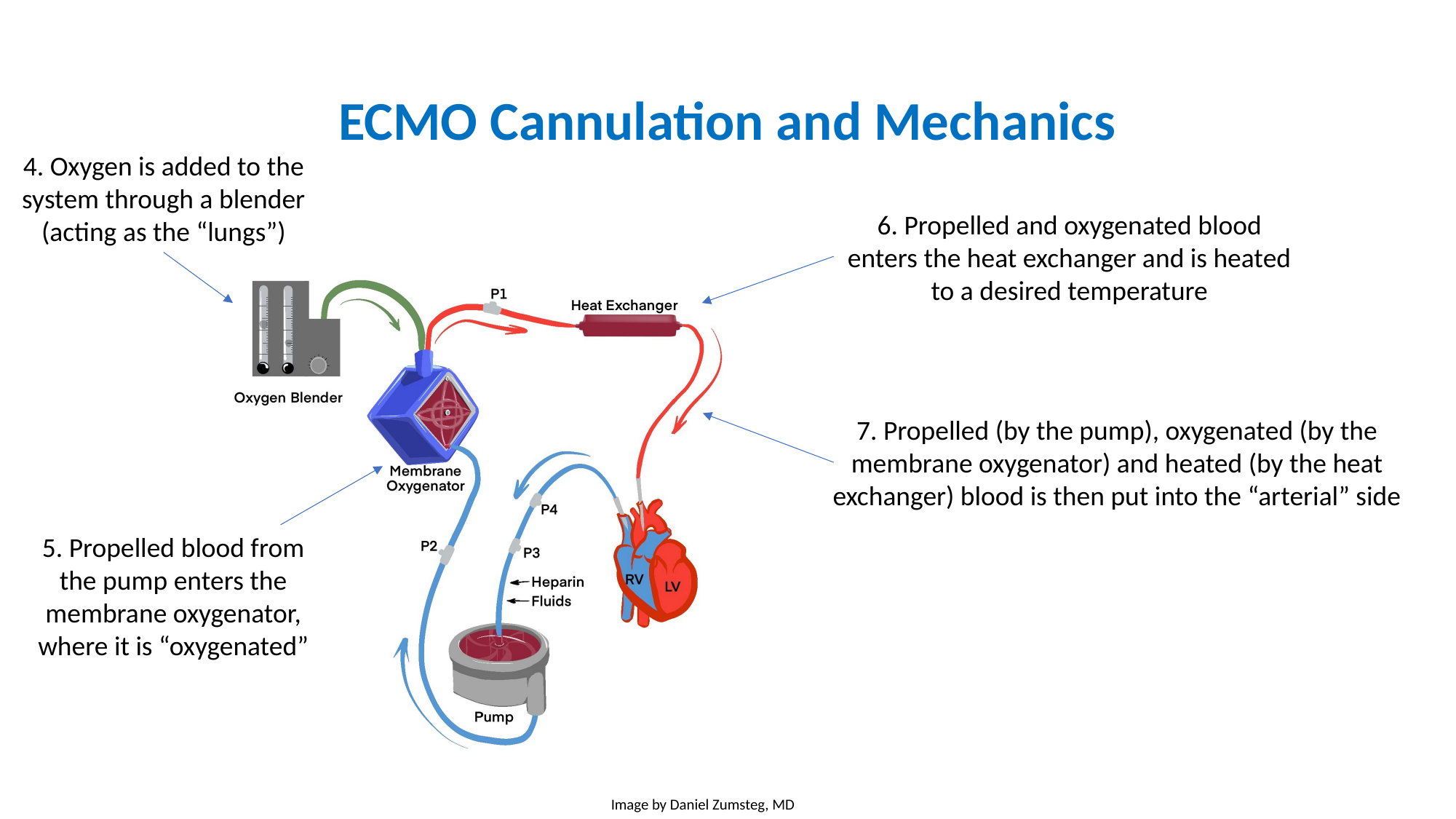

# ECMO Cannulation and Mechanics
4. Oxygen is added to the system through a blender (acting as the “lungs”)
6. Propelled and oxygenated blood enters the heat exchanger and is heated to a desired temperature
7. Propelled (by the pump), oxygenated (by the membrane oxygenator) and heated (by the heat exchanger) blood is then put into the “arterial” side
5. Propelled blood from the pump enters the membrane oxygenator, where it is “oxygenated”
Image by Daniel Zumsteg, MD

## Slide 32
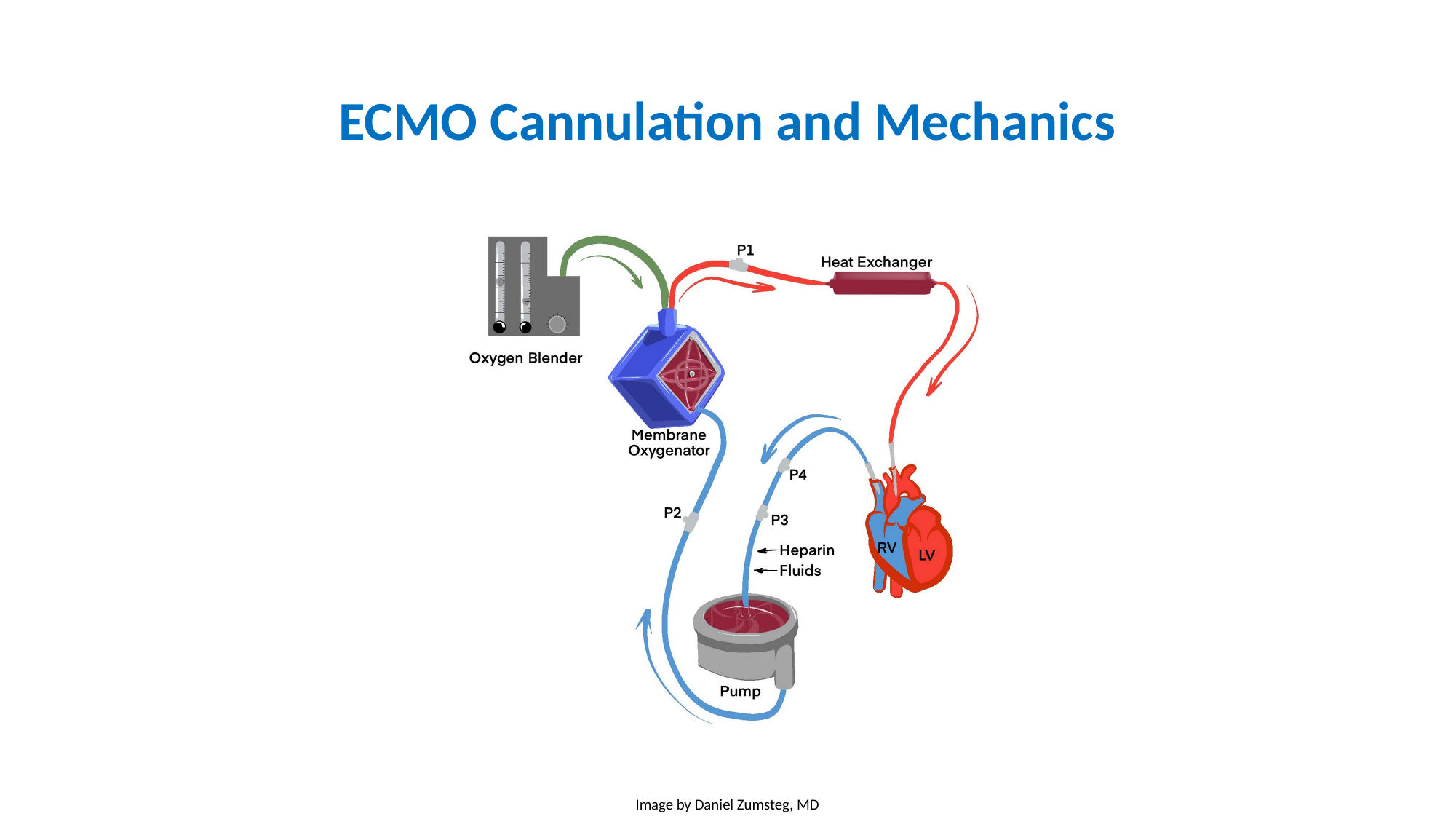

# ECMO Cannulation and Mechanics
Image by Daniel Zumsteg, MD

## Slide 33
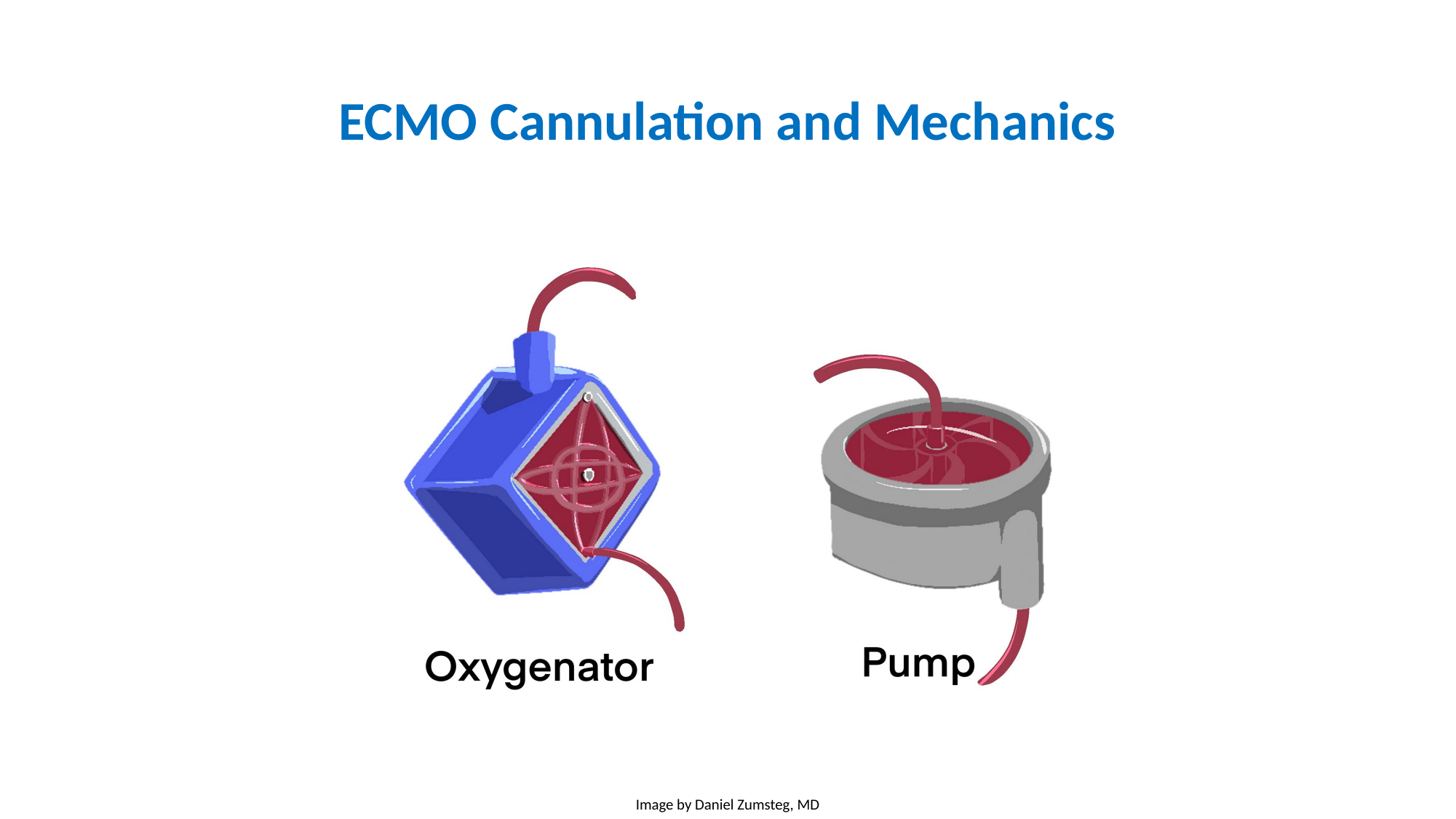

# ECMO Cannulation and Mechanics
Image by Daniel Zumsteg, MD

## Slide 34
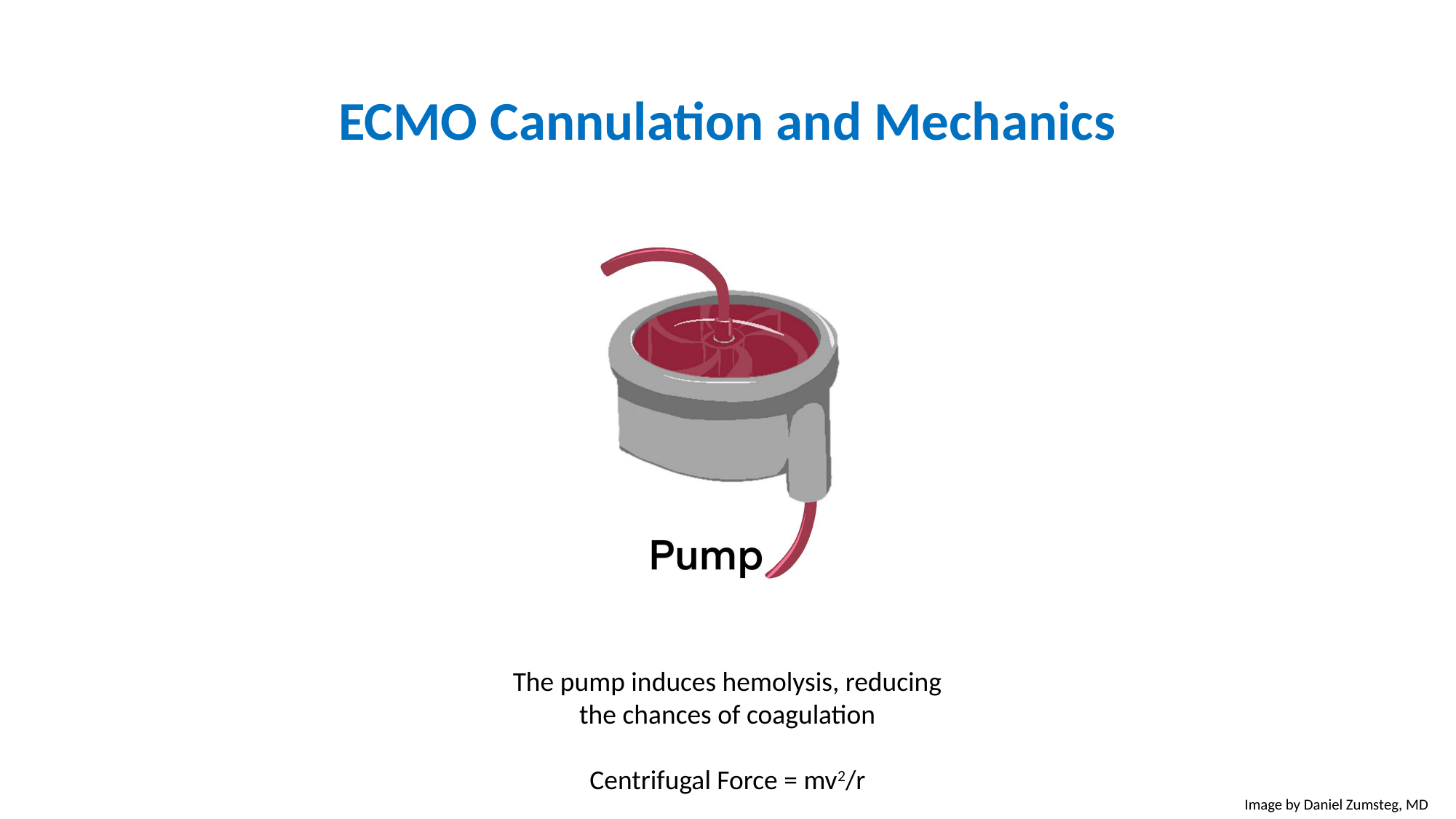

# ECMO Cannulation and Mechanics
The pump induces hemolysis, reducing the chances of coagulation
Centrifugal Force = mv2/r
Image by Daniel Zumsteg, MD

## Slide 35
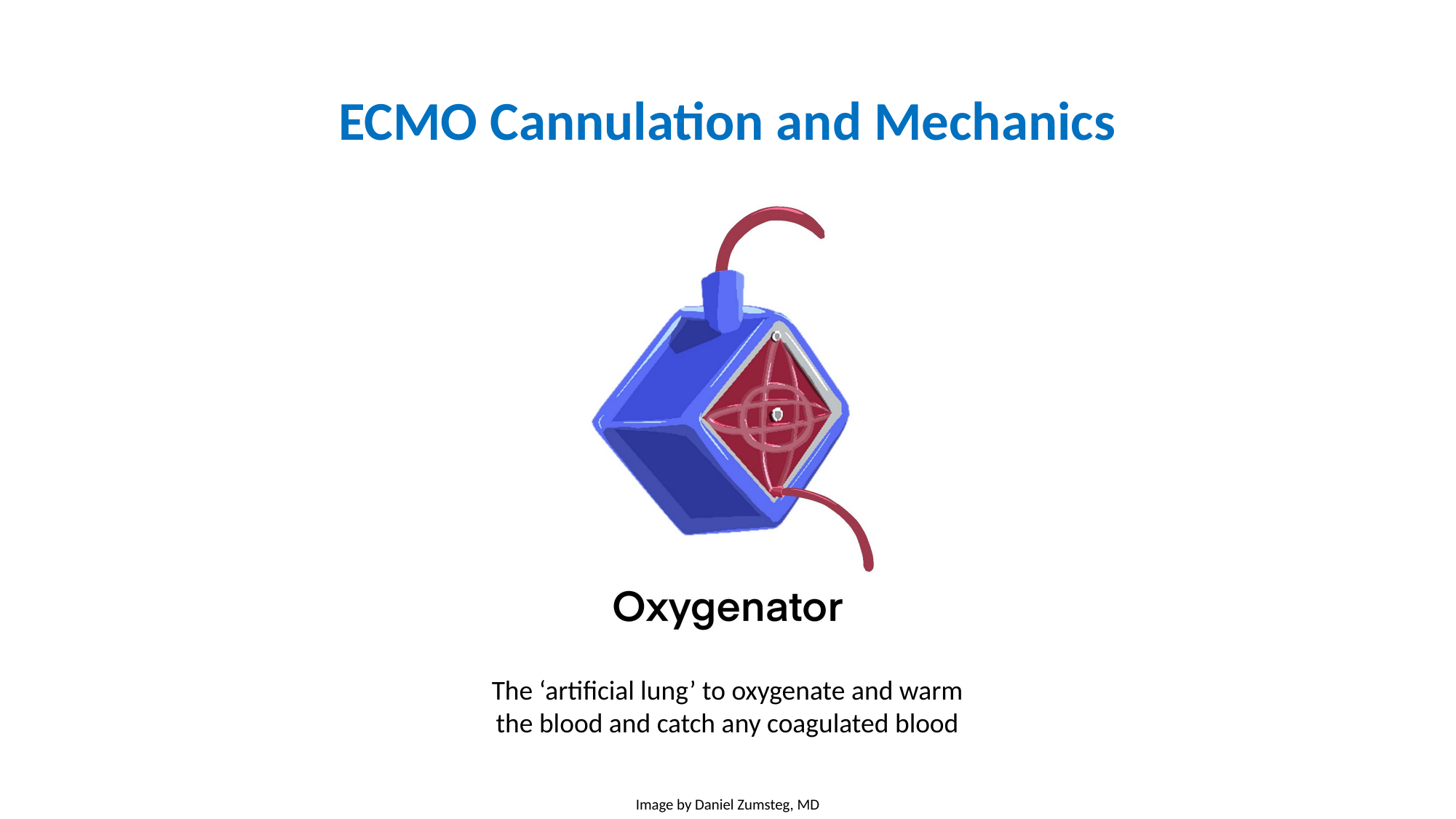

# ECMO Cannulation and Mechanics
The ‘artificial lung’ to oxygenate and warm the blood and catch any coagulated blood
Image by Daniel Zumsteg, MD

## Slide 36
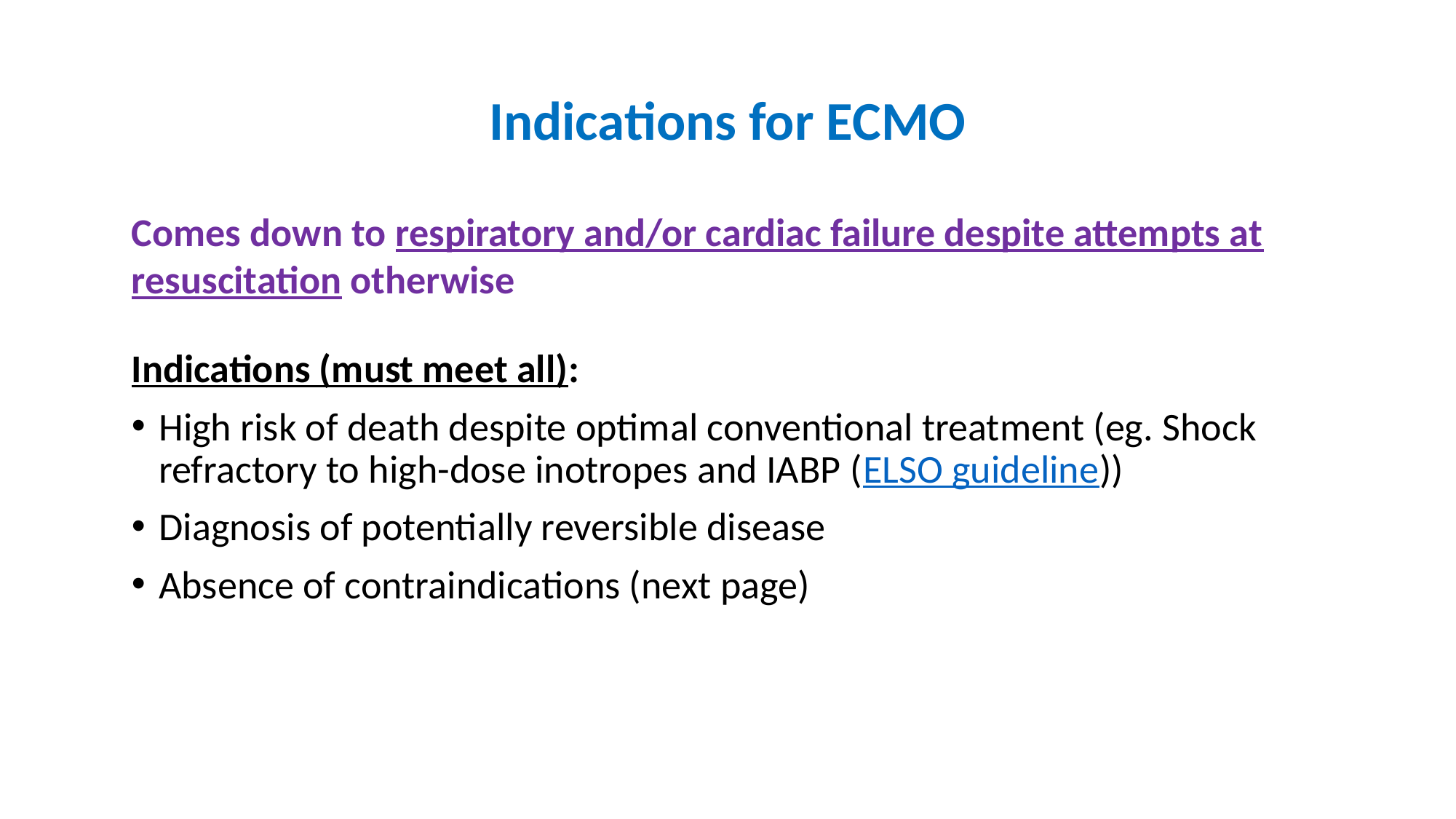

# Indications for ECMO
Comes down to respiratory and/or cardiac failure despite attempts at resuscitation otherwise
Indications (must meet all):
High risk of death despite optimal conventional treatment (eg. Shock refractory to high-dose inotropes and IABP (ELSO guideline))
Diagnosis of potentially reversible disease
Absence of contraindications (next page)

## Slide 37
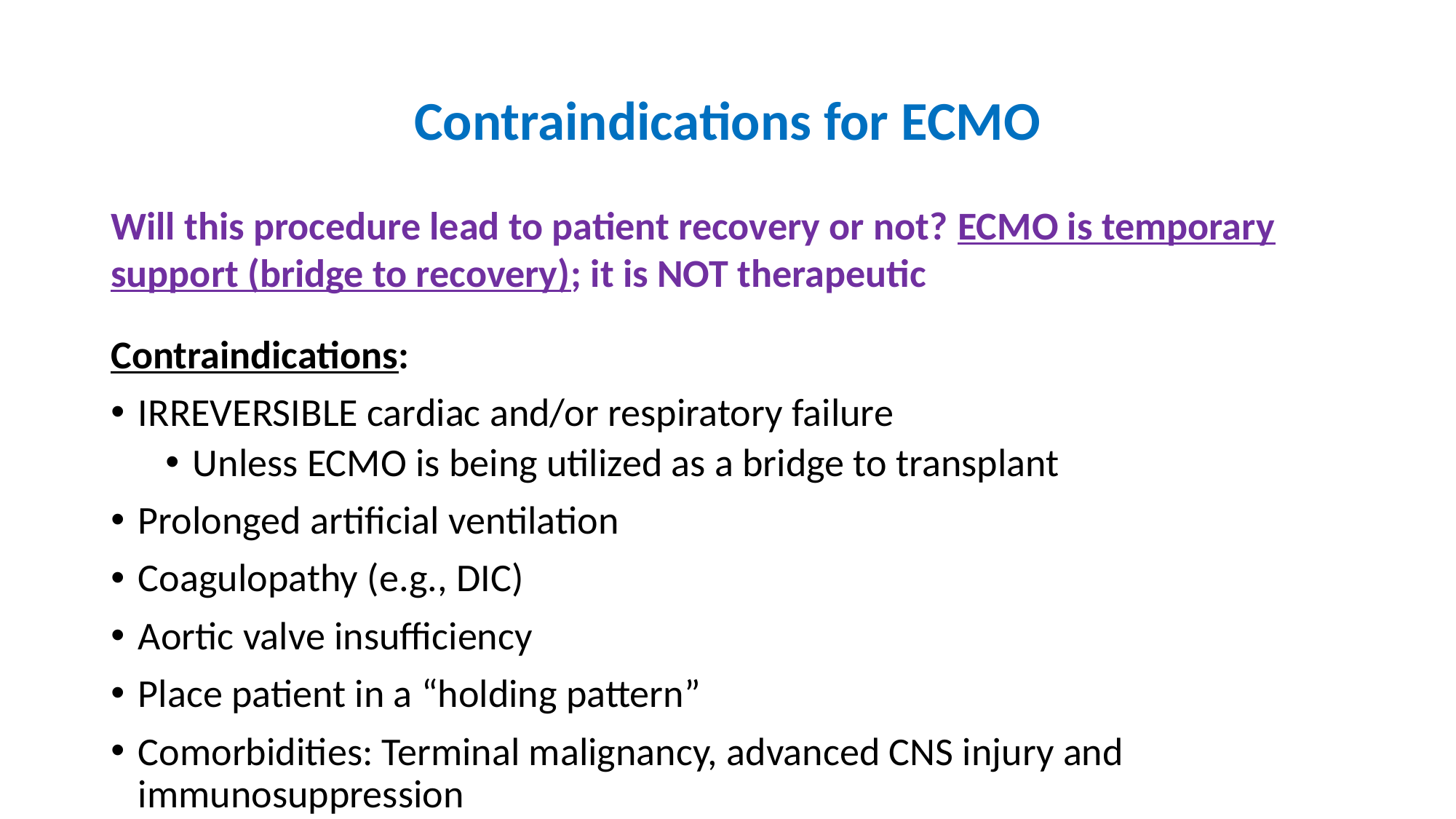

# Contraindications for ECMO
Will this procedure lead to patient recovery or not? ECMO is temporary support (bridge to recovery); it is NOT therapeutic
Contraindications:
IRREVERSIBLE cardiac and/or respiratory failure
Unless ECMO is being utilized as a bridge to transplant
Prolonged artificial ventilation
Coagulopathy (e.g., DIC)
Aortic valve insufficiency
Place patient in a “holding pattern”
Comorbidities: Terminal malignancy, advanced CNS injury and immunosuppression

## Slide 38
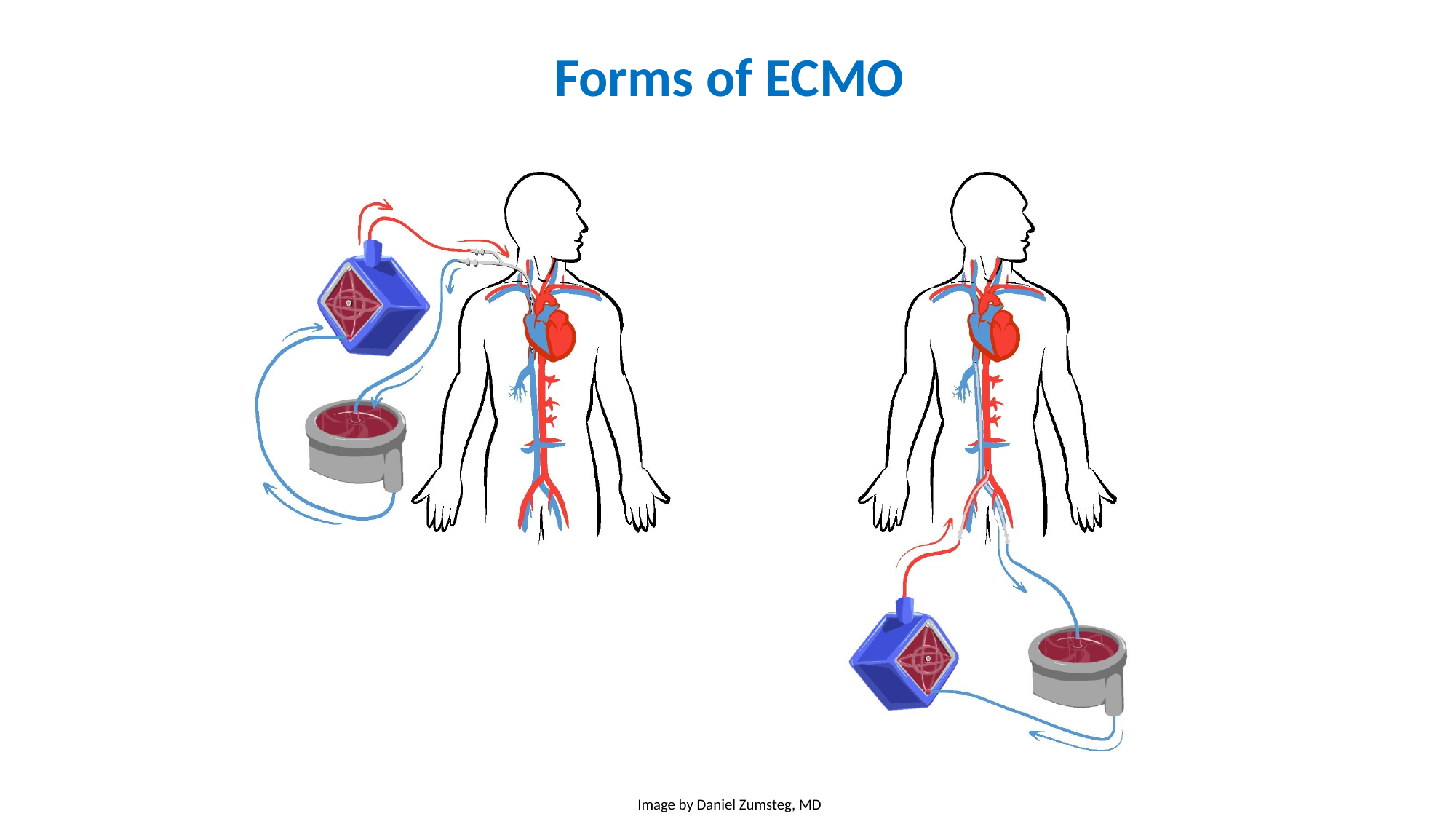

# Forms of ECMO
Image by Daniel Zumsteg, MD

## Slide 39
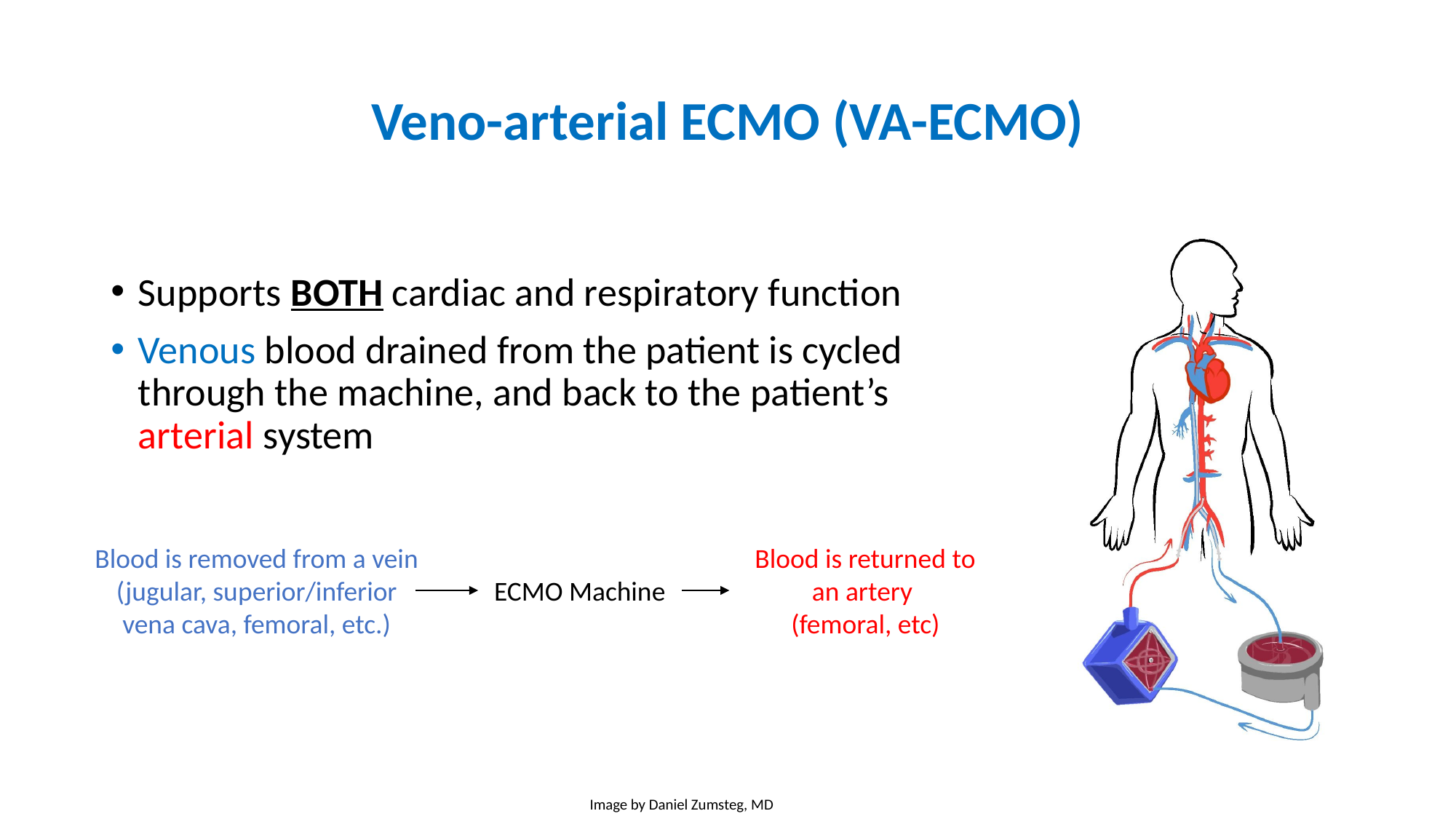

# Veno-arterial ECMO (VA-ECMO)
Supports BOTH cardiac and respiratory function
Venous blood drained from the patient is cycled through the machine, and back to the patient’s arterial system
Blood is removed from a vein (jugular, superior/inferior vena cava, femoral, etc.)
Blood is returned to an artery
(femoral, etc)
ECMO Machine
Image by Daniel Zumsteg, MD

## Slide 40
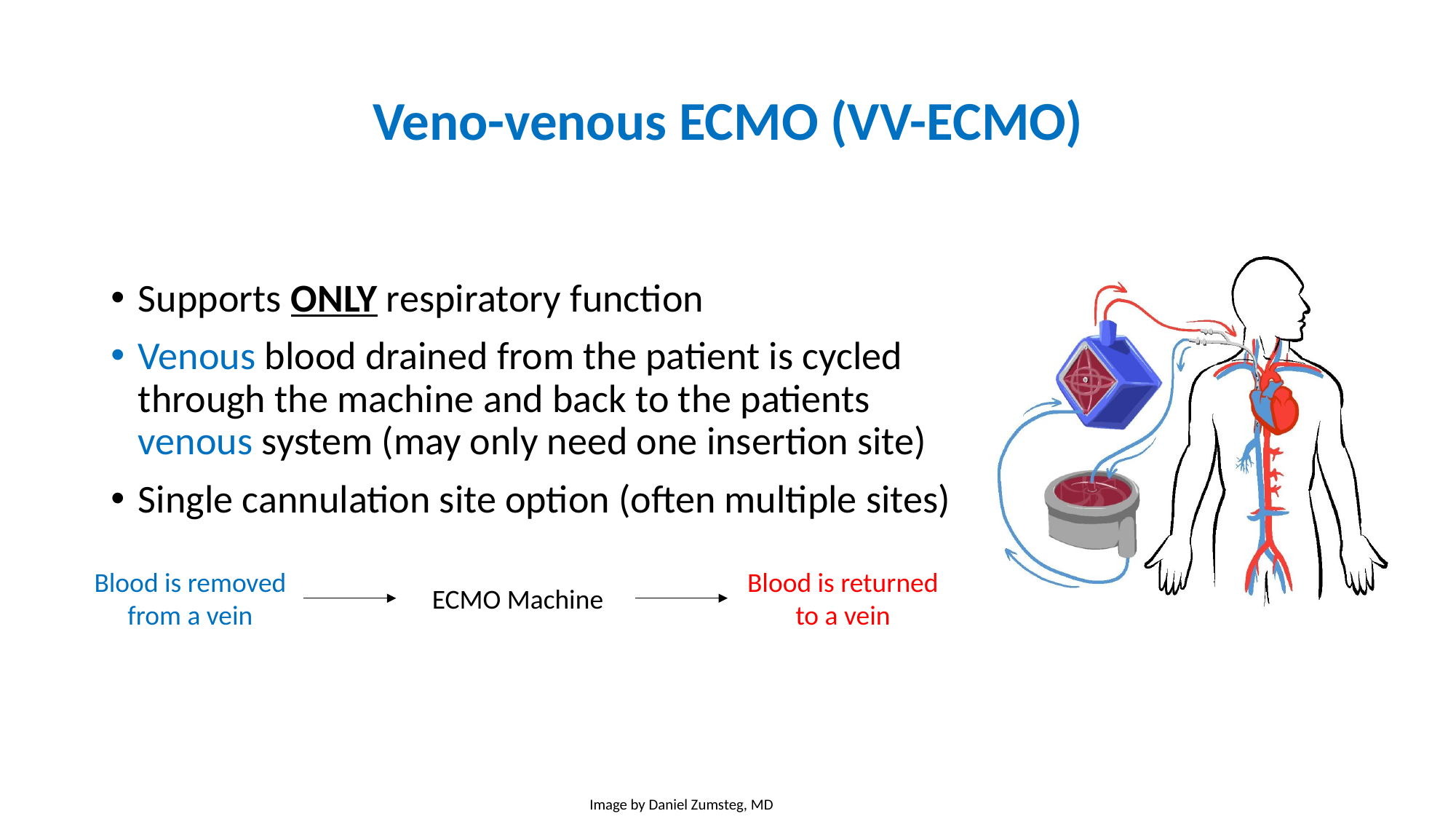

# Veno-venous ECMO (VV-ECMO)
Supports ONLY respiratory function
Venous blood drained from the patient is cycled through the machine and back to the patients venous system (may only need one insertion site)
Single cannulation site option (often multiple sites)
Blood is removed from a vein
Blood is returned to a vein
ECMO Machine
Image by Daniel Zumsteg, MD

## Slide 41
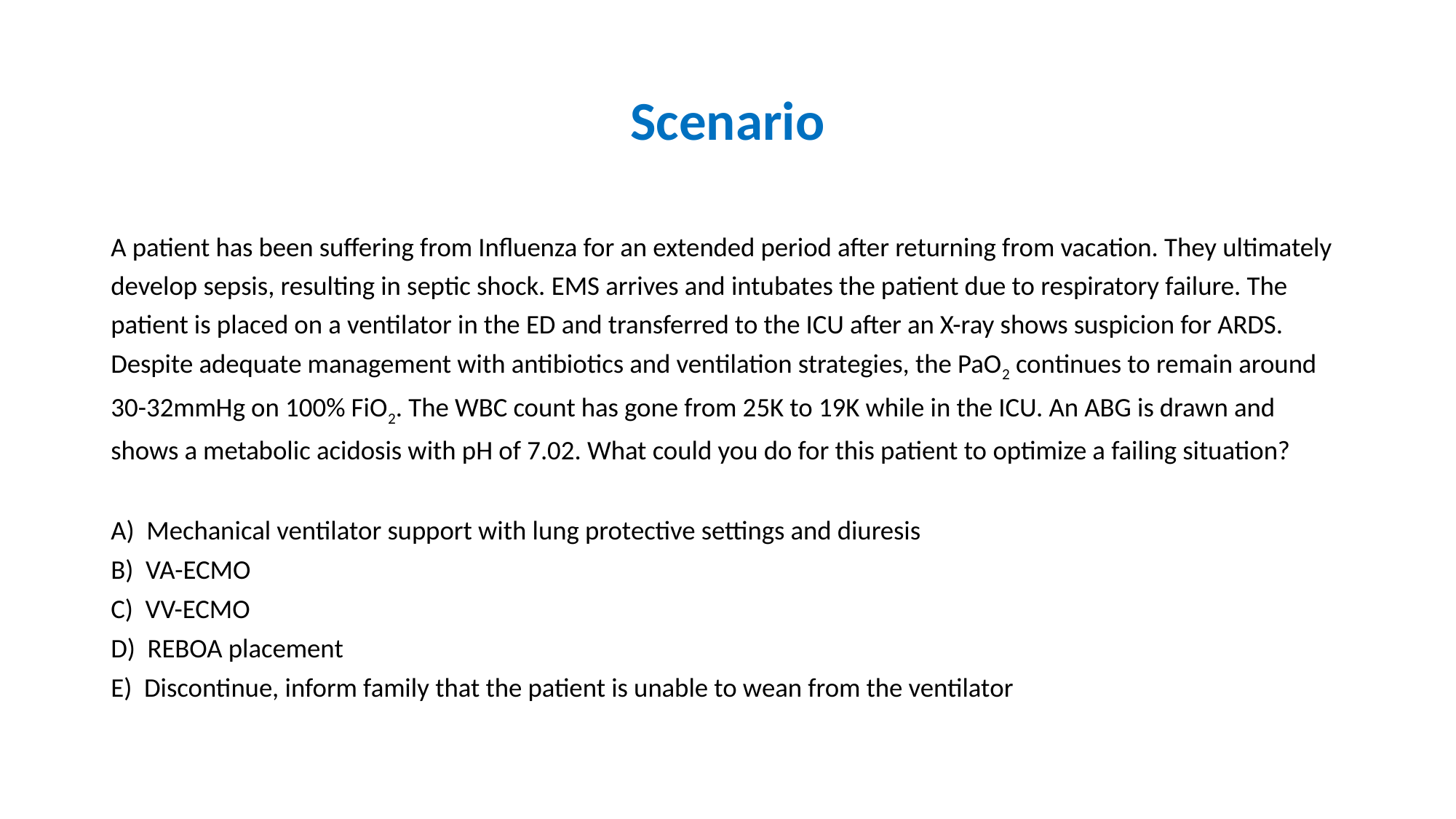

# Scenario
A patient has been suffering from Influenza for an extended period after returning from vacation. They ultimately develop sepsis, resulting in septic shock. EMS arrives and intubates the patient due to respiratory failure. The patient is placed on a ventilator in the ED and transferred to the ICU after an X-ray shows suspicion for ARDS. Despite adequate management with antibiotics and ventilation strategies, the PaO2 continues to remain around 30-32mmHg on 100% FiO2. The WBC count has gone from 25K to 19K while in the ICU. An ABG is drawn and shows a metabolic acidosis with pH of 7.02. What could you do for this patient to optimize a failing situation?
A)  Mechanical ventilator support with lung protective settings and diuresis
B)  VA-ECMO
C)  VV-ECMO
D) REBOA placement
E)  Discontinue, inform family that the patient is unable to wean from the ventilator

## Slide 42
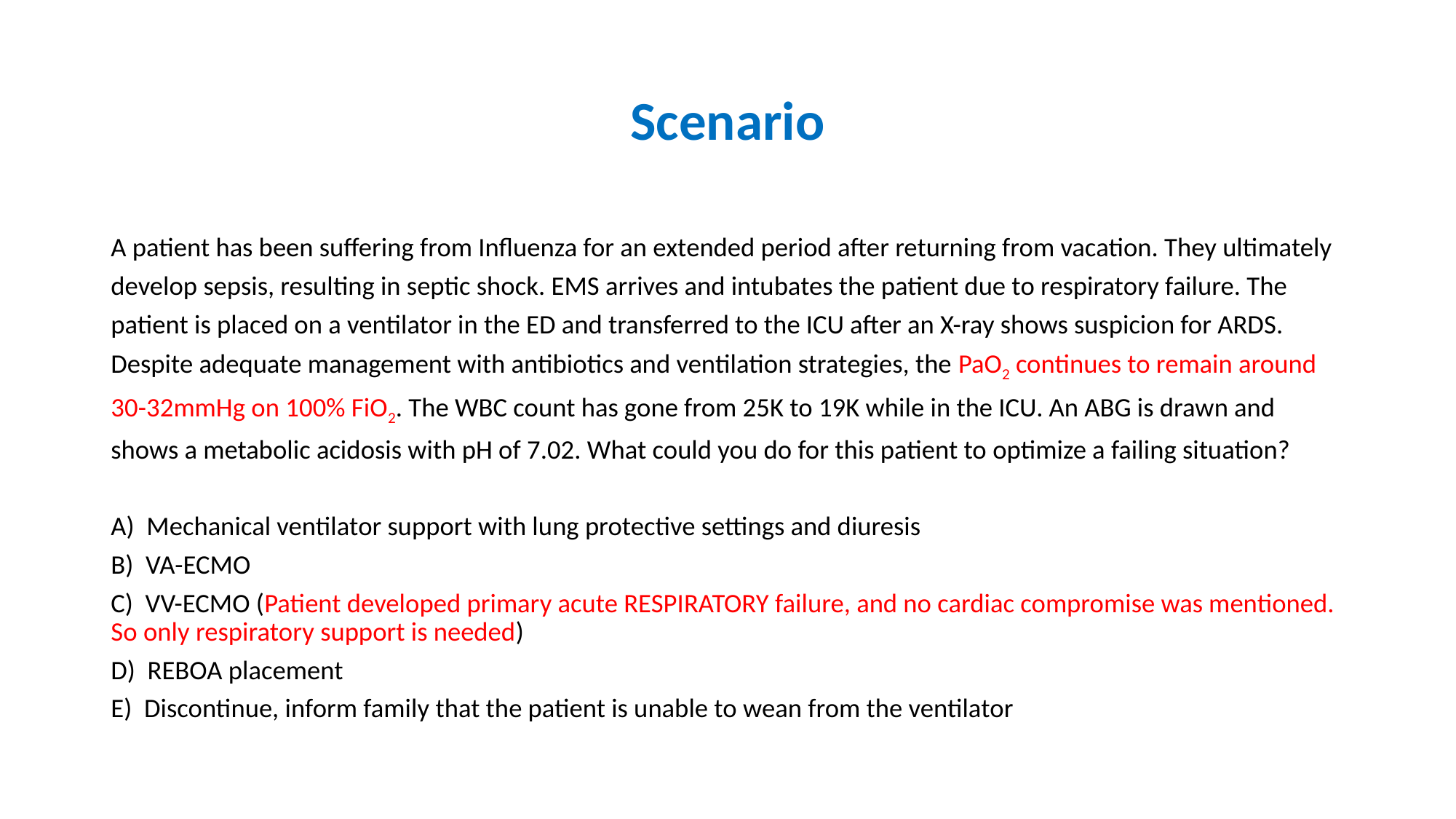

# Scenario
A patient has been suffering from Influenza for an extended period after returning from vacation. They ultimately develop sepsis, resulting in septic shock. EMS arrives and intubates the patient due to respiratory failure. The patient is placed on a ventilator in the ED and transferred to the ICU after an X-ray shows suspicion for ARDS. Despite adequate management with antibiotics and ventilation strategies, the PaO2 continues to remain around 30-32mmHg on 100% FiO2. The WBC count has gone from 25K to 19K while in the ICU. An ABG is drawn and shows a metabolic acidosis with pH of 7.02. What could you do for this patient to optimize a failing situation?
A)  Mechanical ventilator support with lung protective settings and diuresis
B)  VA-ECMO
C)  VV-ECMO (Patient developed primary acute RESPIRATORY failure, and no cardiac compromise was mentioned. So only respiratory support is needed)
D) REBOA placement
E)  Discontinue, inform family that the patient is unable to wean from the ventilator

## Slide 43
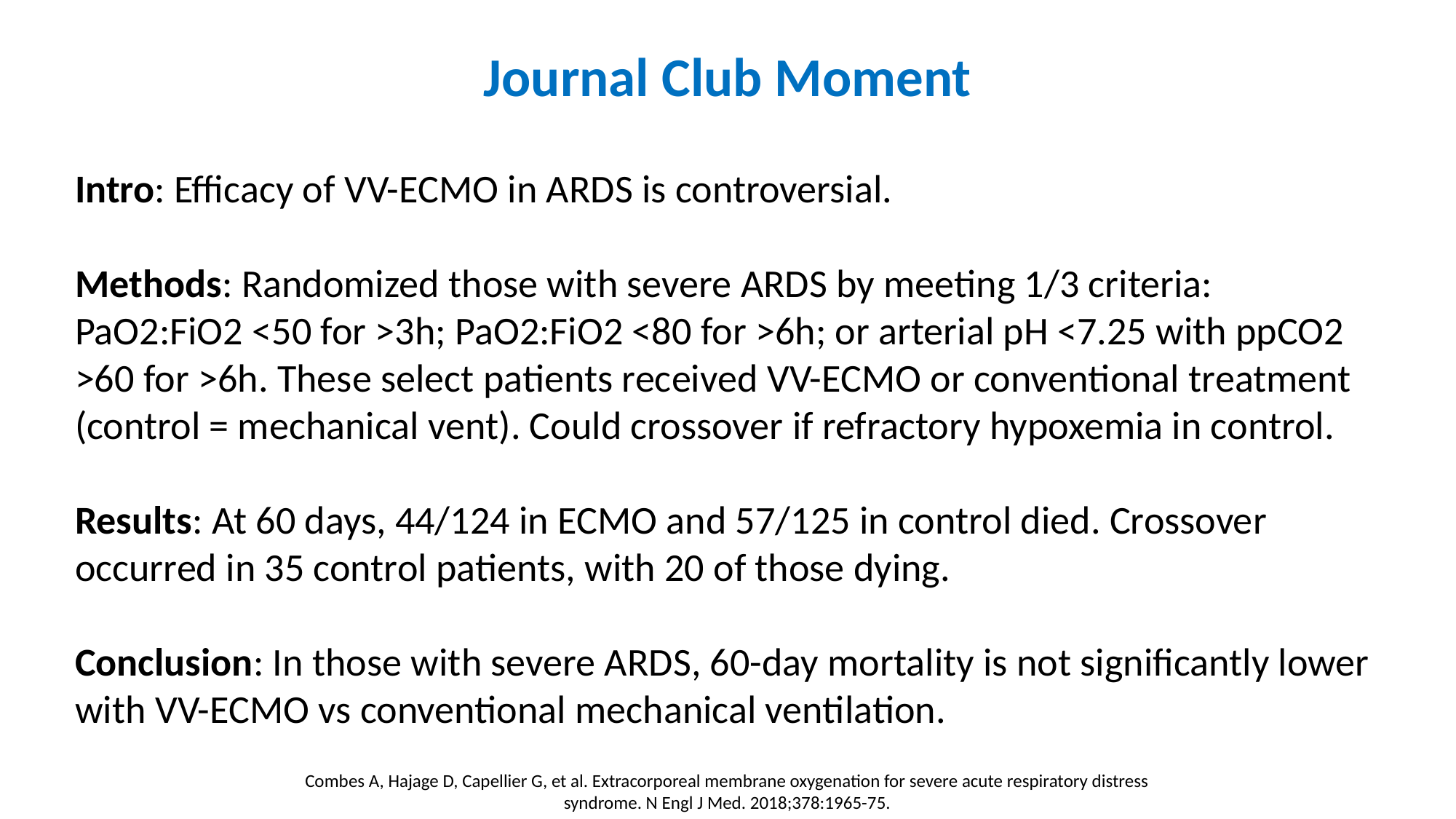

# Journal Club Moment
Intro: Efficacy of VV-ECMO in ARDS is controversial.
Methods: Randomized those with severe ARDS by meeting 1/3 criteria: PaO2:FiO2 <50 for >3h; PaO2:FiO2 <80 for >6h; or arterial pH <7.25 with ppCO2 >60 for >6h. These select patients received VV-ECMO or conventional treatment (control = mechanical vent). Could crossover if refractory hypoxemia in control.
Results: At 60 days, 44/124 in ECMO and 57/125 in control died. Crossover occurred in 35 control patients, with 20 of those dying.
Conclusion: In those with severe ARDS, 60-day mortality is not significantly lower with VV-ECMO vs conventional mechanical ventilation.
Combes A, Hajage D, Capellier G, et al. Extracorporeal membrane oxygenation for severe acute respiratory distress syndrome. N Engl J Med. 2018;378:1965-75.

## Slide 44
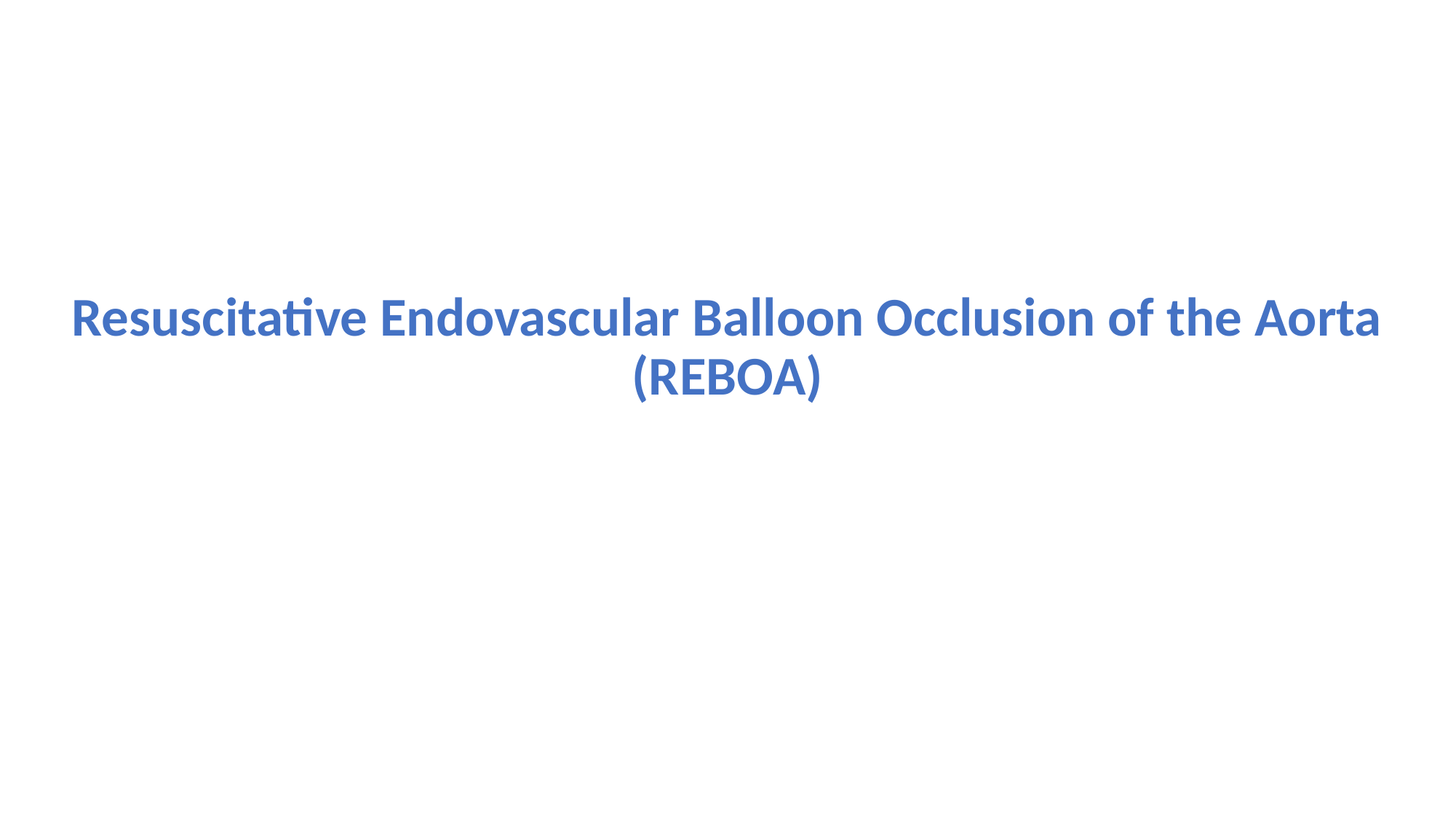

# Resuscitative Endovascular Balloon Occlusion of the Aorta (REBOA)

## Slide 45
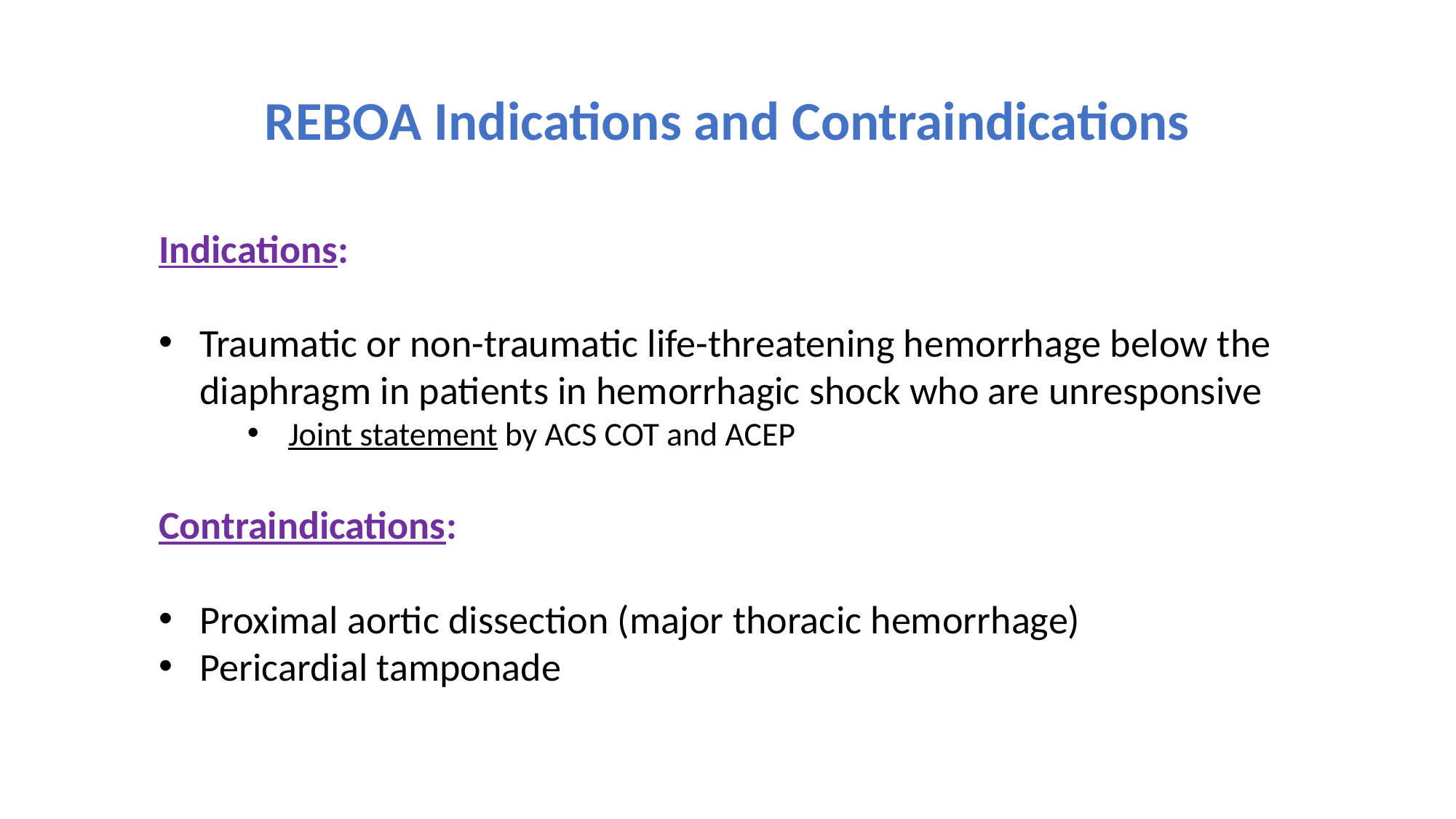

# REBOA Indications and Contraindications
Indications:
Traumatic or non-traumatic life-threatening hemorrhage below the diaphragm in patients in hemorrhagic shock who are unresponsive
Joint statement by ACS COT and ACEP
Contraindications:
Proximal aortic dissection (major thoracic hemorrhage)
Pericardial tamponade

## Slide 46
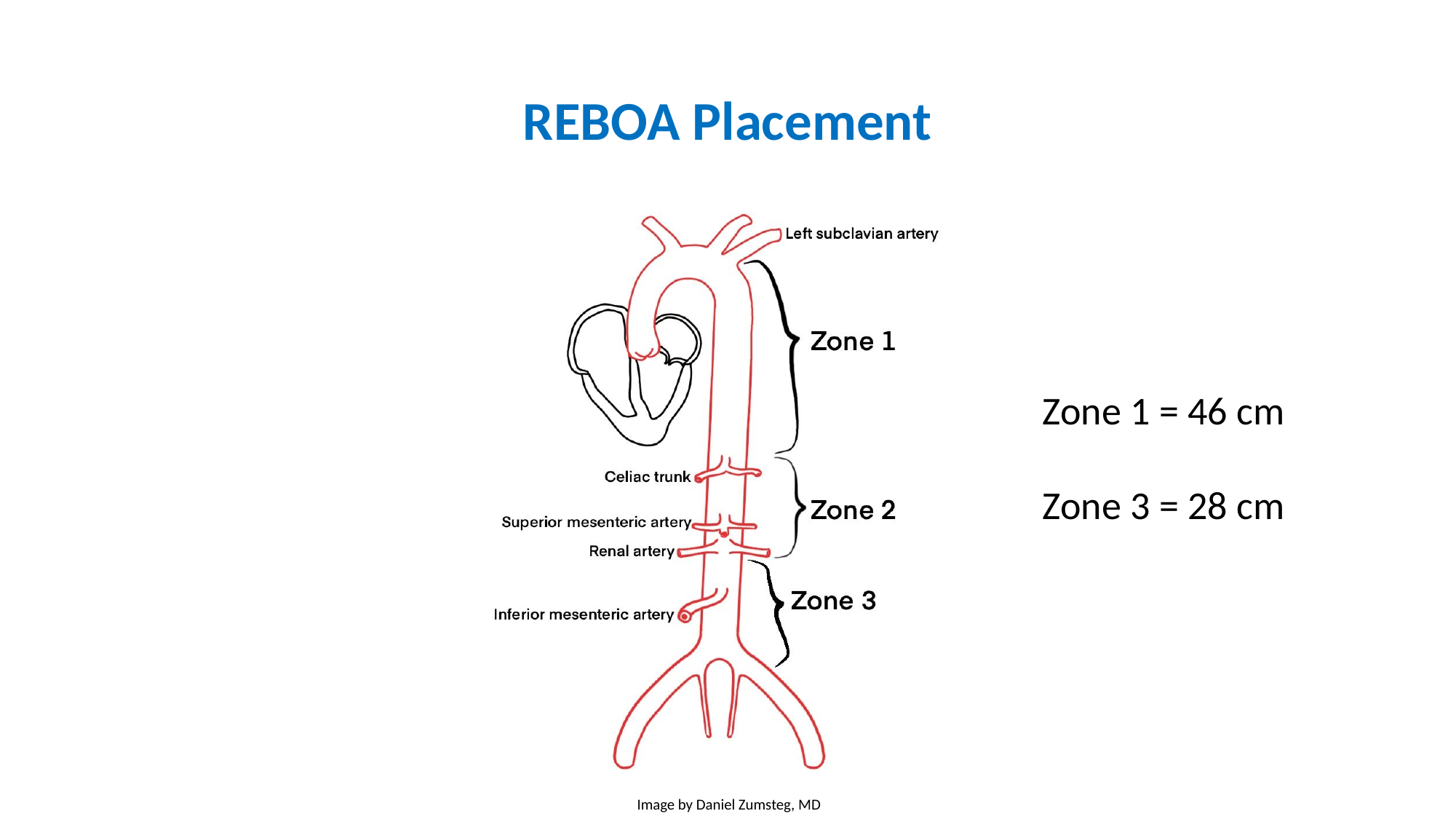

# REBOA Placement
Zone 1 = 46 cm
Zone 3 = 28 cm
Image by Daniel Zumsteg, MD

## Slide 47
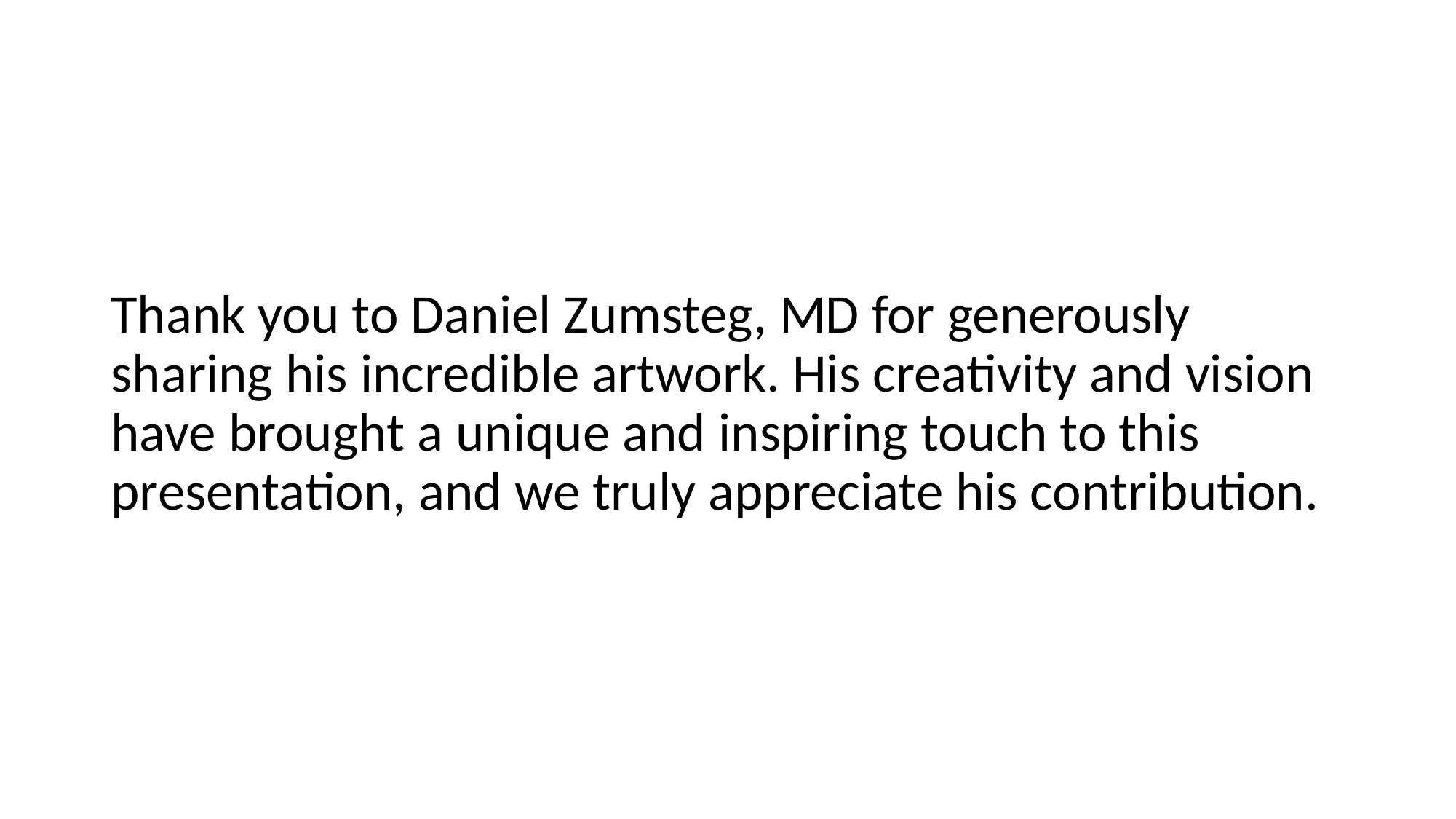

# Thank you to Daniel Zumsteg, MD for generously sharing his incredible artwork. His creativity and vision have brought a unique and inspiring touch to this presentation, and we truly appreciate his contribution.

## Slide 48
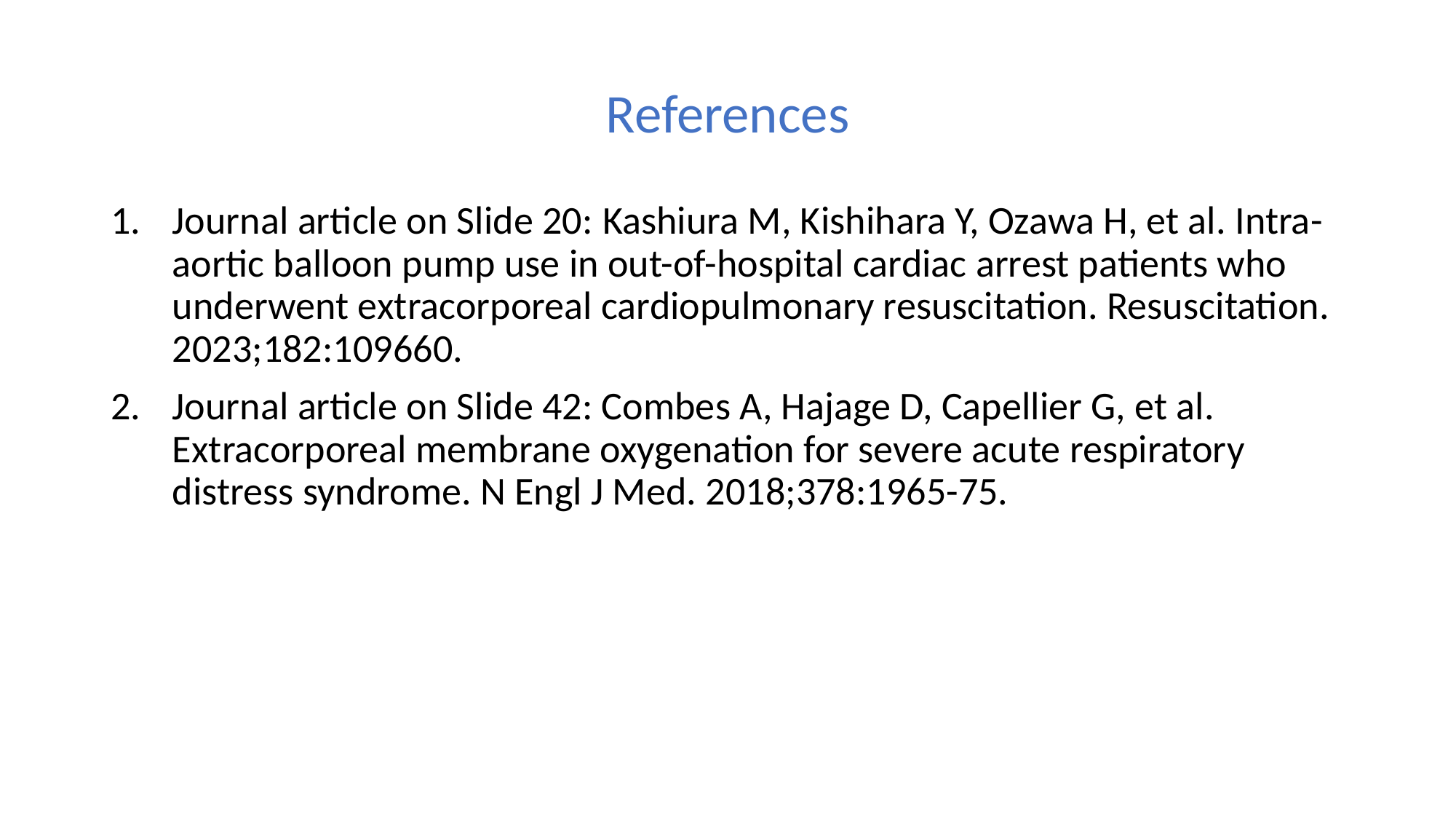

# References
Journal article on Slide 20: Kashiura M, Kishihara Y, Ozawa H, et al. Intra-aortic balloon pump use in out-of-hospital cardiac arrest patients who underwent extracorporeal cardiopulmonary resuscitation. Resuscitation. 2023;182:109660.
Journal article on Slide 42: Combes A, Hajage D, Capellier G, et al. Extracorporeal membrane oxygenation for severe acute respiratory distress syndrome. N Engl J Med. 2018;378:1965-75.

## Slide 49
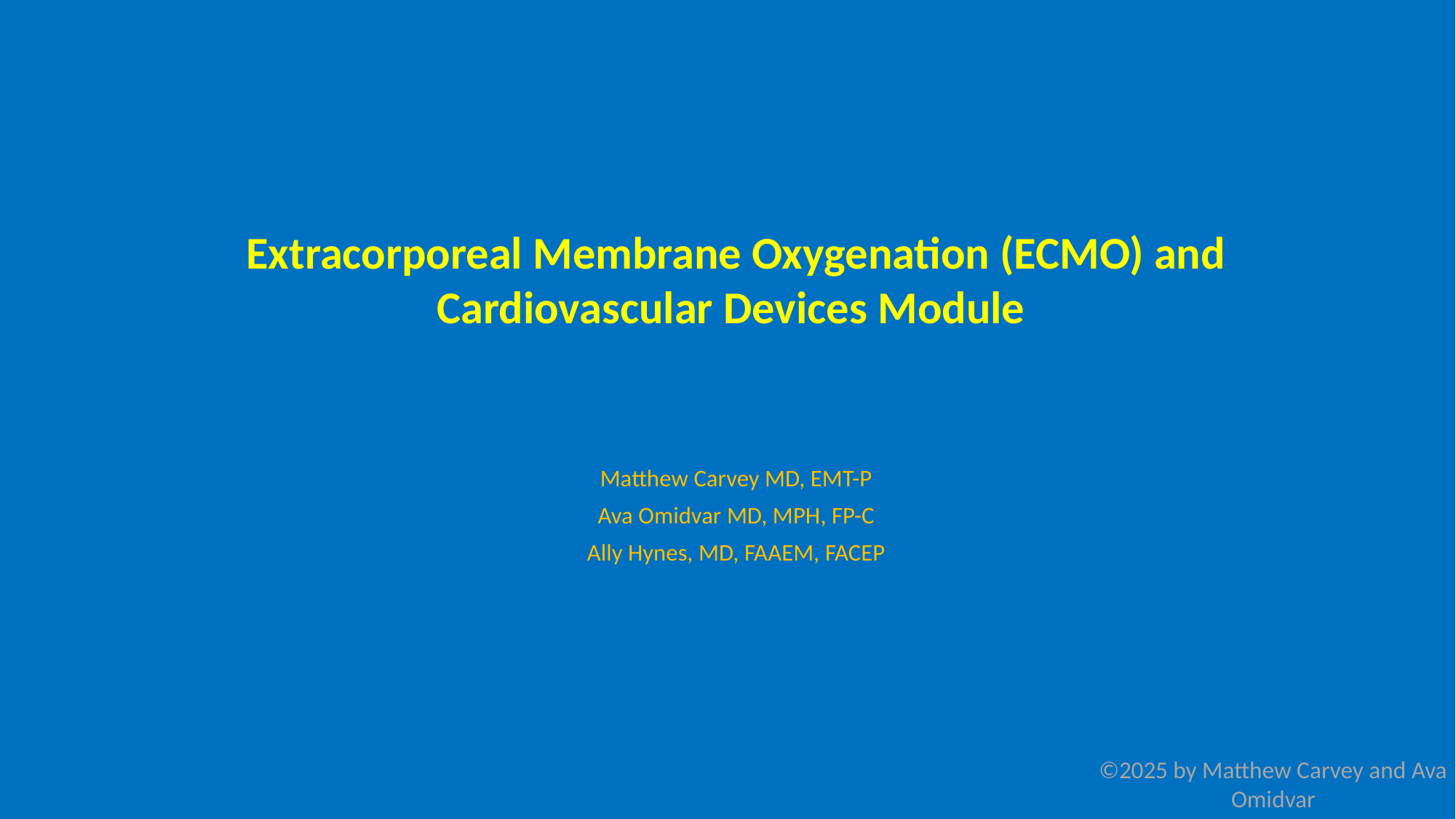

Extracorporeal Membrane Oxygenation (ECMO) and Cardiovascular Devices Module
Matthew Carvey MD, EMT-P
Ava Omidvar MD, MPH, FP-C
Ally Hynes, MD, FAAEM, FACEP
©2025 by Matthew Carvey and Ava Omidvar
