# Supplementary material for: Effectiveness of the Essential Critical Care Concepts in Emergency Medicine: Extracorporeal Membrane Oxygenation and Cardiovascular Devices Module Implementation
Source: MedEdPORTAL. 2025 Nov 7;21:11556. doi: 10.15766/mep_2374-8265.11556 (PMC12592219; doi:10.15766/mep_2374-8265.11556)
Supplement: Supplementary file 1 — Facilitator Guide - ECMO and ACD.docxLearning Objectives - ECMO and ACD.docxModule Presentation Slides - ECMO and ACD.pptxModule Presentation Recording - ECMO and ACD.mp4Module Quiz - ECMO and ACD.docxModule Quiz Answers - ECMO and ACD.docxPostmodule Survey Likert Questions.docx [file mep_2374-8265.11556-s001.zip › E. Module Quiz - ECMO and ACD.docx]

**Extracorporeal Membrane Oxygenation and Cardiovascular Devices Module Quiz**

Q1: Scenario 1: A 59-year-old female is admitted to the hospital post-myocardial infarction. The patient was brought to the ICU and is now exhibiting signs of cardiogenic shock. An intra-aortic balloon pump is inserted. What was the purpose of this procedure?

1. To decrease the workload on the heart
2. To increase myocardial oxygen demand
3. To decrease myocardial oxygen supply
4. To decrease coronary perfusion pressure

Q2: During inflation of the intra-aortic balloon pump, what is being augmented?

1. Systolic blood pressure
2. Peripheral vascular resistance
3. Diastolic blood pressure
4. Systemic vascular resistance

Q3: If inflation of the intra-aortic balloon pump occurs prior to aortic valve closure (dicrotic notch), resulting in decreased diastolic augmentation, what type of timing error is currently occurring?

1. Early inflation
2. Early deflation
3. Late inflation
4. Late deflation
5. There is no timing error

Q4: A patient has been put on extracorporeal membrane oxygenation (ECMO) due to an ejection fraction (EF) of 25% as a bridge to a heart transplant. One cannula is inserted into the subclavian vein and is removing blood from the inferior vena cava, while the other cannula is inserted into the femoral artery and is returning blood to the descending aortic arch. Which form of ECMO is this person receiving?

A. Veno-arterial

B. Veno-venous

Q5: A patient is currently on VA-ECMO. You are checking the system and notice that there are clots collecting within a portion of the device. What is the technical term for the 'portion of the device' in question and where are clots MOST likely to collect?

1. Centrifugal pump
2. Blood pump
3. Membrane oxygenator
4. Sweep gas flow
