# Supplementary material for: Effectiveness of the Essential Critical Care Concepts in Emergency Medicine: Extracorporeal Membrane Oxygenation and Cardiovascular Devices Module Implementation
Source: MedEdPORTAL. 2025 Nov 7;21:11556. doi: 10.15766/mep_2374-8265.11556 (PMC12592219; doi:10.15766/mep_2374-8265.11556)
Supplement: Supplementary file 1 — Facilitator Guide - ECMO and ACD.docxLearning Objectives - ECMO and ACD.docxModule Presentation Slides - ECMO and ACD.pptxModule Presentation Recording - ECMO and ACD.mp4Module Quiz - ECMO and ACD.docxModule Quiz Answers - ECMO and ACD.docxPostmodule Survey Likert Questions.docx [file mep_2374-8265.11556-s001.zip › F. Module Quiz Answers - ECMO and ACD.docx]

**Extracorporeal Membrane Oxygenation, Intra-aortic Balloon Pumps and Cardiovascular Devices Quiz**

Q1: Scenario 1: A 59-year-old female is admitted to the hospital post-myocardial infarction. The patient was brought to the ICU and is now exhibiting signs of cardiogenic shock. An intra-aortic balloon pump is inserted. What was the purpose of this procedure?

1. To decrease the workload on the heart
2. To increase myocardial oxygen demand
3. To decrease myocardial oxygen supply
4. To decrease coronary perfusion pressure

A: This is the correct answer because the two primary goals of an IABP are increasing myocardial oxygen supply and reducing myocardial oxygen demand. This results in decreased workload on the heart by augmenting diastole and systole.

B: The primary goal of an IABP is to decrease myocardial oxygen demand by the mechanism described above, not increase it.

C: The primary goal of an IABP is to increase myocardial oxygen supply by the mechanism described above, not decrease it.

D: The primary goal of an IABP is the increase coronary perfusion pressure through diastolic augmentation and inflation of the IABP during diastole, ultimately assisting with perfusion of the coronary arteries. This results in increased coronary perfusion pressure, not decreased perfusion pressure.

Q2: During inflation of the intra-aortic balloon pump, what is being augmented?

1. Systolic blood pressure
2. Peripheral vascular resistance
3. Diastolic blood pressure
4. Systemic vascular resistance

A: During inflation of the IABP, diastolic augmentation occurs and the coronary arteries are perfused. Systolic blood pressure is not augmented during this time due to this is a period of diastole. If inflation occurs during systole, we result in increased SVR which placed further pressure on the ascending, transverse and early descending aorta.

B: PVR is not being augmented during inflation of the IABP, as inflation occurs during diastole.

C: This is the correct answer, as inflation of the IABP occurs during diastole, which results in diastolic augmentation and further perfusion of the coronary arteries.

D: SVR is not being augmented in the case of an inflating IABP, as this occurs during diastole. If this were to occur during systole, SVR would increase pathologically due to the ejection of blood against an inflated IABP.

Q3: If inflation of the intra-aortic balloon pump occurs prior to aortic valve closure (dicrotic notch), resulting in decreased diastolic augmentation, what type of timing error is currently occurring?

1. Early inflation
2. Early deflation
3. Late inflation
4. Late deflation
5. There is no timing error

A: This is the correct answer, as early inflation of the IABP results in decreased diastolic augmentation due to decreased coronary perfusion pressure. The peak of diastolic augmentation is less affected then that of late deflation. There is an absence of a ‘U’ upstroke from unassisted systole to diastolic augmentation on the waveform.

B: Early deflation is a result of premature deflation of the IABP during diastole. This results in sub-optimal diastolic augmentation. Any deflation error should occur after the dicrotic notch, not before.

C: Late inflation of the IABP occurs markedly after closure of the aortic valve, not before. This results in presence of a dicrotic notch. There is also absence of a sharp “V” or gentle “U” from unassisted systole to diastolic augmentation.

D: Late deflation is inflammation of the IABP while systole is occurring, resulting in ejection of blood against an obstructed system. This results in a widened appearance to diastolic augmentation. Any deflation timing error occurs after the dicrotic notch.

E: The description in the question stem is classic for early inflation. Therefore, a timing error is present.

Q4: A patient has been put on extracorporeal membrane oxygenation (ECMO) due to an ejection fraction (EF) of 25% as a bridge to a heart transplant. One cannula is inserted into the subclavian vein and is removing blood from the inferior vena cava, while the other cannula is inserted into the femoral artery and is returning blood to the descending aortic arch. Which form of ECMO is this person receiving?

A. Veno-arterial

B. Veno-venous

A: Veno-arterial ECMO acts as a temporary replacement for both the heart and lungs. The description states this patient has a poor ejection fraction requiring a heart transplant, or likely a primary cardiac process requiring ECMO which is a key indicator for VA-ECMO. The description of cannulation states a catheter is removing blood from a vein and returning it to an artery. This is another description of VA-ECMO.

B: Veno-venous ECMO is used as a support for a primary respiratory process, such as ARDS. The description given in this question stem describes a primary cardiac process requiring ECMO, and therefore, VA-ECMO is indicated. The description of cannulation also states there is blood coming from a vein and returning to an artery, which defines VA-ECMO.

Q5: A patient is currently on VA-ECMO. You are checking the system and notice that there are clots collecting within a portion of the device. What is the technical term for the 'portion of the device' in question and where are clots MOST likely to collect?

1. Centrifugal pump
2. Blood pump
3. Membrane oxygenator
4. Sweep gas flow

A: The centrifugal pump acts as the forward force or “pump” of the system. Just prior to entering the centrifugal pump, heparin is added to the system to prevent clots from occurring during this process and the centrifugal force also cause further hemolysis. Therefore, it is less likely that clots would collect in this area, nor would they be visible on observation.

B: A blood pump is an alternate name for the centrifugal pump in most situations. There are also other forms of pumps in ECMO devices which may not be centrifugal; however, there is still a less likely chance clots will collect in this area of the ECMO device as heparin is infused just prior to entering the pump.

C: The membrane oxygenator is also known as the “artificial lung” of the ECMO device. Blood is pumped from the centrifugal pump to this section, which provides oxygenation to the system from the O_2_ blender. Due to this portion of the system having lower flow then proximal sections, it is more prone to developing clots. You can also recognize these clots through the transparent glass.

D: Sweep gas flow is the rate of gas flow into the membrane oxygenator. It facilitates removal of CO_2_ from blood and titration of oxygen entering into the system. This portion of the ECMO device is usually not prone to clotting as it solely involves the gases of the system.
