# Supplementary material for: Effectiveness of the Essential Critical Care Concepts in Emergency Medicine: Extracorporeal Membrane Oxygenation and Cardiovascular Devices Module Implementation
Source: MedEdPORTAL. 2025 Nov 7;21:11556. doi: 10.15766/mep_2374-8265.11556 (PMC12592219; doi:10.15766/mep_2374-8265.11556)
Supplement: Supplementary file 1 — Facilitator Guide - ECMO and ACD.docxLearning Objectives - ECMO and ACD.docxModule Presentation Slides - ECMO and ACD.pptxModule Presentation Recording - ECMO and ACD.mp4Module Quiz - ECMO and ACD.docxModule Quiz Answers - ECMO and ACD.docxPostmodule Survey Likert Questions.docx [file mep_2374-8265.11556-s001.zip › G. Postmodule Survey Likert Questions.docx]

**Effectiveness of the Essential Critical Care Concepts in Emergency Medicine: Extracorporeal Membrane Oxygenation and Cardiovascular Devices Module Implementation Post-Module Survey**

Please rank the following statements on a scale of 1 - 5, with a score of 1 being strongly disagree, 3 being neutral and 5 being strongly agree. Circle which answer you believe to be true.

1. This module was relevant to my future career path.

1 2 3 4 5

1. This module was effective in teaching me the basic understanding of IABP’s, ECMO and REBOA as they relate to emergency medicine and critical care.

1 2 3 4 5

1. This module was effective in teaching me how IABP’s, ECMO and REBOA apply to both the emergency medicine and critical care environments.

1 2 3 4 5

1. The flipped-classroom online session helped me solidify my understanding of IABP’s, ECMO and REBOA in the emergency medicine and critical care context.

1 2 3 4 5

1. The flipped-classroom online session facilitated meaningful academic discussion among our group about IABP’s, ECMO and REBOA.

1 2 3 4 5

1. After participating in this module, I am more confident in my ability to manage IABP’s in clinical practice.

1 2 3 4 5

1. After participating in this module, I am more confident in my ability to manage ECMO in clinical practice.

1 2 3 4 5

1. After participating in this module, I am more confident in my ability to understand the use of REBOA in clinical practice.

1 2 3 4 5

1. Do you have any open comments regarding this module?
